# Potential Drivers for the Spatiotemporal Patterns of the Global Burden of Chronic Obstructive Pulmonary Disease Attributable to Ambient Ozone, 1990–2019

**DOI:** 10.3389/ijph.2024.1606062

**Published:** 2024-07-23

**Authors:** Ning Wang, Jian Cheng, Zhiwei Xu, Shuai Wang, Qiutong Wang, Xuefei Feng, Wenbiao Hu, Baohua Wang

**Affiliations:** ^1^ National Center for Chronic and Non-Communicable Diseases Control and Prevention, Chinese Center for Disease Control and Prevention, Beijing, China; ^2^ Department of Epidemiology and Biostatistics, School of Public Health, Anhui Medical University, Hefei, China; ^3^ School of Medicine and Dentistry, Griffith Health, Griffith University, Birtinya, QLD, Australia; ^4^ Affiliated Hospital of Chengde Medical University, Chengde, China; ^5^ School of Public Health and Social Work, Faculty of Health, Queensland University of Technology, Brisbane, QLD, Australia

**Keywords:** chronic obstructive pulmonary disease, ozone, GBD, global warming, green space

## Abstract

**Objectives:**

To identify the long-term spatiotemporal trend of ozone-related chronic obstructive pulmonary disease (COPD) burden by sex and country and to explore potential drivers.

**Methods:**

We retrieved data of ozone-related COPD death and disability adjusted life year (DALY) from the Global Burden of Disease 2019. We used a linear regression of natural logarithms of age-standardized rates (ASRs) with calendar year to examine the trends in ASRs and a panel regression to identify country-level factors associated with the trends.

**Results:**

Global ozone-attributable COPD deaths increased from 117,114 to 208,342 among men and from 90,265 to 156,880 among women between 1990 and 2019. Although ASRs of ozone-related COPD death and DALY declined globally, they increased in low and low-middle Socio-demographic Index (SDI) regions, with faster rise in women. Elevated average maximum temperature was associated with higher ozone-attributable COPD burden, while more green space was associated with lower burden.

**Conclusion:**

More efforts are needed in low and low-middle SDI regions, particularly for women, to diminish inter-country inequality in ozone-attributable COPD. Global warming may exacerbate the burden. Expanding green space may mitigate the burden.

## Introduction

Chronic obstructive pulmonary disease (COPD) led to over three million deaths and 74 million disability-adjusted life years (DALYs) lost in 2019 worldwide, which was the third and fourth most common cause of death or disability, respectively [1]. Population growth and population ageing may further exacerbate the heavy burden of COPD.

Although smoking, occupational exposures and indoor air pollution are identified as major contributors to COPD, ambient air pollution, particularly ozone [2–4], has been increasingly reported to play an important role in the progression of COPD [2, 5–10]. Ozone is a type of gaseous pollutant formed by atmospheric chemical reactions of nitrogen oxides and volatile organic compound precursor under the conditions of high temperature and strong solar radiation. It is therefore the air pollutant that is most sensitive to meteorological factors [11]. Urbanization, industrialization, and together with global warming in the coming decades may aggravate the health impacts of ozone pollution [12]. Despite global efforts to control emissions of ozone precursors, the concentration of ozone remains high, particularly in East Asia where its concentration presents a significant upward trend [13, 14]. In some countries such as China, the concentration of ambient particulate matter has dropped with the stringent control measures, but the ambient ozone pollution remains serious [15–17]. Moreover, unlike particulate matter, which can reduce atmospheric visibility, ozone pollution is more difficult to detect and protect against.

As a strong oxidant, ozone exposure leads to COPD progression through lung inflammation and alveolar epithelial damage [18–20]. A meta-analysis on the relationship between air pollution and COPD showed that pooled relative risk associated with ozone was higher than other air pollutants, and the estimations on ozone-COPD relationship had the highest level of heterogeneity [21]. Disparities in climate, environment, demographic characteristics, and socioeconomic level in different studies may partly explain the heterogeneity of the results. Revealing the spatial and temporal variations of ozone-associated COPD at the national level, and identifying nation-specific factors that modify the effect of ozone exposure will help understand the drivers behind the variations and facilitate interventions for COPD.

However, the existing studies on ozone-related COPD burden have been either carried out in a certain country or region [22, 23], have failed to compare the sex disparity in different countries and regions [24], or have not examined the modification effect of various country-specific characteristics [24–26]. The global burden of disease study (GBD) 2019 uses a unified method to estimate ozone-related COPD burden by sex at the levels of country and region, which allows us to compare the trend in the burden by sex across countries and regions, and to evaluate influencing factors at the level of country by further incorporating socioenvironmental factors [1, 27].

This study aimed to use data from the GBD 2019 to investigate the spatiotemporal trends in COPD mortality and DALY associated with ozone by sex at the regional and national levels. We also aimed to assess whether country-level environment, demographic characteristics, and socioeconomic variables have modified the observed trends.

## Methods

### Data of Ozone-Related COPD Burden

We collected the annual numbers, age-standardized rates (ASRs), and the proportions (accounting for the total burden from COPD) of death and DALY of ozone-attributable COPD by country, region, and sex for the period 1990–2019 from the GBD study results [28]. The GBD 2019 covered 21 regions and 204 countries. Countries were categorized into five groups (high, low, middle, high-middle, and low-middle) according to the Socio-demographic Index (SDI). The SDI was a synthetic metrics that reflected the degree of development associated with health conditions. A higher value in SDI meant a higher level of development [1].

The full details of the GBD methodology had been reported elsewhere [1, 24, 26]. In terms of mortality, data used for estimation included vital registration and surveillance from the cause of death database. COPD deaths were coded as J41-J44, J47 by the *International Classification of Diseases*, 10th version (ICD-10) and 491–492, 496 by the ICD-9. The standard Cause of Death Ensemble model approach was applied to develop mixed effects or spatiotemporal regression models for COPD mortality estimation [5, 24].

In terms of non-fatal health loss, the estimation was based on the data from representative surveys, prevalence studies, and medical claims. COPD was defined by expiratory volume in one second/forced vital capacity (FEV1/FVC) <70% (post-bronchodilator) as per the recommendation of the 2022 Global Initiative for Chronic Obstructive Lung Disease (GOLD) [10]. Data using alternative case-definitions of COPD prevalence (i.e., FEV_1_/FVC<0.7 pre-bronchodilator) were crosswalked to this definition. The severity of COPD was classified as mild, moderate, and severe according to the GOLD criteria. A DisMod-MR 2.1 model was employed to estimate COPD prevalence and the proportions of three GOLD severity groups. These proportions were mapped into the three COPD health states for which disability weights (DWs) were available using the Medical Expenditure Panel Survey data from the United States. The prevalence of each COPD health state was multiplied by its DW and was then adjusted for comorbidity to obtain the years lived with disability (YLD) [5, 25].

Years of life lost (YLLs) were calculated by multiplying the number of deaths at a given age by the standard life expectancy at the corresponding age. DALYs were the sum of YLDs and YLLs [5, 25].

A comparative risk assessment method was used by the GBD 2019 to quantify the burden of disease related to ozone pollution. Population-attributable fraction (PAF) was calculated through exposure data of ambient ozone, relative risk for COPD, and a theoretical minimum risk exposure level (TMREL) for ozone. A chemical transport model based on satellite data was used to estimate ozone exposure. Relative risk was pooled from meta-analysis of cohort studies. The TMREL for ozone was set to a uniform distribution between the lowest and fifth percentile measured by the Cancer Prevention Study-II of American Cancer Society [5, 25]. Ethical approval was not required because we used pre-existing and de-identified data in this study and we did not have any contact with the subjects.

### Data of Potential Country-Level Modifiers

The following country- and year-specific factors that might modify ozone-related COPD were included in the present study according to previous studies [29–33]. Age-standardized prevalence of smoking tobacco use by sex was derived from the GBD 2019 [34]. Demographic and socioeconomic factors including average years of schooling (average number of years people aged 25 years or older participated in formal education), population density, proportion of population aged 65 years or older, proportion of urban population, and gross domestic product (GDP) *per capita* were collected from the Our World in Data [35]. Data in this platform have been widely used in previous studies [36, 37]. We also included environmental factors including average temperature, average maximum temperature, average minimum temperature, rainfall, and Normalized Derived Vegetation Index (NDVI) (the values ranged from 0 to 1 with greater value presenting a higher vegetation coverage). Daily temperature and rainfall were collected from the National Oceanic and Atmospheric Administration of the United States [38]. In consistent with other epidemiological studies [39], we calculated average annual temperature, average annual maximum temperature, and average annual minimum temperature during summer months for each country (June to August for northern hemisphere and December to February for southern hemisphere). We collected NDVI from the National Aeronautics and Space Administration [40] and calculated the annual average.

### Statistical Analysis

We conducted three-step analyses. Step 1: we presented the numbers, ASRs, and proportions of COPD death and DALY attributable to ozone by sex, country, and region with 95% uncertainty intervals (UI). We used maps to show their spatial distributions. Step 2: we fitted the natural logarithm of ASR of ozone-related COPD death and DALY to the calendar year by linear regression with the equation $\ln\left(ASR\right)=\alpha +\beta \times calendar\;year+\varepsilon$ . We calculated annual percentage change (APC) with $\left(\exp\left(\beta \right)-1\right)\times 100$ to identify the trend of ASR. If the APC was significantly higher than 0, we considered that there was a significant increment trend [41]. Step 3: we applied panel regression model to assess the modification effect of country-level factors on ozone-related COPD burden [42]. Because the NDVI data were only available from 2000 to 2018, the panel data analysis was constrained to this period. The model was defined as follows:

$$y_{it}={\beta \times X}_{it}+\varepsilon_{it}$$

where $y_{it}$ is the mortality or DALY rates (all ages) for country *i* in year *t*, $X_{it}$ is the matrix of country-level factors for country *i* in year *t*, *β* is the vector of the parameters, $\varepsilon_{it}$ represents the composite error. Panel data analysis contains three types of models including pooled model, fixed effect model, and random effect model. A pooled model assumes that there is no individual effect and produces consistent parameter estimates as the ordinary least squares (OLS). In fixed effect model, the parameter estimate for the unobserved individual effect (or time effect) is a part of the intercept, while in a random effect model, its estimate is an error component [43, 44]. We used the F test and Hausman test to determine the applicability of the three models and found that the fixed effect estimator was the most suitable for this study [39, 44]. We estimated *β* value and *95% confidence interval (CI)* for each country-level factor.

We performed all analyses with R (version 4.1.0, package of *plm*). A *p-value < 0.05* was viewed as statistically significant.

## Results

### COPD Deaths and DALYs Associated With Ozone by Region

#### Percentage of COPD Deaths and DALYs Associated With Ozone

**FIGURE 1:**
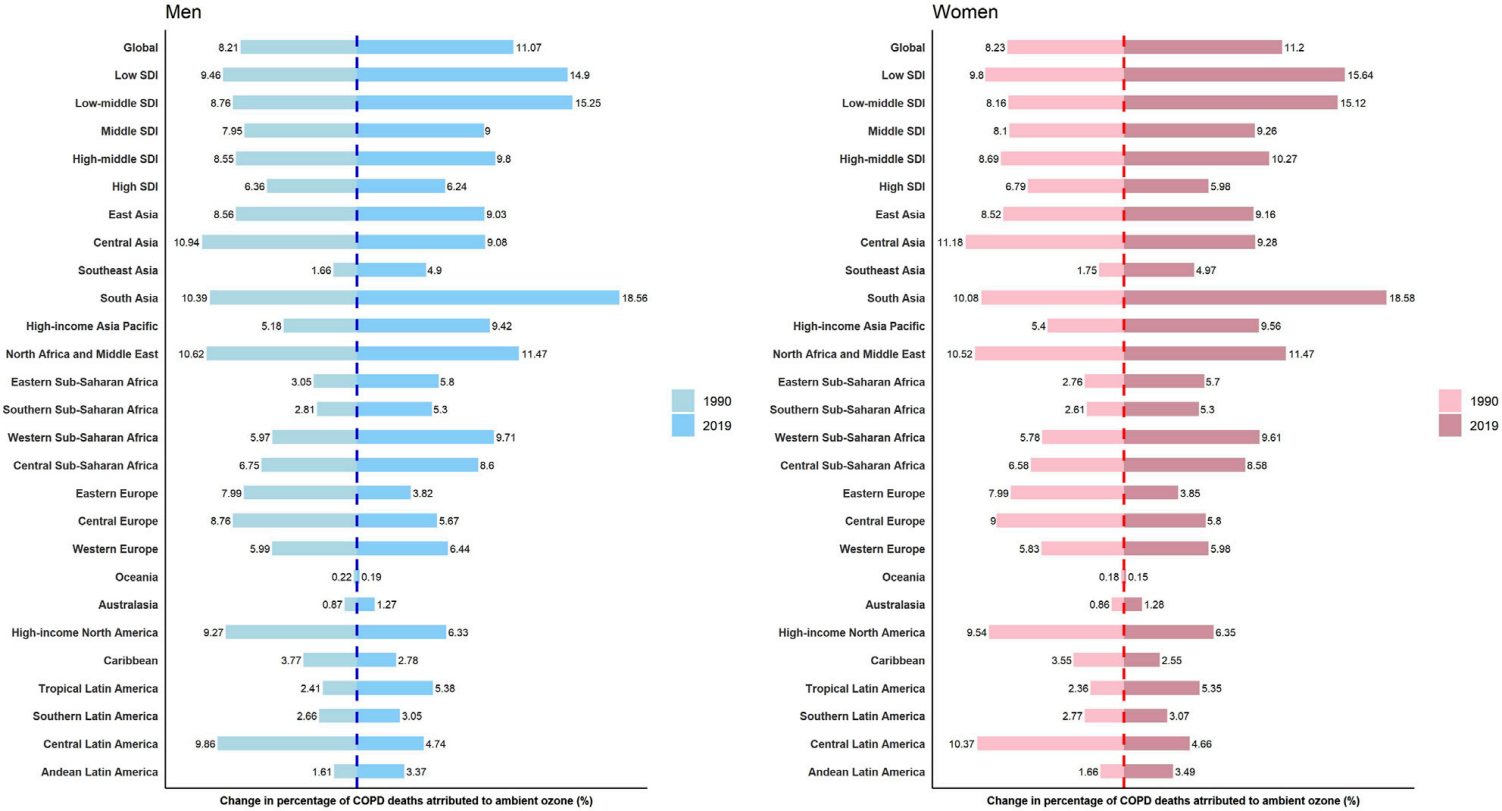
The proportions of chronic obstructive pulmonary disease deaths attributable to ozone among total chronic obstructive pulmonary disease deaths by sex and region (Global, 1990 and 2019).

Ozone pollution contributed to 11.07% and 11.20% of all deaths from COPD among men and women worldwide in 2019, respectively. Across regions with different SDIs, the proportion attributable to ozone pollution increased among both men and women from 1990 to 2019, with the exception of high-SDI region. Among 21 GBD regions, the decrease in the proportion only occurred in seven regions [Central Asia, Eastern Europe, Central Europe, Oceania, High-income North America, Caribbean, and Central Latin America (Figure 1)].

Contribution of ozone pollution to DALYs from COPD was on the rise globally in both men and women during the study period. In addition to the GBD regions where the percentage of ozone-related COPD deaths decreased, a decline in the percentage of ozone-related COPD DALYs occurred in East Asia, North Africa and Middle East, and Western Europe as well (Supplementary Figure S1).

#### Number and ASR of COPD Death Associated With Ozone

The global number of ozone-related COPD deaths increased from 117,114 (95% UI, 51,903 to 187,913) to 208,342 (95% UI, 96,725 to 327,371) among men during the study period. The age-standardized mortality rate (ASMR) of ozone-attributable COPD among men decreased globally, from 8.05 (95% UI, 3.58–12.86) to 6.20 (95% UI, 2.88–9.68) per 100,000 person-years, with an APC of −1.07% (95% CI, −1.34% to −0.80%). However, the ASMR increased in the regions with low [APC 0.74% (95% CI, 0.55, 0.94)] and low-middle SDI [APC 0.61% (95% CI, 0.45, 0.77)]. Among 21 GBD regions, the ASMR among men increased in six regions (Table 1A).

**TABLE 1:** Death numbers and age-standardized mortality rates of chronic obstructive pulmonary disease attributable to ozone by sex and region (Global, 1990 and 2019).

(A) Men					
Regions	Death numbers (UIs)		Age-standardized mortality rates (UIs)		
	1990	2019	1990^a^	2019^a^	Annual percentage change
Global	117,114 (51,903, 187,913)	208,342 (96,725, 327,371)	8.05 (3.58, 12.86)	6.20 (2.88, 9.68)	−1.07% (−1.34, −0.80)
Region by socio-demographic index					
Low	8,127 (3,625, 13,212)	21,460 (9,668, 33,111)	9.43 (4.18, 15.33)	11.75 (5.31, 18.09)	0.74% (0.55, 0.94)
Low-middle	29,581 (12,793, 47,163)	83,494 (39,013, 131,166)	13.02 (5.61, 20.93)	15.88 (7.4, 24.81)	0.61% (0.45, 0.77)
Middle	38,000 (16,850, 61,897)	54,981 (25,293, 87,368)	10.82 (4.79, 17.55)	6.08 (2.81, 9.6)	−1.97% (−2.52, −1.41)
High-middle	31,470 (13,981, 50,811)	33,615 (15,684, 52,368)	8.99 (4.01, 14.55)	4.42 (2.06, 6.9)	−2.82% (−3.16, −2.47)
High	9,924 (4,484, 16,247)	14,764 (6,412, 24,545)	2.49 (1.13, 4.08)	1.67 (0.72, 2.77)	−1.59% (−1.88, −1.31)
GBD region					
East Asia	54,774 (24,039, 89,180)	55,572 (24,795, 88,720)	22.06 (9.76, 35.54)	8.28 (3.71, 13.14)	−3.2% (−3.86, −2.54)
Central Asia	980 (445, 1,530)	907 (418, 1,432)	6.39 (2.9, 9.98)	4.25 (1.94, 6.72)	−1.86% (−2.26, −1.46)
Southeast Asia	1,197 (402, 2,347)	6,154 (2,712, 10,483)	1.36 (0.46, 2.66)	3.02 (1.33, 5.13)	2.65% (1.94, 3.37)
South Asia	34,762 (15,393, 55,336)	110,087 (52,493, 171,738)	16.74 (7.43, 26.92)	20.18 (9.57, 31.18)	0.53% (0.32, 0.74)
High-income Asia Pacific	802 (336, 1,376)	2,978 (1,288, 4,879)	1.22 (0.51, 2.1)	1.32 (0.57, 2.15)	−0.29% (−0.67, 0.09)
North Africa and Middle East	2,864 (1,300, 4,529)	6,565 (3,020, 10,366)	4.34 (1.96, 6.85)	3.75 (1.72, 5.93)	−0.23% (−0.41, −0.04)
Eastern Sub-Saharan Africa	490 (189, 910)	1,368 (588, 2,331)	1.68 (0.65, 3.14)	2.48 (1.06, 4.17)	1.49% (0.24, 2.76)
Southern Sub-Saharan Africa	150 (57, 275)	460 (201, 767)	1.62 (0.61, 2.95)	2.6 (1.13, 4.37)	1.72% (0.87, 2.58)
Western Sub-Saharan Africa	785 (322, 1,371)	2,189 (924, 3,612)	2.46 (1.01, 4.25)	3.31 (1.4, 5.43)	1.39% (0.46, 2.34)
Central Sub-Saharan Africa	323 (129, 563)	671 (271, 1,182)	4.27 (1.69, 7.38)	4.25 (1.71, 7.37)	0.33% (−0.83, 1.51)
Eastern Europe	4,329 (1,835, 6,970)	1,284 (501, 2,233)	5.57 (2.37, 8.95)	1.1 (0.44, 1.93)	−6.18% (−6.63, −5.72)
Central Europe	2,425 (1,091, 3,855)	1,359 (591, 2,283)	4.51 (2.03, 7.18)	1.56 (0.68, 2.62)	−3.55% (−3.89, −3.2)
Western Europe	5,807 (2,619, 9,546)	7,660 (3,446, 12,568)	2.71 (1.22, 4.46)	1.75 (0.79, 2.87)	−1.66% (−1.93, −1.4)
Oceania	4 (1, 9)	6 (2, 12)	0.39 (0.09, 0.93)	0.27 (0.08, 0.55)	−2.44% (−3.25, −1.63)
Australasia	41 (13, 85)	79 (26, 157)	0.45 (0.14, 0.94)	0.33 (0.11, 0.65)	−1.71% (−2.46, −0.94)
High-income North America	5,267 (2,409, 8,293)	6,644 (2,863, 11,069)	3.64 (1.66, 5.74)	2.3 (0.99, 3.84)	−1.73% (−2.18, −1.28)
Caribbean	103 (41, 187)	173 (62, 327)	0.92 (0.37, 1.65)	0.74 (0.26, 1.4)	−1.39% (−2.12, −0.65)
Tropical Latin America	523 (195, 958)	2,038 (848, 3,462)	1.6 (0.59, 2.95)	2.14 (0.89, 3.66)	−0.23% (−1.03, 0.59)
Southern Latin America	191 (72, 355)	387 (149, 704)	1.09 (0.41, 2.02)	1.13 (0.43, 2.04)	−0.07% (−0.37, 0.22)
Central Latin America	1,263 (615, 1905)	1,613 (678, 2,775)	4.05 (1.96, 6.14)	1.65 (0.7, 2.84)	−3.73% (−4.02, −3.43)
Andean Latin America	33 (12, 63)	147 (58, 269)	0.39 (0.14, 0.75)	0.6 (0.24, 1.1)	1.49% (0.42, 2.56)

(B) Women					
Regions	Death numbers (UIs)		Age-standardized mortality rates (UIs)		
	1990	2019	1990^a^	2019^a^	Annual percentage change
Global	90,265 (40,644, 146,693)	156,880 (73,265, 249,792)	4.56 (2.06, 7.42)	3.57 (1.67, 5.68)	−1.15% (−1.41, −0.89)
Region by socio-demographic index					
Low	5,055 (2,142, 8,622)	16,879 (7,933, 27,039)	5.74 (2.44, 9.85)	8.45 (3.93, 13.56)	1.3% (1.11, 1.49)
Low-middle	17,986 (7,841, 29,815)	63,389 (29,204, 102,294)	8 (3.49, 13.47)	10.51 (4.82, 16.96)	0.71% (0.52, 0.9)
Middle	34,243 (15,122, 56,277)	39,746 (18,063, 65,160)	8.45 (3.72, 13.75)	3.61 (1.65, 5.9)	−3.05% (−3.6, −2.49)
High-middle	26,131 (11,322, 43,595)	24,716 (11,167, 40,975)	4.7 (2.04, 7.85)	2.06 (0.93, 3.42)	−3.38% (−3.75, −3)
High	6,842 (3,022, 11,327)	12,133 (5,090, 20,439)	1.04 (0.46, 1.71)	0.96 (0.41, 1.61)	−0.47% (−0.91, −0.04)
GBD region					
East Asia	53,320 (23,096, 89,695)	42,095 (19,157, 71,915)	16.11 (7.04, 26.58)	4.42 (2.01, 7.55)	−4.52% (−5.21, −3.82)
Central Asia	769 (341, 1,247)	669 (311, 1,085)	2.95 (1.31, 4.8)	2.09 (0.97, 3.35)	−1.6% (−1.97, −1.22)
Southeast Asia	755 (251, 1,512)	3,158 (1,329, 5,417)	0.71 (0.24, 1.42)	1.17 (0.49, 1.99)	1.53% (0.84, 2.22)
South Asia	19,608 (8,422, 33,440)	84,834 (39,424, 135,381)	10.64 (4.56, 18.13)	14.68 (6.76, 23.47)	0.83% (0.58, 1.09)
High-income Asia Pacific	479 (194, 824)	1,673 (729, 2,830)	0.45 (0.18, 0.78)	0.43 (0.18, 0.72)	−0.87% (−1.16, −0.57)
North Africa and Middle East	1,704 (747, 2,852)	3,749 (1,744, 5,937)	2.58 (1.13, 4.28)	2.24 (1.04, 3.57)	−0.22% (−0.46, 0.02)
Eastern Sub-Saharan Africa	224 (78, 442)	769 (313, 1,391)	0.73 (0.26, 1.43)	1.14 (0.47, 2.05)	1.74% (0.39, 3.1)
Southern Sub-Saharan Africa	95 (36, 185)	330 (142, 563)	0.7 (0.27, 1.36)	1.17 (0.5, 2)	2.15% (1.3, 3.02)
Western Sub-Saharan Africa	459 (195, 791)	1,250 (563, 2,096)	1.23 (0.52, 2.1)	1.62 (0.72, 2.68)	1.25% (0.28, 2.22)
Central Sub-Saharan Africa	233 (82, 485)	694 (256, 1,577)	2.91 (1.04, 6.02)	3.3 (1.22, 7.62)	0.89% (−0.29, 2.09)
Eastern Europe	2,682 (977, 4,579)	720 (278, 1,361)	1.51 (0.55, 2.59)	0.3 (0.12, 0.56)	−5.99% (−6.24, −5.74)
Central Europe	1,235 (548, 2003)	856 (364, 1,447)	1.53 (0.68, 2.47)	0.62 (0.26, 1.03)	−2.99% (−3.33, −2.65)
Western Europe	3,186 (1,389, 5,399)	5,590 (2,348, 9,390)	0.83 (0.36, 1.39)	0.8 (0.34, 1.35)	−0.08% (−0.35, 0.2)
Oceania	2 (0, 5)	4 (1, 8)	0.21 (0.05, 0.54)	0.15 (0.05, 0.32)	−2.34% (−3.29, −1.39)
Australasia	20 (6, 43)	66 (21, 135)	0.15 (0.05, 0.31)	0.21 (0.07, 0.43)	0.67% (0.03, 1.31)
High-income North America	3,973 (1,845, 6,402)	6,769 (2,654, 11,660)	1.79 (0.83, 2.89)	1.74 (0.69, 2.98)	−0.21% (−0.86, 0.44)
Caribbean	62 (24, 121)	113 (39, 223)	0.49 (0.19, 0.96)	0.4 (0.14, 0.79)	−1.36% (−2.04, −0.66)
Tropical Latin America	330 (119, 614)	1,698 (709, 2,923)	0.84 (0.31, 1.57)	1.28 (0.54, 2.21)	0.25% (−0.62, 1.12)
Southern Latin America	103 (38, 193)	307 (113, 562)	0.42 (0.16, 0.8)	0.58 (0.22, 1.07)	0.88% (0.59, 1.17)
Central Latin America	996 (471, 1,531)	1,409 (566, 2,415)	2.89 (1.38, 4.46)	1.15 (0.46, 1.97)	−3.68% (−3.99, −3.37)
Andean Latin America	27 (10, 54)	130 (53, 237)	0.31 (0.11, 0.61)	0.46 (0.19, 0.84)	1.35% (0.23, 2.48)

Abbreviations: UI, uncertainty interval; GBD, global disease burden; SDI, socio-demographic index.

^a^ Per 100,000 person-years.

The number of ozone-related COPD deaths in women was constantly lower than that in men across regions with different SDIs. The death number among women increased from 90,265 (95% UI, 40,644 to 146,693) to 156,880 (95% UI, 73,265 to 249,792) globally from 1990 to 2019. The ASMR of ozone-attributable COPD in women also decreased globally, from 4.56 (95% UI, 2.06–7.42) to 3.57 (95% UI, 1.67–5.68) per 100,000 person-years, with an APC of −1.15% (95% CI, −1.41% to −0.89%). Women in the regions with low [APC 1.30% (95% CI, 1.11, 1.49)] and low-middle SDI [APC 0.71% (95% CI, 0.52, 0.90)] also experienced an increased ASMR, with the APC higher than men in corresponding regions. Among 21 GBD regions, the ASMR among women increased in eight regions [Table 1B].

#### Number and ASR of COPD DALY Associated With Ozone

Global numbers of ozone-attributable COPD DALYs increased among both men and women from 1990 to 2019, whereas the age-standardized DALY rates (ASDRs) decreased significantly among both sexes. Nevertheless, the ASDR still increased in the regions with low and low-middle SDI. Among 21 GBD regions, the increments in ASDR in men and women were observed in four and five regions, respectively (Supplementary Table S1).

### COPD Deaths and DALYs Associated With Ozone by Country

#### Percentage of COPD Deaths and DALYs Associated With Ozone

**FIGURE 2:**
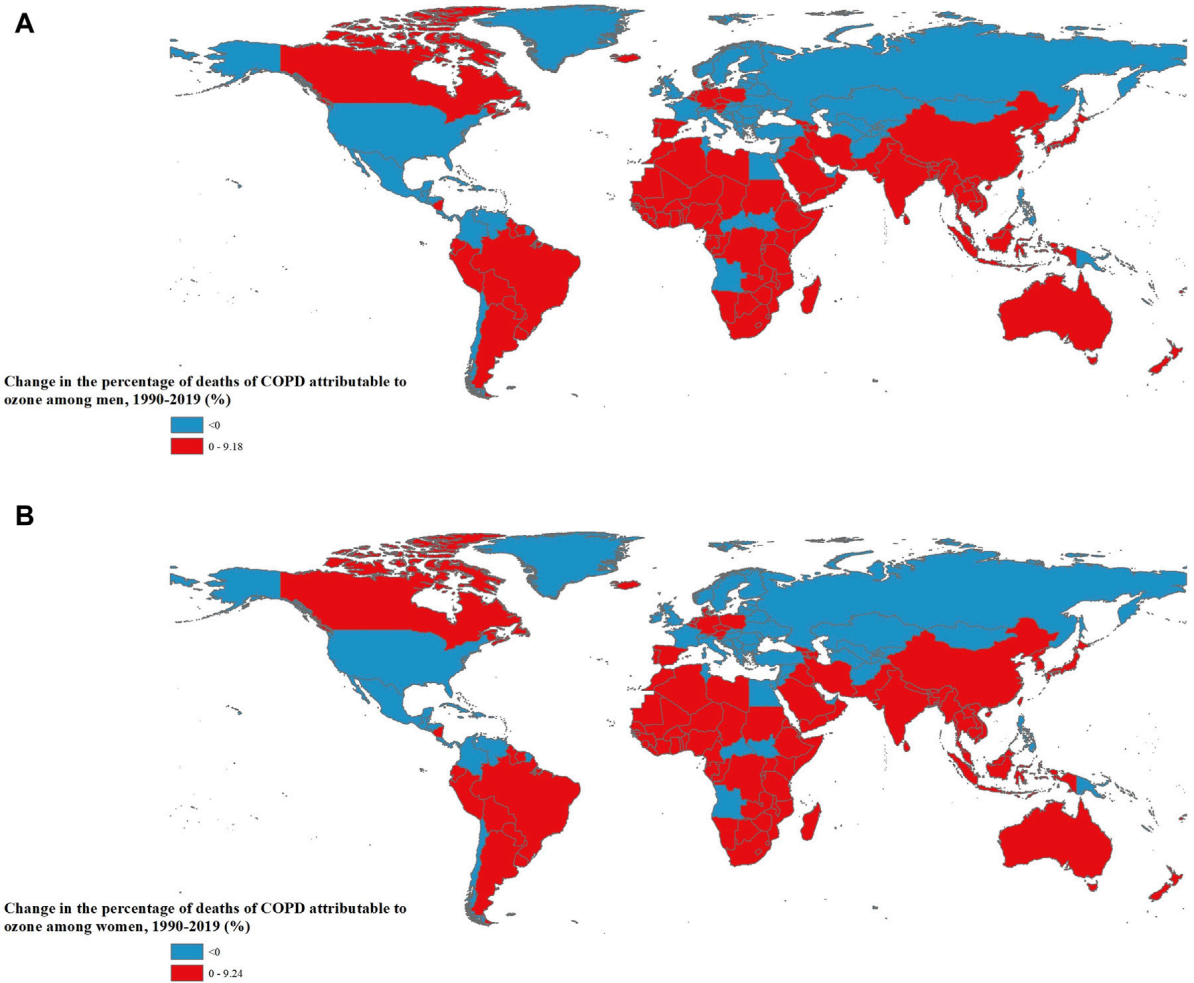
The change in the proportions of chronic obstructive pulmonary disease deaths attributable to ozone among total chronic obstructive pulmonary disease deaths by sex and country [**(A)**: Men; **(B)**: Women] (Global, 1990 to 2019).

The percentages of ozone-attributable COPD deaths increased in both men and women in 118 countries of 204 countries (57.8%, n = 118/204). The highest increase occurred in Bangladesh, Maldives, and India (Figure 2; Supplementary Table S2). The percentages of ozone-attributable COPD DALYs in men and women increased in 109 (53.4%, n = 109/204) and 107 countries (52.5%, n = 107/204) out of 204 countries, respectively. The highest increase occurred in India, Bangladesh, and Nepal among men, and in India, Maldives, and Bangladesh among women (Supplementary Figure S2; Supplementary Table S3).

#### Number and ASR of COPD Death Associated With Ozone

**FIGURE 3:**
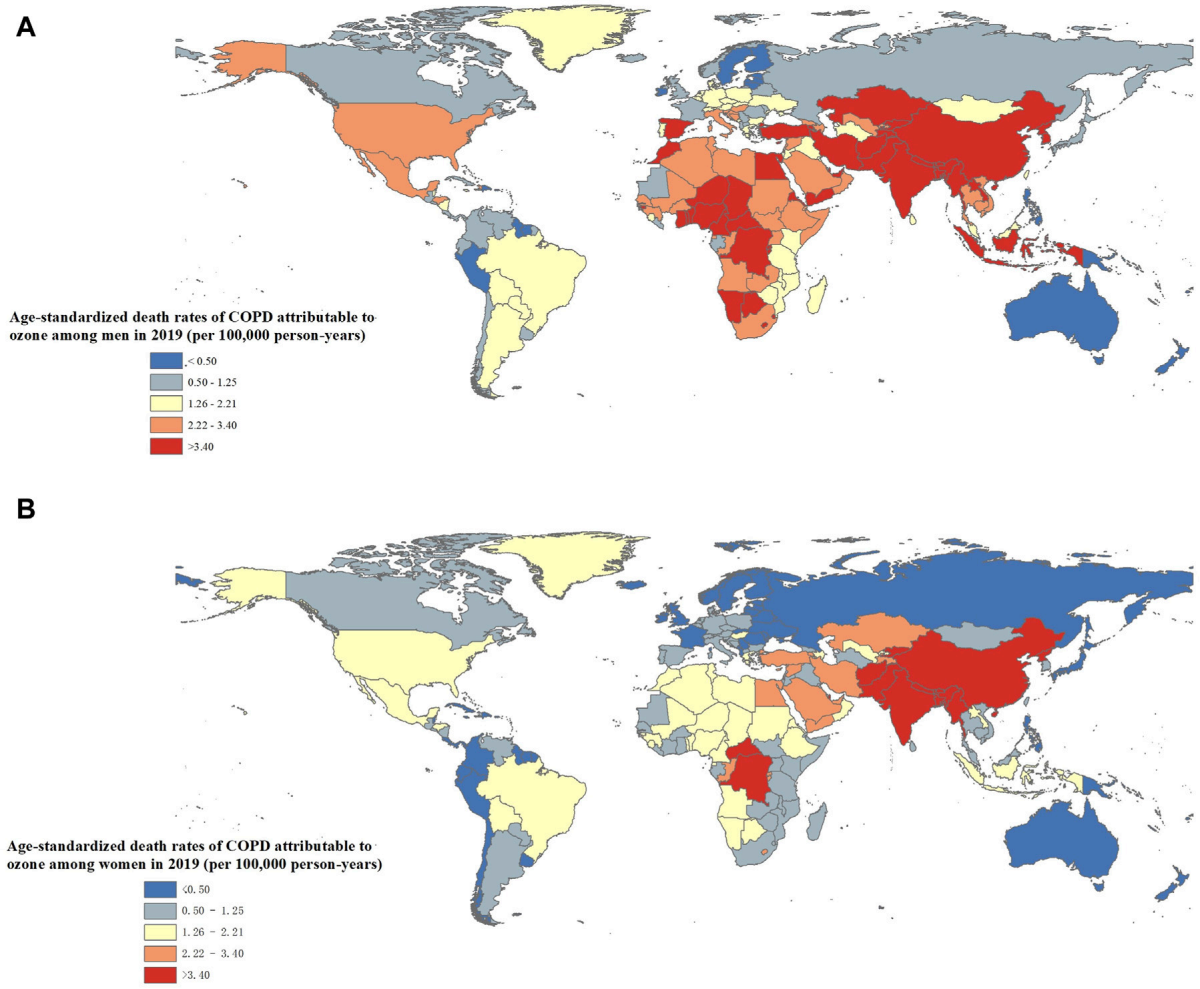
The age-standardized death rates of chronic obstructive pulmonary disease attributable to ozone by sex and country [**(A)**: Men; **(B)**: Women] (Global, 2019).

Among 204 countries and territories, Nepal, India, and Pakistan had the highest ASMR of ozone-attributable COPD in men in 2019, and Nepal, India, and Bhutan had the highest value in women (Figure 3; Supplementary Table S2). The number of COPD-related deaths in men was the highest in India, China, and Pakistan in 2019, while the number in women was the highest in India, China, and United States (Supplementary Table S2).

**FIGURE 4:**
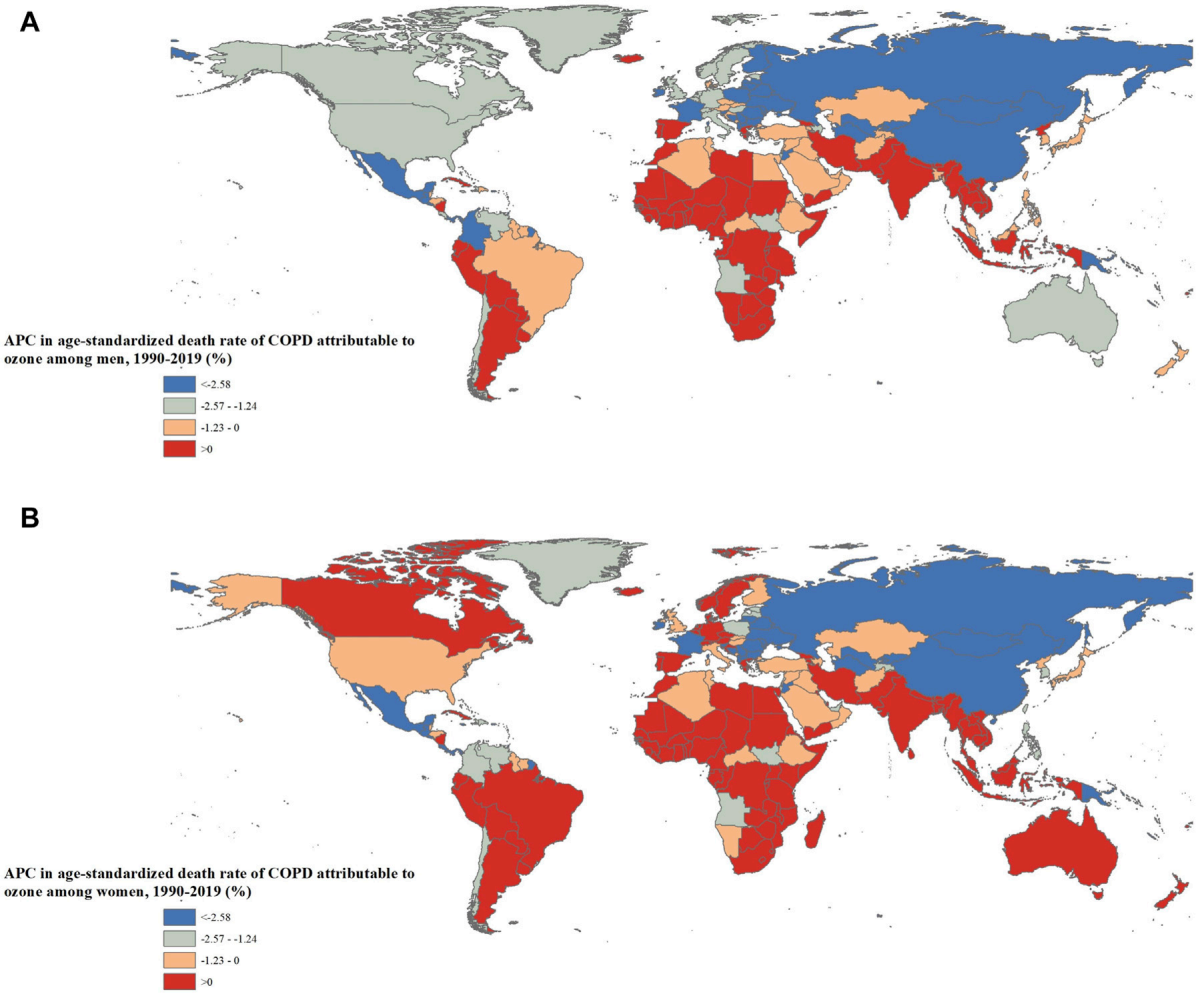
Annual percentage change in the age-standardized death rates of chronic obstructive pulmonary disease attributable to ozone by sex and country [**(A)**: Men; **(B)**: Women] (Global, 1990 to 2019).

Among 204 countries, the ASMR of ozone-attributable COPD in men significantly increased in 59 countries out of 204 countries (28.9%, n = 59/204), with Madagascar, Mozambique, and Kenya having the highest annual growth. The ASMR in women significantly increased in 71 countries (34.8%, n = 71/204), with Madagascar, Seychelles, and Kenya having the highest annual growth (Figure 4; Supplementary Table S2).

#### Number and ASR of COPD DALY Associated With Ozone

The DALY numbers and ASDRs of ozone-attributable COPD by sex and country are shown in Supplementary Table S3. The distribution (Supplementary Figure S3) and trend of (Supplementary Figure S4) ozone-attributable COPD DALY were similar to the death.

### Country-Level Factors Associated With Ozone-Related COPD

The patterns of the significant associations between country-level factors and ozone-attributable COPD burden were similar in terms of death and DALY in both men and women (Table 2; Supplementary Table S4). Smoking prevalence was positively associated with the rate of the burden. With regards to demographic factors, average years of schooling was negatively associated with the burden. A higher population density and a higher percentage of population aged ≥65 years old were associated with a higher ozone-related COPD burden. In terms of socioeconomic factors, countries with a higher percentage of urban population were more likely to have a lighter ozone-attributable COPD burden. Among meteorological factors, average maximum and minimum temperature and rainfall were positively associated with ozone-related COPD burden. In contrast, NVDI was associated with significantly decreased ozone-attributable COPD deaths and DALYs in both men and women.

**TABLE 2:** Association between country-level factors and mortality rate of chronic obstructive pulmonary disease attributable to ozone pollution (Global, 1990 to 2019).

Country-level factors	Men			Women		
	β	95% CI	*p*-value	β	95% CI	*p*-value
Lifestyle factors						
Smoking prevalence (%)	0.042	0.036, 0.048	<0.001	0.03	0.021, 0.039	<0.001
Demographic factors						
Average years of schooling (years)	−0.152	−0.190, −0.113	<0.001	−0.052	−0.084, −0.021	0.001
Population density (thousand people/km^2^)	0.221	0.095, 0.347	<0.001	0.129	0.025, 0.233	0.02
Proportion of population aged 65 years and above (%)	0.215	0.193, 0.236	<0.001	0.109	0.089, 0.129	<0.001
Socioeconomic factors						
Proportion of urban population (%)	−0.018	−0.023, −0.013	<0.001	−0.017	−0.021, −0.013	<0.001
GDP *per capita* [US$ (thousand)]	0.003	−0.002, −0.008	0.26	0.002	−0.003, 0.006	0.48
Environmental factors						
Average maximum temperature (°C)	0.169	0.078, 0.259	<0.001	0.108	0.034, 0.183	0.004
Average temperature (°C)	−0.236	−0.388, −0.08	0.002	−0.201	−0.327, −0.075	0.002
Average minimum temperature (°C)	0.085	0.007, 0.163	0.03	0.104	0.038, 0.169	0.002
Rainfall (mm)	0.001	0.001, 0.002	<0.001	0.001	0.0003, 0.001	0.002
NVDI	−25.199	−29.652, −20.746	<0.001	−21.859	−25.611, −18.107	<0.001

## Discussion

Ozone is a worldwide threat to COPD, but there is limited evidence detailing how ozone-related COPD burden changed spatially and temporally by sex at the national level and what the underlying drivers were. In this study, we reported that the trends in the ASRs of ozone-attributable COPD death and DALY showed a great spatial variation, with low and low-middle SDI regions having a significant increasing trend. It’s worth noting that such increasing trend was more marked among women than men. More importantly, we found that such variation could be related to a variety of country-level environmental and socioeconomic characteristics, suggesting that tailored strategies taking into account these characteristics are crucial for ozone-attributable COPD prevention and control.

The GBD study 2015 reported that 254,000 deaths from COPD were associated with exposure to ozone worldwide in 2015, representing 8.0% of total COPD deaths [45]. Our study found that more than 364,000 COPD deaths (over 208,000 for men and 156,000 for women) were associated with exposure to ozone in 2019, accounting for around 11.0% of total COPD deaths. This raise may be due to the increases in both levels of ozone and COPD mortality and the updated estimation method. Although some studies have initially described ozone-related COPD burden based on the GBD 2019 [24–26], our study further detailed the spatial and temporal trends in the number, percentage, and ASR of ozone-related COPD death and DALY by sex in each country, and for the first time assessed the modifying effects of a variety of country-level environmental and socioeconomic factors on such trends [24].

Our study found that although the ASRs of ozone-attributable COPD death and DALY declined worldwide, the ASRs in regions of low SDI and low-middle SDI were still on the rise. Nepal, India, Pakistan, and Bhutan in particular showed the highest ASMR attributable to ozone pollution. With rapid economic development, these regions are experiencing the most severe ambient air pollution in the world [46]. Relatively low socioeconomic level may be another reason for the higher ASMR in these regions because poverty has been reported to be associated with airflow obstruction [47, 48]. Moreover, the relatively poor treatment and health service may further aggravate the mortality burden from COPD in less developed countries as health resources have been mainly allocated to infectious diseases rather than non-communicable diseases such as COPD. In this study, we also found that country-level socioeconomic indicator, i.e., proportion of urban population, was negatively associated with the mortality rate of COPD through panel analysis, which is able to reflect, to some extent, the negative relationship between socioeconomic level and COPD mortality. These findings revealed a huge inequity in the ozone-attributable COPD burden among countries with different SDI levels. Due to limited resources, countries with low and low-middle SDI were facing the dilemma of economic development and air pollution control. More international efforts are needed to decrease ozone pollution exposure and related health disparities.

We found that the absolute numbers and ASRs of COPD burden were higher among men than women, which is consistent with previous studies [5]. However, it is worth noting that the increase in ASMR among women (34.8% of all countries) was observed in more countries compared to men (28.9% of all countries) from 1990 to 2019. The ASMR in low and low-middle SDI regions increased more rapidly among women than men (higher absolute values of APC in women than men), and the ASMR in high SDI region decreased more slowly among women than men (lower absolute value of APC in women than men). This may indicate that women are more susceptible to the process of air quality deterioration. Physiological differences in hormone, lung volume, deposition, and reactivity may partly explain the disparity in susceptibility between men and women [49, 50]. Another possible examination is the disparity in the trend of smoking prevalence between men and women. It has been reported that smoking among men decreased more dramatically than that among women in high-income countries from 2007 to 2019 [51]. Smoking and ozone exposure may have joint effect on COPD mortality. Our finding suggests that more attention should be paid to women in preventing the influence of ozone on respiratory system. More refined data collection and analysis is needed to discern the reasons for the disparity in response to ozone between men and women.

We further evaluated potential modifiers on ozone-related COPD death and DALY at the national level using panel analyses. Countries with a higher smoking prevalence were found to have higher rates of COPD mortality and DALY, suggesting that smoking may have synergistic effect with air pollution [52]. With the implementation of multiple tobacco control initiatives such as the World Health Organization Framework Convention on Tobacco Control (WHO FCTC) and the MPOWER measures, smoking prevalence had decreased worldwide during the last three decades, but the absolute number of smokers continued to increase [51, 53]. Moreover, a wide implementation gap for tobacco control policies still existed among countries and only two countries (Brazil and Turkey) have fully applied all MPOWER measures [46, 53]. Strong and extensive implementation of evidence-based interventions is needed to speed up reduction in health influence of smoking.

Our results show population density and percentage of population aged ≥65 years old were positively related with ozone-attributable COPD mortality and DALY rates, which indicates that COPD burden will continue to elevate with global population growth and population ageing. Average years of schooling and proportion of urban population that reflect the socioeconomic levels were found to be negatively associated with the rates of ozone-attributable COPD mortality and DALY. The possible explanation is that countries with higher socioeconomic levels tend to implement better early warning and protective measures against air pollution and be able to afford better healthcare services [32].

Our study found that countries with higher average maximum and minimum temperatures were likely to have higher ozone-attributable COPD mortality and DALY rates. Previous studies also discovered that temperature synergistically modified the ozone-mortality [54]. Our study provides the evidence of this synergistic effect through analysis across multiple countries worldwide. The ozone-attributable COPD burden is predicted to surge further as climate change becomes more fiercer and heat waves become more frequent. The coordinated efforts in early warning and protection against climate change and ozone pollution are needed to reduce the synergistic effect.

It is noticeable that our study found increased greenness could attenuate the susceptibility of COPD death and DALY related to ozone pollution. Most previous studies on green space and respiratory diseases focused on its direct protective effect [55–58]. Only two studies revealed a favorable modification effect of greenness on the association between ozone pollution and respiratory diseases [59, 60]. The underlying reasons for the modification effect may include the differences in health behaviors and stress reduction in regions of different greenness. For example, people live in a greener space may be more likely to take protection measures against ozone pollution and lead a healthier lifestyle (i.e., less likely to smoke), and may be more likely to be able to recover from psychological stress [61, 62]. This finding suggests the importance of enlarging green space in improving resiliency from the health influence of ozone pollution. It also indicates that future studies on the health effect of air pollution should take the modification effect of greenness into account.

Our study has several novel findings with public health significance. First, the marked disparity in the trend of ASR of ozone-attributable COPD death and DALY across regions with different SDI underscores the urgency to allocate more resources in regions with low or low-middle SDI to mitigate health inequality. Second, the discrepancy in the trend of ASR of ozone-attributable COPD death and DALY between men and women indicates that women may be more vulnerable to the deterioration of air quality and should receive more attention. Third, our panel analyses identified that smoking and high temperature may aggravate ozone-related COPD burden, whereas greenspace may alleviate the burden, providing an essential basis for identifying susceptible countries and formulating policy to mitigate the health effect of ozone pollution.

Our study has several limitations. First, in addition to COPD, other respiratory diseases such as asthma may also be associated with ozone pollution. Further studies are needed to assess the asthma burden attributable to ozone. Second, some potential modifiers (e.g., solar radiation, occupational exposures and indoor air pollution), were not included in panel analysis because of the lack of long-term continuous data at the national level. Third, as an ecological study, our estimates at the country-level could not fully rule out the potential for bias. Further studies about the modification of socioenvironmental factors on ozone-attributable COPD at the individual level are warranted. Fourth, there are a few ineluctable limitations in the GBD study. For instance, some countries lack relevant data or have data with low quality. To compensate for this, the GBD study used data processing and modelling methods to constantly improve the estimation [1].

### Conclusion

The ASR of ozone-attributable COPD death was increasing in regions with low or low-middle SDI, urging that more resources need to be invested in these regions to diminish ozone-related health inequalities between countries. Such increase was faster in women than man, which indicates women need more attention to reduce the respiratory effect of ozone. Increasing ambient temperature was associated with a higher burden of ozone-related COPD, and more green space may help alleviate the burden. These findings provide crucial basis for reducing the ozone-related COPD burden through urban design and coordinated control of ozone pollution and climate change.

## Data Availability

The data that support the findings of this study are openly available at the following URL/DOI: https://vizhub.healthdata.org/gbd-results/.

## References

[1] GBD Diseases Injuries Collaborators. Global Burden of 369 Diseases and Injuries in 204 Countries and Territories, 1990-2019: A Systematic Analysis for the Global Burden of Disease Study 2019. Lancet (2020) 396(10258):1204–22. 10.1016/S0140-6736(20)30925-9 33069326 PMC7567026

[2] Hansel NN McCormack MC Kim V. The Effects of Air Pollution and Temperature on COPD. COPD (2016) 13(3):372–9. 10.3109/15412555.2015.1089846 26683097 PMC4878829

[3] Huangfu P Atkinson R. Long-term Exposure to NO2 and O3 and All-Cause and Respiratory Mortality: A Systematic Review and Meta-Analysis. Environ Int (2020) 144:105998. 10.1016/j.envint.2020.105998 33032072 PMC7549128

[4] Gao H Wang K W WA Zhao W Xia ZL. A Systematic Review and Meta-Analysis of Short-Term Ambient Ozone Exposure and COPD Hospitalizations. Int J Environ Res Public Health (2020) 17(6):2130. 10.3390/ijerph17062130 32210080 PMC7143242

[5] GBD Chronic Respiratory Disease Collaborators. Prevalence and Attributable Health burden of Chronic Respiratory Diseases, 1990-2017: A Systematic Analysis for the Global Burden of Disease Study 2017. Lancet Respir Med (2020) 8(6):585–96. 10.1016/S2213-2600(20)30105-3 32526187 PMC7284317

[6] Li X Cao X Guo M Xie M Liu X. Trends and Risk Factors of Mortality and Disability Adjusted Life Years for Chronic Respiratory Diseases From 1990 to 2017: Systematic Analysis for the Global Burden of Disease Study 2017. BMJ (Clinical research ed) (2020) 368:m234. 10.1136/bmj.m234 PMC7190065 32075787

[7] Liu S Lim YH Pedersen M Jorgensen JT Amini H Cole-Hunter T Long-Term Air Pollution and Road Traffic Noise Exposure and COPD: The Danish Nurse Cohort. Eur Respir J (2021) 58(6):2004594. 10.1183/13993003.04594-2020 33986028

[8] Shin S Bai L Burnett RT Kwong JC Hystad P van Donkelaar A Air Pollution as a Risk Factor for Incident Chronic Obstructive Pulmonary Disease and Asthma. A 15-Year Population-Based Cohort Study. Am J Respir Crit Care Med (2021) 203(9):1138–48. 10.1164/rccm.201909-1744OC 33147059

[9] Wang C Xu J Yang L Xu Y Zhang X Bai C Prevalence and Risk Factors of Chronic Obstructive Pulmonary Disease in China (The China Pulmonary Health [CPH] Study): A National Cross-Sectional Study. Lancet (2018) 391(10131):1706–17. 10.1016/S0140-6736(18)30841-9 29650248

[10] Global Initiative for Chronic Obstructive Lung Disease. Global Strategy for the Diagnosis Management, and Prevention of Chronic Obstructive Pulmonary Disease (2022). Available at: https://goldcopd.org/wp-content/uploads/2021/12/GOLD-REPORT-2022-v1.1-22Nov2021_WMV.pdf (Accessed May 16, 2022).

[11] Atkinson RW Butland BK Dimitroulopoulou C Heal MR Stedman JR Carslaw N Long-term Exposure to Ambient Ozone and Mortality: A Quantitative Systematic Review and Meta-Analysis of Evidence From Cohort Studies. BMJ Open (2016) 6(2):e009493. 10.1136/bmjopen-2015-009493 PMC4769417 26908518

[12] Romanello M McGushin A Di Napoli C Drummond P Hughes N Jamart L The 2021 Report of the Lancet Countdown on Health and Climate Change: Code Red for a Healthy Future. Lancet (2021) 398(10311):1619–62. 10.1016/S0140-6736(21)01787-6 34687662 PMC7616807

[13] Bakola M Hernandez Carballo I Jelastopulu E Stuckler D. The Impact of COVID-19 Lockdown on Air Pollution in Europe and North America: A Systematic Review. Eur J Public Health (2022) 32(6):962–8. 10.1093/eurpub/ckac118 36074061 PMC9494388

[14] Wang T Xue L Brimblecombe P Lam YF Li L Zhang L. Ozone Pollution in China: A Review of Concentrations, Meteorological Influences, Chemical Precursors, and Effects. Sci total Environ (2017) 575:1582–96. 10.1016/j.scitotenv.2016.10.081 27789078

[15] Huang J Pan X Guo X Li G. Health Impact of China's Air Pollution Prevention and Control Action Plan: An Analysis of National Air Quality Monitoring and Mortality Data. Lancet Planet Health (2018) 2(7):e313–e23. 10.1016/S2542-5196(18)30141-4 30074894

[16] Yin P Brauer M Cohen AJ Wang H Li J Burnett RT The Effect of Air Pollution on Deaths, Disease Burden, and Life Expectancy across China and its Provinces, 1990-2017: An Analysis for the Global Burden of Disease Study 2017. Lancet Planet Health (2020) 4(9):e386–e98. 10.1016/S2542-5196(20)30161-3 32818429 PMC7487771

[17] Cai W Zhang C Suen HP Ai S Bai Y Bao J The 2020 China Report of the Lancet Countdown on Health and Climate Change. Lancet Public Health (2021) 6(1):e64–e81. 10.1016/S2468-2667(20)30256-5 33278345 PMC7966675

[18] Corradi M Alinovi R Goldoni M Vettori M Folesani G Mozzoni P Biomarkers of Oxidative Stress After Controlled Human Exposure to Ozone. Toxicol Lett (2002) 134(1-3):219–25. 10.1016/s0378-4274(02)00169-8 12191881

[19] Thurston GD Balmes JR Garcia E Gilliland FD Rice MB Schikowski T Outdoor Air Pollution and New-Onset Airway Disease. An Official American Thoracic Society Workshop Report. Ann Am Thorac Soc (2020) 17(4):387–98. 10.1513/AnnalsATS.202001-046ST 32233861 PMC7175976

[20] Srebot V Gianicolo EA Rainaldi G Trivella MG Sicari R. Ozone and Cardiovascular Injury. Cardiovasc Ultrasound (2009) 7:30. 10.1186/1476-7120-7-30 19552797 PMC2706799

[21] Li J Sun S Tang R Qiu H Huang Q Mason TG Major Air Pollutants and Risk of COPD Exacerbations: A Systematic Review and Meta-Analysis. Int J Chron Obstruct Pulmon Dis (2016) 11:3079–91. 10.2147/copd.s122282 28003742 PMC5161337

[22] Huang J Li G Xu G Qian X Zhao Y Pan X The Burden of Ozone Pollution on Years of Life Lost from Chronic Obstructive Pulmonary Disease in a City of Yangtze River Delta, China. Environ Pollut (2018) 242(Pt B):1266–73. 10.1016/j.envpol.2018.08.021 30121480

[23] Qiu H Tan K Long F Wang L Yu H Deng R The Burden of COPD Morbidity Attributable to the Interaction Between Ambient Air Pollution and Temperature in Chengdu, China. Int J Environ Res Public Health (2018) 15(3):492. 10.3390/ijerph15030492 29534476 PMC5877037

[24] Wang Y Wang K Cheng W Zhang Y. Global burden of Chronic Obstructive Pulmonary Disease Attributable to Ambient Ozone in 204 Countries and Territories During 1990-2019. Environ Sci Pollut Res Int (2022) 29(6):9293–305. 10.1007/s11356-021-16233-y 34505240

[25] Safiri S Carson-Chahhoud K Noori M Nejadghaderi SA Sullman MJM Ahmadian Heris J Burden of Chronic Obstructive Pulmonary Disease and its Attributable Risk Factors in 204 Countries and Territories, 1990-2019: Results From the Global Burden of Disease Study 2019. BMJ (Clinical research ed) (2022) 378:e069679. 10.1136/bmj-2021-069679 PMC9326843 35896191

[26] Zou J Sun T Song X Liu YM Lei F Chen MM Distributions and Trends of the Global Burden of COPD Attributable to Risk Factors by SDI, Age, and Sex From 1990 to 2019: A Systematic Analysis of GBD 2019 Data. Respir Res (2022) 23(1):90. 10.1186/s12931-022-02011-y 35410227 PMC8996417

[27] GBD Risk Factors Collaborators. Global burden of 87 Risk Factors in 204 Countries and Territories, 1990-2019: A Systematic Analysis for the Global Burden of Disease Study 2019. Lancet (2020) 396(10258):1223–49. 10.1016/S0140-6736(20)30752-2 33069327 PMC7566194

[28] Institute for Health Metrics and Evaluation. GBD Results (2019). Available from: https://vizhub.healthdata.org/gbd-results/ (Accessed June 15, 2022).

[29] Paulin LM Gassett AJ Alexis NE Kirwa K Kanner RE Peters S Association of Long-Term Ambient Ozone Exposure With Respiratory Morbidity in Smokers. JAMA Intern Med (2020) 180(1):106–15. 10.1001/jamainternmed.2019.5498 31816012 PMC6902160

[30] Gaffney AW Himmelstein DU Christiani DC Woolhandler S. Socioeconomic Inequality in Respiratory Health in the US From 1959 to 2018. JAMA Intern Med (2021) 181(7):968–76. 10.1001/jamainternmed.2021.2441 34047754 PMC8261605

[31] Fischer P Hoek G Brunekreef B Verhoeff A van Wijnen J. Air Pollution and Mortality in The Netherlands: Are the Elderly More at Risk? Eur Respir J Suppl (2003) 40:34s–8s. 10.1183/09031936.03.00402503 12762572

[32] Nordeide Kuiper I Svanes C Markevych I Accordini S Bertelsen RJ Braback L Lifelong Exposure to Air Pollution and Greenness in Relation to Asthma, Rhinitis and Lung Function in Adulthood. Environ Int (2021) 146:106219. 10.1016/j.envint.2020.106219 33126061

[33] Analitis A De' Donato F Scortichini M Lanki T Basagana X Ballester F Synergistic Effects of Ambient Temperature and Air Pollution on Health in Europe: Results From the PHASE Project. Int J Environ Res Public Health (2018) 15(9):1856. 10.3390/ijerph15091856 30154318 PMC6163671

[34] Institute for Health Metrics and Evaluation. Global Burden of Disease Study 2019 (GBD 2019) Data Resources (2019). Available from: https://ghdx.healthdata.org/gbd-2019 (Accessed June 15, 2022).

[35] Global Change Data Lab. Our World in Data (2021). Available from: https://ourworldindata.org/charts (Accessed June 15, 2022).

[36] Wyckoff MH Wyllie J Aziz K de Almeida MF Fabres JW Fawke J Neonatal Life Support 2020 International Consensus on Cardiopulmonary Resuscitation and Emergency Cardiovascular Care Science With Treatment Recommendations. Resuscitation (2020) 156:A156–A187. 10.1016/j.resuscitation.2020.09.015 33098917

[37] Mathieu E Ritchie H Ortiz-Ospina E Roser M Hasell J Appel C A Global Database of COVID-19 Vaccinations. Nat Hum Behav (2021) 5(7):947–53. 10.1038/s41562-021-01122-8 33972767

[38] National Oceanic and Atmospheric Administation. Assessing the Global Climate in June 2024 (2021). Available from: https://www.noaa.gov/ (Accessed June 15, 2022).

[39] Song J Pan R Yi W Wei Q Qin W Song S Ambient High Temperature Exposure and Global Disease Burden During 1990-2019: An Analysis of the Global Burden of Disease Study 2019. Sci total Environ (2021) 787:147540. 10.1016/j.scitotenv.2021.147540 33992940

[40] National Aeronautics and Space Administration. Moderate Resolution Imaging Spectroradiometer (2022). Available from: https://modis.gsfc.nasa.gov/ (Accessed June 15, 2022).

[41] Wang N Mengersen K Tong S Kimlin M Zhou M Hu W. Global, Regional, and National Burden of Lung Cancer and its Attributable Risk Factors, 1990 to 2017. Cancer (2020) 126(18):4220–34. 10.1002/cncr.33078 32648980

[42] Breto C Ionides EL King AA. Panel Data Analysis via Mechanistic Models. J Am Stat Assoc (2019) 115(531):1178–88. 10.1080/01621459.2019.1604367 32905476 PMC7472993

[43] Bollen KA Brand JE. A General Panel Model With Random and Fixed Effects: A Structural Equations Approach. Soc Forces (2010) 89(1):1–34. 10.1353/sof.2010.0072 21769157 PMC3137523

[44] Campelo Barroso Carneiro VC Ribeiro de Oliveira PT Rassy Carneiro S Cardoso Maciel M da Silva Pedroso J. Evidence of the Effect of Primary Care Expansion on Hospitalizations: Panel Analysis of 143 Municipalities in the Brazilian Amazon. PLoS One (2021) 16(4):e0248823. 10.1371/journal.pone.0248823 33831030 PMC8031449

[45] Cohen AJ Brauer M Burnett R Anderson HR Frostad J Estep K Estimates and 25-Year Trends of the Global Burden of Disease Attributable to Ambient Air Pollution: An Analysis of Data From the Global Burden of Diseases Study 2015. Lancet (2017) 389(10082):1907–18. 10.1016/S0140-6736(17)30505-6 28408086 PMC5439030

[46] Abdul Jabbar S Tul Qadar L Ghafoor S Rasheed L Sarfraz Z Sarfraz A Air Quality, Pollution and Sustainability Trends in South Asia: A Population-Based Study. Int J Environ Res Public Health (2022) 19(12):7534. 10.3390/ijerph19127534 35742785 PMC9224398

[47] Townend J Minelli C Mortimer K Obaseki DO Al Ghobain M Cherkaski H The Association Between Chronic Airflow Obstruction and Poverty in 12 Sites of the Multinational BOLD Study. Eur Respir J (2017) 49(6):1601880. 10.1183/13993003.01880-2016 28572124

[48] Gershon AS Warner L Cascagnette P Victor JC To T. Lifetime Risk of Developing Chronic Obstructive Pulmonary Disease: A Longitudinal Population Study. Lancet (2011) 378(9795):991–6. 10.1016/S0140-6736(11)60990-2 21907862

[49] Shin HH Gogna P Maquiling A Parajuli RP Haque L Burr B. Comparison of Hospitalization and Mortality Associated With Short-Term Exposure to Ambient Ozone and PM2.5 in Canada. Chemosphere (2021) 265:128683. 10.1016/j.chemosphere.2020.128683 33158503

[50] Clougherty JE. A Growing Role for Gender Analysis in Air Pollution Epidemiology. Cien Saude Colet (2011) 16(4):2221–38. 10.1590/s1413-81232011000400021 21584463

[51] World Health Organization. WHO Report on the Global Tobacco Epidemic 2021: Addressing New and Emerging Products (2021). Available from: https://www.who.int/publications/i/item/9789240032095 (Accessed July 27, 2021).

[52] Xing DF Xu CD Liao XY Xing TY Cheng SP Hu MG Spatial Association Between Outdoor Air Pollution and Lung Cancer Incidence in China. BMC Public Health (2019) 19(1):1377. 10.1186/s12889-019-7740-y 31655581 PMC6815434

[53] GBD Tobacco Collaborators. Spatial, Temporal, and Demographic Patterns in Prevalence of Smoking Tobacco Use and Attributable Disease Burden in 204 Countries and Territories, 1990-2019: A Systematic Analysis From the Global Burden of Disease Study 2019. Lancet (2021) 397(10292):2337–60. 10.1016/S0140-6736(21)01169-7 34051883 PMC8223261

[54] Ren C Williams GM Mengersen K Morawska L Tong S. Does Temperature Modify Short-Term Effects of Ozone on Total Mortality in 60 Large Eastern US Communities? An Assessment Using the NMMAPS Data. Environ Int (2008) 34(4):451–8. 10.1016/j.envint.2007.10.001 17997483

[55] Crouse DL Pinault L Balram A Hystad P Peters PA Chen H Urban Greenness and Mortality in Canada's Largest Cities: A National Cohort Study. Lancet Planet Health (2017) 1(7):e289–e97. 10.1016/S2542-5196(17)30118-3 29851627

[56] Bereziartua A Chen J de Hoogh K Rodopoulou S Andersen ZJ Bellander T Exposure to Surrounding Greenness and Natural-Cause and Cause-Specific Mortality in the ELAPSE Pooled Cohort. Environ Int (2022) 166:107341. 10.1016/j.envint.2022.107341 35717714

[57] Stas M Aerts R Hendrickx M Delcloo A Dendoncker N Dujardin S Exposure to Green Space and Pollen Allergy Symptom Severity: A Case-Crossover Study in Belgium. Sci total Environ (2021) 781:146682. 10.1016/j.scitotenv.2021.146682 33812114

[58] Heo S Bell ML. The Influence of Green Space on the Short-Term Effects of Particulate Matter on Hospitalization in the U.S. For 2000-2013. Environ Res (2019) 174:61–8. 10.1016/j.envres.2019.04.019 31039514 PMC6550459

[59] Sun S Sarkar C Kumari S James P Cao W Lee RS Air Pollution Associated Respiratory Mortality Risk Alleviated by Residential Greenness in the Chinese Elderly Health Service Cohort. Environ Res (2020) 183:109139. 10.1016/j.envres.2020.109139 31999997 PMC9847333

[60] Kasdagli MI Katsouyanni K de Hoogh K Lagiou P Samoli E. Associations of Air Pollution and Greenness With Mortality in Greece: An Ecological Study. Environ Res (2021) 196:110348. 10.1016/j.envres.2020.110348 33127394

[61] Markevych I Schoierer J Hartig T Chudnovsky A Hystad P Dzhambov AM Exploring Pathways Linking Greenspace to Health: Theoretical and Methodological Guidance. Environ Res (2017) 158:301–17. 10.1016/j.envres.2017.06.028 28672128

[62] James P Hart JE Banay RF Laden F. Exposure to Greenness and Mortality in a Nationwide Prospective Cohort Study of Women. Environ Health Perspect (2016) 124(9):1344–52. 10.1289/ehp.1510363 27074702 PMC5010419

