# Supplementary material for: Potential Drivers for the Spatiotemporal Patterns of the Global Burden of Chronic Obstructive Pulmonary Disease Attributable to Ambient Ozone, 1990–2019
Source: Int J Public Health. 2024 Jul 23;69:1606062. doi: 10.3389/ijph.2024.1606062 (PMC11300243; doi:10.3389/ijph.2024.1606062)
Supplement: Supplementary file 1 [file DataSheet1.docx]

**Supplementary materials**

[Figure S1. The proportions of COPD DALYs attrttibutable to ozone among total COPD DALYs by sex and region, 1990 and 2019 3](#_Toc120715341)

[Figure S2. The change in the proportions of COPD DALYs attributable to ozone among total COPD DALYs by sex and country, 1990 to 2019 4](#_Toc120715342)

[Figure S3. The age-standardized DALY rates of COPD attributable to ozone by sex and country, 2019 5](#_Toc120715343)

[Figure S4. Annual percentage change in the age-standardized DALY rates of COPD attributable to ozone by sex and country, 1990 to 2019 6](#_Toc120715344)

[Table S1. DALY numbers and age-standardized DALY rates of COPD attributable to ozone, 1990 and 2019 7](#_Toc120715345)

[Table S2. Number, percentage, and age-standardized rate of deaths from COPD attributable to ozone by country, 1990 and 2019 11](#_Toc120715346)

[Table S3. Number, percentage, and age-standardized rate of DALYs from COPD attributable to ozone pollution by country, 1990-2019 34](#_Toc120715347)

[Table S4. Association between country-level factors and DALY rate of COPD attributable to ozone pollution 58](#_Toc120715348)

# Figure S1. The proportions of COPD DALYs attributable to ozone among total COPD DALYs by sex and region, 1990 and 2019


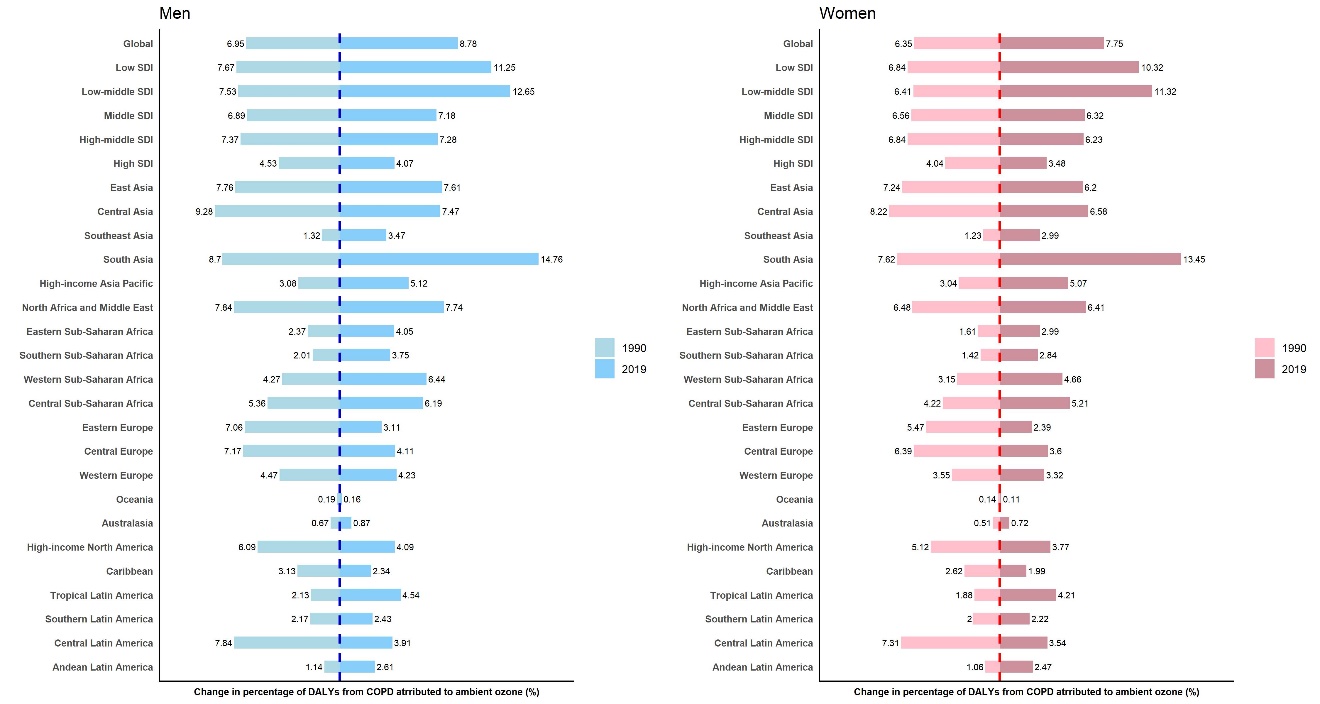


# Figure S2. The change in the proportions of COPD DALYs attributable to ozone among total COPD DALYs by sex and country, 1990 to 2019

# (A) Men


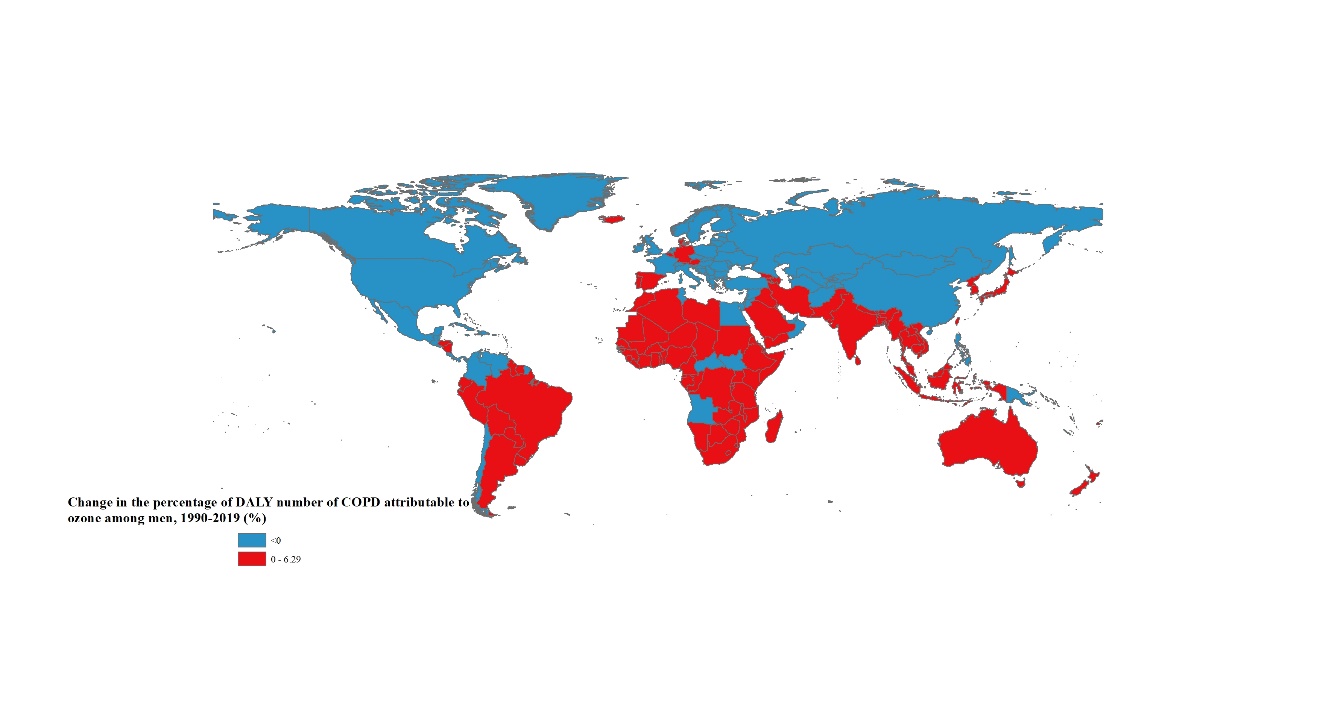


# (B) Women


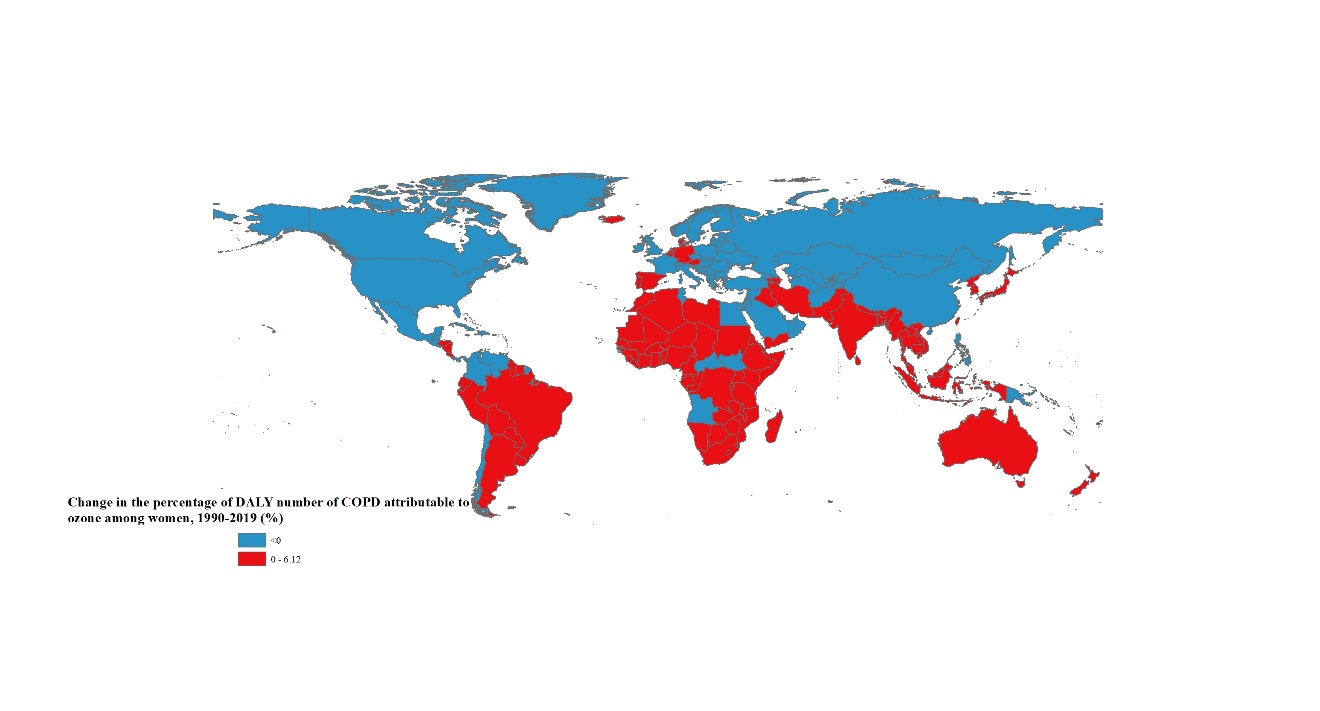


# Figure S3. The age-standardized DALY rates of COPD attributable to ozone by sex and country, 2019

# (A) Men


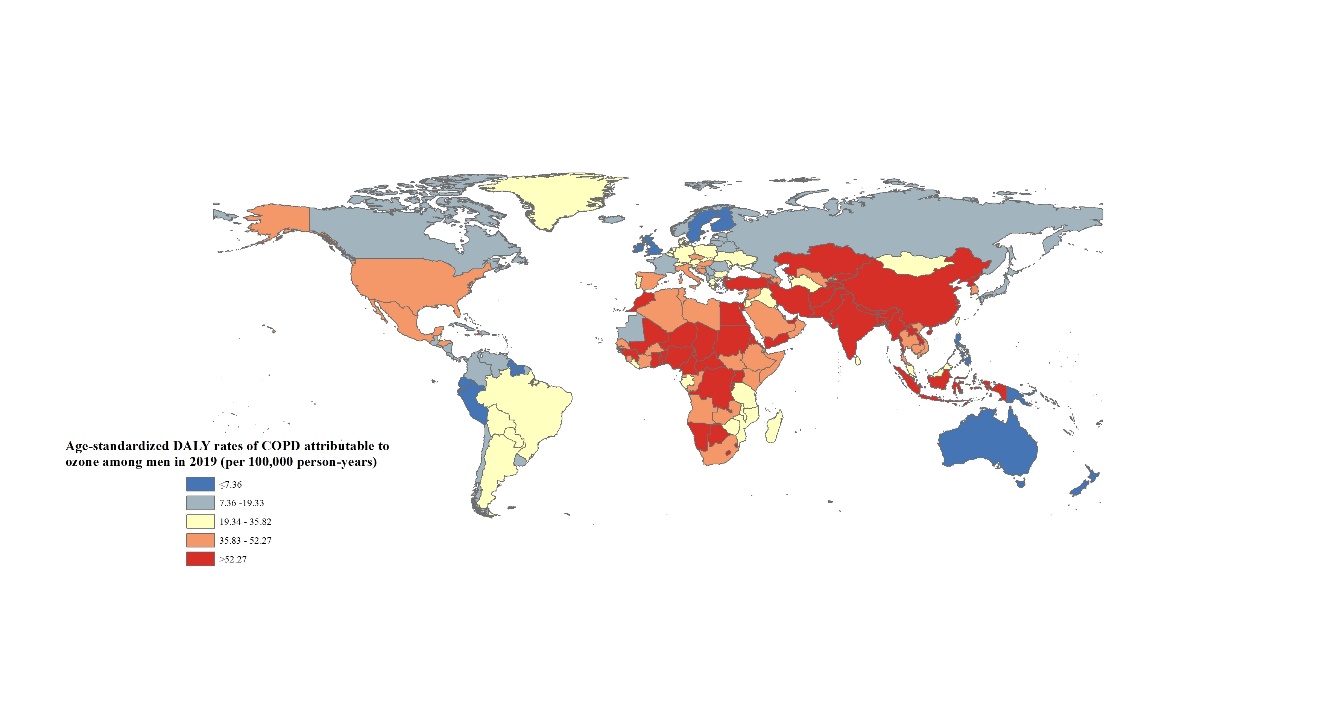


# (B) Women


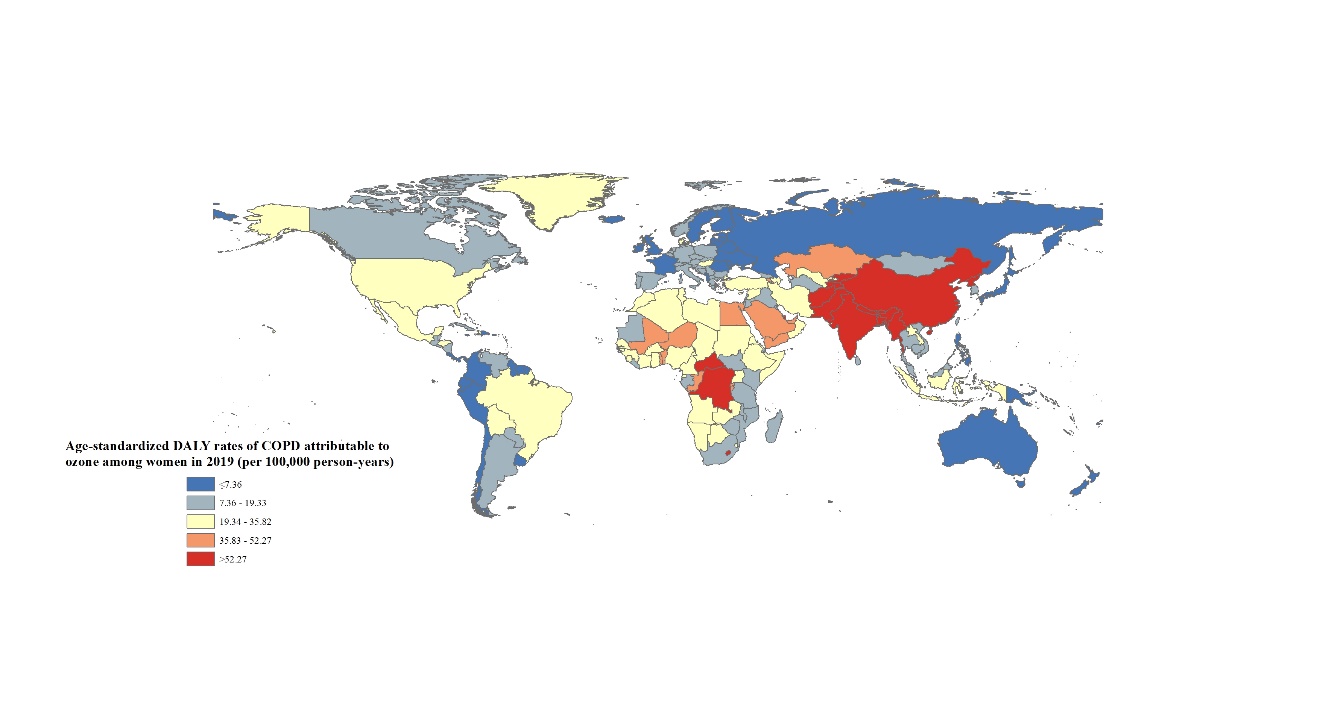


# Figure S4. Annual percentage change in the age-standardized DALY rates of COPD attributable to ozone by sex and country, 1990 to 2019

# (A) Men


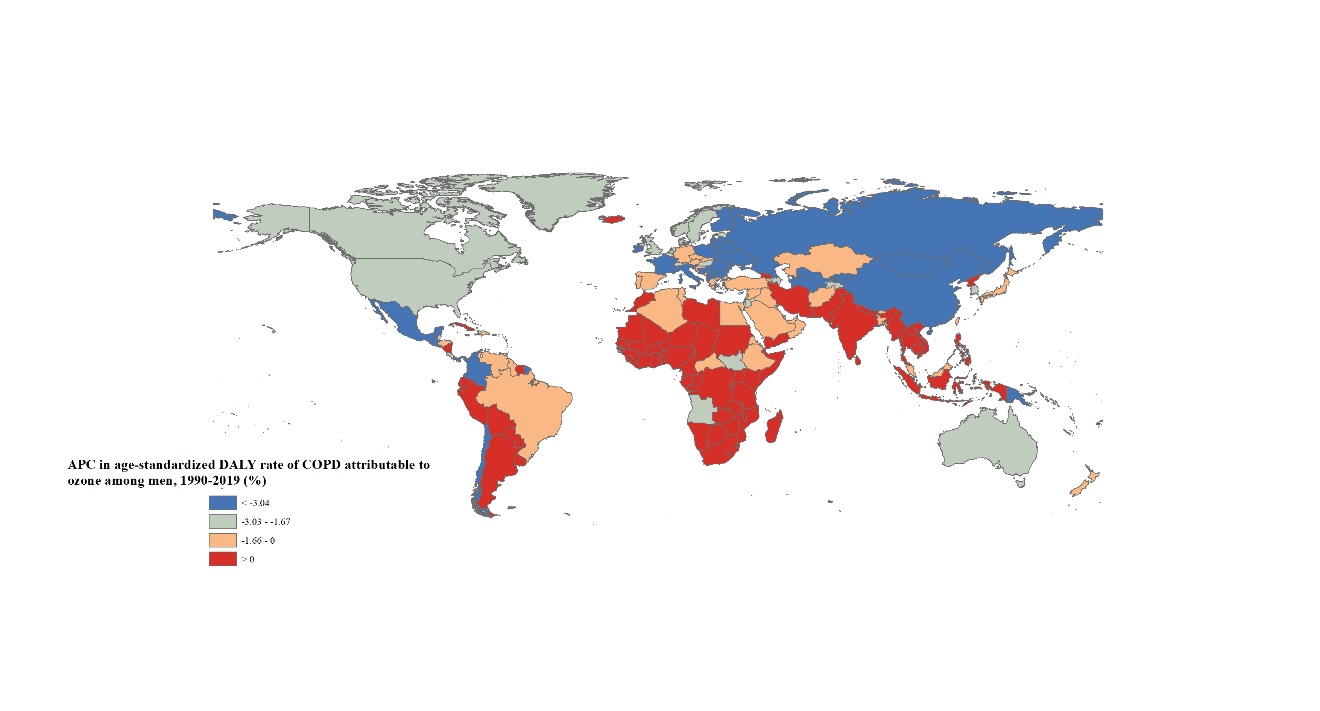


# (B) Women


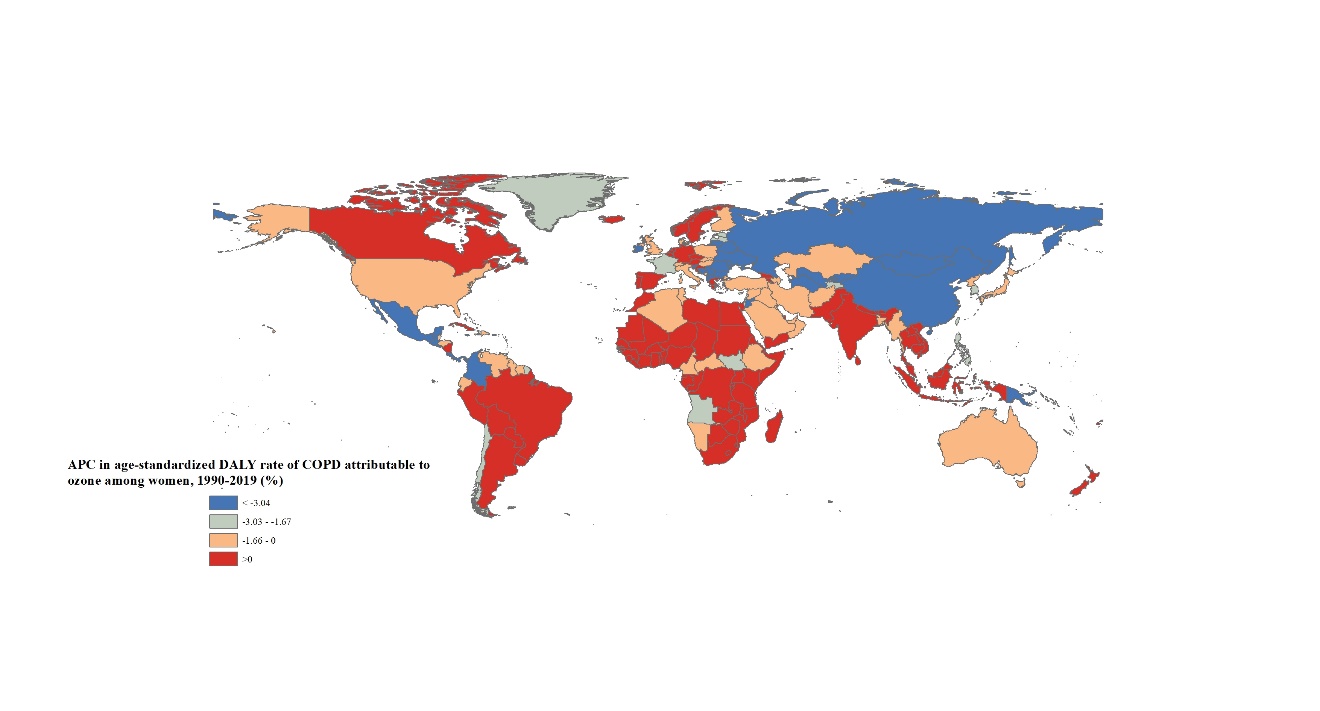


# Table S1. DALY numbers and age-standardized DALY rates of COPD attributable to ozone, 1990 and 2019

(A) Men

| Regions | DALY numbers (Uncertainty Intervals) | |  | Age-standardized DALY rates (Uncertainty intervals) | | |  |
| --- | --- | --- | --- | --- | --- | --- | --- |
|  | 1990 | 2019 |  | 1990 ^a^ | 2019 ^a^ | Annual percentage change |  |
| Global | 2351263 (1045805, 3796271) | 3695020 (1721583, 5805269) |  | 139.64 (62.05, 224.51) | 100.82 (46.97, 158.02) | -1.3% (-1.54, -1.06) |  |
| Socio-demographic index | | | | | | |  |
| Low | 174698 (76779, 283002) | 418124 (191989, 651964) |  | 164.08 (72.77, 266.18) | 191.62 (86.93, 296.3) | 0.53% (0.34, 0.71) |  |
| Low-middle | 631307 (275349, 1007539) | 1585905 (743825, 2507917) |  | 231.96 (100.27, 370.59) | 264.69 (124.23, 417.32) | 0.4% (0.25, 0.54) |  |
| Middle | 773156 (336723, 1263086) | 952569 (440134, 1510213) |  | 179.97 (79.83, 293.66) | 91.24 (41.94, 144.65) | -2.34% (-2.85, -1.83) |  |
| High-middle | 600243 (266461, 969175) | 515036 (241996, 802037) |  | 145.76 (64.75, 235.14) | 61.3 (28.64, 95.65) | -3.43% (-3.77, -3.1) |  |
| High | 171626 (77357, 279605) | 222873 (98101, 366047) |  | 39.88 (17.99, 65.12) | 25.74 (11.29, 42.25) | -1.7% (-1.95, -1.45) |  |
| GBD region |  |  |  |  |  |  |  |
| East Asia | 1079373 (476463, 1771280) | 870868 (383953, 1399193) |  | 325.43 (141.57, 530.06) | 106.99 (47.78, 171.18) | -3.69% (-4.35, -3.04) |  |
| Central Asia | 22021 (10067, 34409) | 19732 (9046, 31338) |  | 121.22 (55.27, 189.01) | 71.29 (32.93, 112.34) | -2.41% (-2.81, -2) |  |
| Southeast Asia | 25321 (8508, 50077) | 117015 (50686, 200631) |  | 23.56 (7.91, 46.48) | 47.99 (21.08, 81.95) | 2.31% (1.6, 3.04) |  |
| South Asia | 742217 (323171, 1173099) | 2089362 (990221, 3269260) |  | 289.19 (128.17, 460.14) | 332.35 (158.63, 518.78) | 0.38% (0.2, 0.56) |  |
| High-income Asia Pacific | 13132 (5452, 22374) | 37140 (16217, 60961) |  | 17.24 (7.19, 29.53) | 17.04 (7.48, 27.84) | -0.67% (-1.02, -0.31) |  |
| North Africa and Middle East | 61595 (28284, 97311) | 130708 (60287, 207492) |  | 76.61 (34.98, 121.2) | 63.43 (29.16, 100.1) | -0.41% (-0.58, -0.24) |  |
| Eastern Sub-Saharan Africa | 11029 (4267, 20724) | 28822 (12203, 49369) |  | 31.54 (12.13, 58.22) | 42.04 (18.08, 71.61) | 1.08% (-0.17, 2.34) |  |
| Southern Sub-Saharan Africa | 3153 (1190, 5730) | 9651 (4207, 15977) |  | 28.19 (10.65, 51.57) | 44.68 (19.47, 74.37) | 1.72% (0.85, 2.59) |  |
| Western Sub-Saharan Africa | 16490 (6694, 29002) | 44850 (19253, 74714) |  | 41.66 (17.11, 72.93) | 55.23 (23.4, 91.31) | 1.35% (0.39, 2.32) |  |
| Central Sub-Saharan Africa | 7507 (3021, 13259) | 15576 (6190, 27743) |  | 77.58 (31.27, 134.55) | 74.1 (29.86, 130.45) | 0.16% (-0.98, 1.31) |  |
| Eastern Europe | 94737 (39980, 152702) | 25709 (9871, 44892) |  | 101.36 (42.89, 163.21) | 20.24 (7.84, 35.26) | -6.26% (-6.78, -5.73) |  |
| Central Europe | 48392 (21888, 76686) | 23923 (10453, 39974) |  | 79.61 (35.94, 126.27) | 26.33 (11.49, 44.06) | -3.73% (-4.07, -3.39) |  |
| Western Europe | 93059 (41674, 153478) | 100613 (44948, 164587) |  | 40.06 (17.95, 66.32) | 23.69 (10.57, 38.98) | -1.97% (-2.23, -1.7) |  |
| Oceania | 84 (21, 205) | 133 (44, 271) |  | 6.44 (1.57, 15.53) | 4.32 (1.39, 8.89) | -2.45% (-3.29, -1.61) |  |
| Australasia | 702 (216, 1448) | 1123 (372, 2227) |  | 6.95 (2.13, 14.38) | 4.71 (1.56, 9.35) | -1.97% (-2.76, -1.18) |  |
| High-income North America | 93678 (43048, 147217) | 106947 (46370, 177897) |  | 62.33 (28.63, 97.87) | 36.96 (16.04, 61.43) | -1.94% (-2.36, -1.52) |  |
| Caribbean | 1991 (794, 3628) | 3152 (1155, 5916) |  | 16.26 (6.5, 29.48) | 13.13 (4.81, 24.65) | -1.44% (-2.15, -0.73) |  |
| Tropical Latin America | 10659 (3963, 19474) | 35961 (15045, 60978) |  | 27.18 (10.13, 49.75) | 34.66 (14.54, 58.52) | -0.44% (-1.24, 0.37) |  |
| Southern Latin America | 3688 (1381, 6856) | 6378 (2490, 11568) |  | 18.68 (7.01, 34.77) | 17.79 (6.94, 32.18) | -0.43% (-0.72, -0.15) |  |
| Central Latin America | 21849 (10652, 32780) | 25199 (10647, 43222) |  | 60.93 (29.73, 91.74) | 24.47 (10.32, 42.02) | -3.81% (-4.1, -3.52) |  |
| Andean Latin America | 587 (214, 1150) | 2158 (850, 3955) |  | 6.34 (2.32, 12.35) | 8.47 (3.33, 15.57) | 0.97% (-0.14, 2.09) |  |

^a^ per 100,000 person-years

(B) Women

| Regions | DALY numbers (Uncertainty intervals) | |  | Age-standardized DALY rates (Uncertainty intervals) | | |
| --- | --- | --- | --- | --- | --- | --- |
|  | 1990 | 2019 |  | 1990 ^a^ | 2019 ^a^ | Annual percentage change |
| Global | 1620207 (729174, 2638684) | 2515125 (1181391, 3964167) |  | 77.68 (34.92, 126.30) | 57.35 (26.95, 90.39) | -1.36% (-1.58, -1.13) |
| Socio-demographic index | | | | | | |
| Low | 107569 (44519, 183762) | 311483 (148305, 504530) |  | 102.45 (42.55, 174.71) | 136 (64.58, 218.37) | 1.3% (1.11, 1.49) |
| Low-middle | 359894 (156275, 603200) | 1098684 (508336, 1767626) |  | 136.38 (59.37, 226.94) | 166.66 (77.02, 267.51) | 0.71% (0.52, 0.9) |
| Middle | 619081 (268801, 1027311) | 610096 (273741, 989818) |  | 132.23 (58.93, 218.45) | 51.09 (23.05, 83.03) | -3.05% (-3.6, -2.49) |
| High-middle | 423186 (183711, 704239) | 325944 (147450, 538633) |  | 71.7 (31.02, 119.23) | 27.82 (12.58, 45.86) | -3.38% (-3.75, -3) |
| High | 110348 (49217, 180648) | 168635 (72513, 285214) |  | 17.73 (7.92, 29) | 15.59 (6.8, 26.06) | -0.47% (-0.91, -0.04) |
| GBD region |  |  |  |  |  |  |
| East Asia | 923886 (394795, 1554336) | 574446 (259679, 982524) |  | 236.07 (102.51, 397.92) | 56.53 (25.61, 96.65) | -4.99% (-5.68, -4.3) |
| Central Asia | 14495 (6486, 23343) | 12295 (5762, 20041) |  | 53.03 (23.77, 85.26) | 33.09 (15.45, 53.79) | -2.2% (-2.59, -1.81) |
| Southeast Asia | 14750 (4864, 29501) | 53024 (22366, 90598) |  | 11.81 (3.95, 23.68) | 17.71 (7.48, 30.3) | 1.12% (0.43, 1.82) |
| South Asia | 401190 (168254, 689537) | 1479461 (682286, 2373101) |  | 176.19 (75.51, 300) | 227.85 (106.19, 364.03) | 0.63% (0.39, 0.86) |
| High-income Asia Pacific | 6840 (2788, 11768) | 17571 (7554, 29590) |  | 6.11 (2.5, 10.49) | 5.58 (2.41, 9.41) | -0.99% (-1.28, -0.69) |
| North Africa and Middle East | 34450 (15205, 58050) | 68957 (30662, 108371) |  | 43.7 (19.27, 73.46) | 35.39 (16.27, 55.94) | -0.56% (-0.73, -0.39) |
| Eastern Sub-Saharan Africa | 5175 (1695, 10368) | 15739 (6285, 29199) |  | 14.12 (4.85, 28.01) | 20.07 (8.22, 36.48) | 1.31% (-0.01, 2.64) |
| Southern Sub-Saharan Africa | 1788 (677, 3455) | 5885 (2529, 9960) |  | 11.9 (4.52, 22.96) | 19.1 (8.21, 32.49) | 2.12% (1.27, 2.99) |
| Western Sub-Saharan Africa | 9509 (4016, 16272) | 26355 (11853, 44563) |  | 22.67 (9.54, 38.8) | 28.84 (12.97, 48.35) | 1.14% (0.15, 2.13) |
| Central Sub-Saharan Africa | 5236 (1826, 10961) | 13671 (5001, 30274) |  | 49.82 (17.49, 103.19) | 54.17 (19.82, 123.08) | 0.75% (-0.41, 1.92) |
| Eastern Europe | 43196 (15516, 73354) | 10657 (4047, 20233) |  | 23.61 (8.51, 40.09) | 4.77 (1.84, 9.05) | -5.92% (-6.2, -5.64) |
| Central Europe | 20585 (9107, 33228) | 12907 (5495, 21459) |  | 24.55 (10.85, 39.59) | 10.14 (4.32, 17) | -2.87% (-3.21, -2.52) |
| Western Europe | 43678 (19031, 73561) | 63961 (27345, 108173) |  | 11.91 (5.19, 19.99) | 10.97 (4.76, 18.64) | -0.25% (-0.52, 0.02) |
| Oceania | 49 (11, 121) | 80 (25, 173) |  | 3.67 (0.83, 9.19) | 2.58 (0.8, 5.57) | -2.29% (-3.29, -1.28) |
| Australasia | 348 (105, 718) | 921 (300, 1890) |  | 2.61 (0.78, 5.42) | 3.3 (1.07, 6.72) | 0.2% (-0.45, 0.86) |
| High-income North America | 68873 (31184, 110872) | 102974 (40361, 175667) |  | 33.57 (15.1, 53.81) | 29.23 (11.59, 49.9) | -0.61% (-1.2, -0.01) |
| Caribbean | 1202 (472, 2376) | 2068 (707, 4085) |  | 8.99 (3.52, 17.78) | 7.52 (2.57, 14.82) | -1.27% (-1.97, -0.58) |
| Tropical Latin America | 6354 (2296, 11775) | 27893 (11788, 47953) |  | 13.96 (5.02, 26.04) | 21.05 (8.89, 36.21) | 0.2% (-0.65, 1.06) |
| Southern Latin America | 1717 (632, 3237) | 4235 (1580, 7782) |  | 6.82 (2.51, 12.87) | 8.66 (3.28, 15.75) | 0.65% (0.39, 0.92) |
| Central Latin America | 16411 (7794, 25054) | 20209 (8196, 34503) |  | 42.13 (19.93, 64.47) | 16.28 (6.57, 27.8) | -3.84% (-4.15, -3.54) |
| Andean Latin America | 473 (165, 915) | 1817 (741, 3354) |  | 4.76 (1.66, 9.34) | 6.38 (2.61, 11.77) | 0.98% (-0.19, 2.16) |

^a^ per 100,000 person-years

# Table S2. Number, percentage, and age-standardized rate of deaths from COPD attributable to ozone by country, 1990 and 2019

(A) Men

| Country | Percentage (%) | |  | Death number | |  | Age-standardized mortality rate | | |
| --- | --- | --- | --- | --- | --- | --- | --- | --- | --- |
|  | 1990  (uncertainty interval) | 2019  (uncertainty interval) |  | 1990  (uncertainty interval) | 2019  (uncertainty interval) |  | 1990  (uncertainty interval)  (per 100,000 person-years) | 2019  (uncertainty interval)  (per 100,000 person-years) | Annual percentage change, 1990-2019 (confidence interval) (%) |
| Afghanistan | 0.14 (0.06, 0.2) | 0.12 (0.06, 0.19) |  | 234 (90, 418) | 242 (102, 411) |  | 7.54 (2.89,13.36) | 5.36 (2.24, 9.02) | -1.03 (-1.34, -0.72) |
| Albania | 0.11 (0.05, 0.17) | 0.04 (0.02, 0.07) |  | 52 (23, 82) | 16 (6, 28) |  | 8.07 (3.59,12.83) | 0.86 (0.32, 1.6) | -7 (-7.42, -6.58) |
| Algeria | 0.1 (0.05, 0.16) | 0.13 (0.06, 0.19) |  | 137 (56, 234) | 377 (170, 639) |  | 3.61 (1.51,6.15) | 2.94 (1.31, 4.93) | -1.03 (-1.35, -0.72) |
| American Samoa | 0.02 (0.01, 0.04) | 0.02 (0.01, 0.04) |  | 0 (0, 0) | 0 (0, 0) |  | 1.94 (0.5,4.28) | 1.2 (0.31, 2.55) | -3.26 (-3.96, -2.55) |
| Andorra | 0.07 (0.03, 0.13) | 0.09 (0.04, 0.15) |  | 1 (0, 1) | 2 (1, 4) |  | 3.78 (1.37,7.11) | 3.05 (1.21, 5.25) | -0.64 (-0.99, -0.28) |
| Angola | 0.1 (0.04, 0.15) | 0.07 (0.03, 0.11) |  | 72 (31, 123) | 75 (32, 128) |  | 5.42 (2.35,9.07) | 2.36 (0.98, 3.96) | -2.4 (-3.11, -1.69) |
| Antigua and Barbuda | 0.03 (0, 0.06) | 0 (0, 0) |  | 0 (0, 0) | 0 (0, 0) |  | 0.35 (0.06,0.85) | 0 (0, 0.02) | -18.35 (-20.86, -15.76) |
| Argentina | 0.03 (0.01, 0.05) | 0.03 (0.01, 0.06) |  | 134 (49, 254) | 297 (111, 539) |  | 1.09 (0.4,2.08) | 1.36 (0.51, 2.47) | 0.57 (0.25, 0.9) |
| Armenia | 0.1 (0.04, 0.16) | 0.13 (0.06, 0.19) |  | 60 (27, 99) | 83 (36, 134) |  | 6.85 (3.04,11.32) | 5.32 (2.28, 8.62) | -1.09 (-1.46, -0.72) |
| Australia | 0.01 (0, 0.02) | 0.01 (0, 0.02) |  | 36 (11, 75) | 64 (21, 128) |  | 0.48 (0.15,1) | 0.31 (0.1, 0.63) | -2.03 (-2.87, -1.18) |
| Austria | 0.08 (0.04, 0.13) | 0.09 (0.04, 0.14) |  | 98 (46, 158) | 158 (72, 253) |  | 2.4 (1.1,3.85) | 1.94 (0.88, 3.11) | -1.05 (-1.5, -0.61) |
| Azerbaijan | 0.08 (0.04, 0.13) | 0.1 (0.04, 0.15) |  | 63 (28, 103) | 84 (35, 144) |  | 3.84 (1.7,6.19) | 3.14 (1.3, 5.33) | -1.35 (-2.33, -0.35) |
| Bahamas | 0.04 (0.01, 0.08) | 0.03 (0.01, 0.07) |  | 0 (0, 1) | 1 (0, 2) |  | 0.69 (0.17,1.48) | 0.54 (0.12, 1.26) | -1.44 (-2.19, -0.69) |
| Bahrain | 0.13 (0.05, 0.22) | 0.17 (0.08, 0.29) |  | 5 (2, 8) | 12 (5, 20) |  | 10 (3.5,17.44) | 5.84 (2.61, 9.87) | -2.06 (-2.7, -1.41) |
| Bangladesh | 0.09 (0.04, 0.14) | 0.18 (0.09, 0.26) |  | 2773 (1178, 4646) | 6530 (3047, 11406) | | 12.53 (5.29,20.92) | 10.93 (5.02, 19.21) | -0.93 (-1.31, -0.56) |
| Barbados | 0.02 (0, 0.05) | 0 (0, 0) |  | 0 (0, 1) | 0 (0, 0) |  | 0.24 (0.03,0.6) | 0 (0, 0.04) | -15.62 (-17.47, -13.73) |
| Belarus | 0.06 (0.02, 0.09) | 0.04 (0.01, 0.06) |  | 182 (70, 313) | 50 (19, 96) |  | 4.6 (1.78,7.91) | 0.95 (0.37, 1.83) | -6.7 (-7.36, -6.04) |
| Belgium | 0.04 (0.01, 0.07) | 0.05 (0.02, 0.08) |  | 134 (49, 243) | 186 (75, 334) |  | 2.36 (0.86,4.26) | 1.72 (0.69, 3.1) | -1.89 (-2.66, -1.12) |
| Belize | 0.05 (0.02, 0.09) | 0.04 (0.02, 0.08) |  | 0 (0, 1) | 2 (1, 3) |  | 1.17 (0.45,2.07) | 1.27 (0.47, 2.47) | -0.63 (-1.63, 0.39) |
| Benin | 0.06 (0.02, 0.1) | 0.11 (0.05, 0.18) |  | 24 (10, 45) | 70 (30, 116) |  | 2.99 (1.23,5.52) | 4.26 (1.82, 7.22) | 1.58 (0.49, 2.68) |
| Bermuda | 0.15 (0.07, 0.23) | 0.08 (0.04, 0.14) |  | 1 (0, 1) | 1 (0, 1) |  | 3.08 (1.44,4.86) | 1.24 (0.52, 2.15) | -3.07 (-3.37, -2.76) |
| Bhutan | 0.1 (0.05, 0.15) | 0.14 (0.07, 0.21) |  | 10 (4, 19) | 33 (14, 62) |  | 13.13 (5.27,23.79) | 14.04 (6.25, 26.08) | 0.35 (0.12, 0.59) |
| Bolivia (Plurinational State of) | 0.03 (0.01, 0.05) | 0.05 (0.02, 0.09) |  | 17 (6, 33) | 64 (25, 115) |  | 1.64 (0.62,3.1) | 2.08 (0.82, 3.75) | 1.15 (-0.43, 2.76) |
| Bosnia and Herzegovina | 0.1 (0.05, 0.16) | 0.08 (0.04, 0.13) |  | 60 (27, 94) | 55 (24, 92) |  | 4.55 (2.05,7.16) | 2.33 (1.01, 3.92) | -2.82 (-3.26, -2.38) |
| Botswana | 0.04 (0.02, 0.07) | 0.06 (0.02, 0.1) |  | 6 (2, 13) | 15 (6, 28) |  | 3.4 (1.2,6.54) | 3.76 (1.42, 6.82) | 0.64 (-0.22, 1.5) |
| Brazil | 0.02 (0.01, 0.04) | 0.05 (0.02, 0.09) |  | 517 (192, 947) | 2000 (833, 3400) | | 1.63 (0.6,2.99) | 2.15 (0.9, 3.67) | -0.29 (-1.1, 0.52) |
| Brunei Darussalam | 0 (0, 0.01) | 0.01 (0, 0.03) |  | 0 (0, 0) | 0 (0, 1) |  | 0.59 (0.04,1.89) | 0.77 (0.04, 2.57) | -0.72 (-2.57, 1.17) |
| Bulgaria | 0.1 (0.05, 0.16) | 0.05 (0.02, 0.09) |  | 227 (102, 359) | 94 (39, 162) |  | 4.69 (2.13,7.42) | 1.6 (0.65, 2.74) | -3.35 (-4.44, -2.25) |
| Burkina Faso | 0.05 (0.02, 0.08) | 0.09 (0.04, 0.14) |  | 22 (9, 42) | 69 (30, 116) |  | 1.47 (0.58,2.73) | 2.33 (1.01, 3.89) | 1.73 (0.5, 2.98) |
| Burundi | 0.03 (0.01, 0.06) | 0.08 (0.04, 0.13) |  | 24 (9, 48) | 77 (29, 144) |  | 2.78 (1,5.34) | 4.64 (1.83, 8.63) | 2.22 (0.74, 3.72) |
| Cabo Verde | 0.02 (0, 0.08) | 0.05 (0, 0.12) |  | 1 (0, 4) | 2 (0, 5) |  | 1.15 (0,4.05) | 1.16 (0.06, 3.08) | -1.04 (-2.54, 0.48) |
| Cambodia | 0.01 (0, 0.03) | 0.05 (0.02, 0.08) |  | 13 (4, 28) | 101 (42, 179) |  | 0.93 (0.29,2.01) | 2.93 (1.18, 5.2) | 4.91 (3.76, 6.08) |
| Cameroon | 0.07 (0.03, 0.11) | 0.1 (0.05, 0.16) |  | 57 (23, 101) | 147 (59, 252) |  | 3.63 (1.52,6.38) | 3.6 (1.52, 6.04) | 0.16 (-1.17, 1.5) |
| Canada | 0.04 (0.02, 0.07) | 0.04 (0.02, 0.07) |  | 207 (81, 359) | 331 (126, 589) |  | 1.66 (0.65,2.89) | 1.03 (0.39, 1.83) | -2 (-2.56, -1.43) |
| Central African Republic | 0.13 (0.06, 0.19) | 0.12 (0.05, 0.19) |  | 40 (17, 71) | 55 (22, 99) |  | 10.32 (4.13,18) | 8.31 (3.44, 14.49) | -0.93 (-1.88, 0.03) |
| Chad | 0.07 (0.03, 0.11) | 0.09 (0.04, 0.14) |  | 38 (15, 74) | 81 (32, 139) |  | 3.31 (1.3,6.32) | 3.5 (1.44, 5.92) | 0.83 (-0.1, 1.78) |
| Chile | 0.04 (0.02, 0.07) | 0.03 (0.01, 0.05) |  | 47 (18, 82) | 67 (23, 130) |  | 1.25 (0.49,2.18) | 0.68 (0.24, 1.32) | -2.32 (-2.91, -1.72) |
| China | 0.09 (0.04, 0.14) | 0.09 (0.04, 0.14) |  | 54083 (23730, 88279) | 53427 (23653, 85491) | | 22.76 (10.05,36.71) | 8.52 (3.82, 13.54) | -3.18 (-3.86, -2.51) |
| Colombia | 0.02 (0.01, 0.03) | 0.02 (0.01, 0.03) |  | 53 (18, 99) | 140 (45, 279) |  | 0.77 (0.27,1.45) | 0.58 (0.19, 1.15) | -2.75 (-3.7, -1.79) |
| Comoros | 0 (0, 0.02) | 0.02 (0, 0.07) |  | 0 (0, 1) | 1 (0, 5) |  | 0.14 (0,1.52) | 0.68 (0, 2.81) | 5.07 (2.32, 7.88) |
| Congo | 0.02 (0.01, 0.05) | 0.08 (0.03, 0.13) |  | 6 (2, 13) | 26 (9, 47) |  | 1.79 (0.56,3.72) | 3.11 (1.09, 5.58) | 2.64 (0.59, 4.74) |
| Cook Islands | 0.02 (0, 0.07) | 0.02 (0.01, 0.04) |  | 0 (0, 0) | 0 (0, 0) |  | 1.21 (0,4.39) | 0.57 (0.15, 1.22) | -4.26 (-4.92, -3.59) |
| Costa Rica | 0.03 (0.01, 0.05) | 0.03 (0.01, 0.05) |  | 5 (2, 11) | 16 (5, 34) |  | 0.71 (0.24,1.46) | 0.71 (0.21, 1.54) | -2.2 (-3.48, -0.91) |
| Croatia | 0.1 (0.05, 0.16) | 0.1 (0.04, 0.15) |  | 66 (30, 107) | 105 (46, 173) |  | 3.12 (1.42,5.06) | 2.94 (1.31, 4.84) | -0.06 (-0.29, 0.17) |
| Cuba | 0.03 (0.01, 0.05) | 0.03 (0.01, 0.05) |  | 25 (9, 48) | 64 (21, 127) |  | 0.53 (0.19,1) | 0.7 (0.23, 1.4) | 0.3 (-0.61, 1.22) |
| Cyprus | 0.12 (0.05, 0.18) | 0.11 (0.05, 0.17) |  | 16 (7, 27) | 29 (12, 47) |  | 6.06 (2.73,9.99) | 3.91 (1.67, 6.48) | -1.7 (-2.1, -1.3) |
| Czechia | 0.08 (0.03, 0.13) | 0.08 (0.04, 0.13) |  | 139 (61, 228) | 187 (80, 314) |  | 2.8 (1.23,4.57) | 2.08 (0.89, 3.51) | -0.1 (-0.69, 0.51) |
| Côte d'Ivoire | 0.02 (0.01, 0.05) | 0.07 (0.03, 0.11) |  | 19 (6, 37) | 94 (37, 165) |  | 1.33 (0.47,2.65) | 2.53 (1.03, 4.45) | 2.2 (0.27, 4.18) |
| Democratic People's Republic of Korea | 0.08 (0.04, 0.13) | 0.13 (0.06, 0.2) |  | 571 (234, 967) | 1764 (752, 2781) | | 16.17 (6.67,27.48) | 19.58 (8.46, 30.73) | 0.46 (0.14, 0.78) |
| Democratic Republic of the Congo | 0.06 (0.03, 0.1) | 0.09 (0.04, 0.14) |  | 204 (74, 364) | 508 (191, 906) |  | 3.94 (1.49,7.04) | 4.83 (1.87, 8.44) | 1.18 (-0.23, 2.61) |
| Denmark | 0.03 (0.01, 0.06) | 0.04 (0.02, 0.07) |  | 58 (22, 107) | 87 (33, 157) |  | 1.68 (0.62,3.09) | 1.6 (0.61, 2.92) | -1.21 (-1.85, -0.58) |
| Djibouti | 0.03 (0.01, 0.06) | 0.08 (0.03, 0.13) |  | 1 (0, 1) | 5 (2, 9) |  | 1.31 (0.38,2.86) | 2.24 (0.82, 4.45) | 1.78 (1.02, 2.55) |
| Dominica | 0.02 (0, 0.07) | 0 (0, 0.01) |  | 0 (0, 1) | 0 (0, 0) |  | 0.61 (0.01,1.89) | 0.03 (0, 0.24) | -11.34 (-13.43, -9.2) |
| Dominican Republic | 0.04 (0.02, 0.07) | 0.02 (0.01, 0.05) |  | 10 (4, 17) | 20 (6, 41) |  | 0.67 (0.27,1.21) | 0.5 (0.16, 1.04) | -0.78 (-1.55, 0.01) |
| Ecuador | 0.01 (0, 0.03) | 0.02 (0.01, 0.04) |  | 8 (3, 16) | 34 (11, 67) |  | 0.39 (0.12,0.76) | 0.62 (0.21, 1.24) | 0.93 (0.03, 1.83) |
| Egypt | 0.13 (0.06, 0.19) | 0.11 (0.05, 0.18) |  | 514 (222, 853) | 1046 (404, 1877) | | 4.41 (1.9,7.26) | 3.58 (1.36, 6.32) | -0.6 (-0.86, -0.33) |
| El Salvador | 0.07 (0.03, 0.12) | 0.06 (0.02, 0.1) |  | 22 (9, 36) | 31 (12, 55) |  | 1.78 (0.77,2.98) | 1.14 (0.43, 2.06) | -2.85 (-3.66, -2.03) |
| Equatorial Guinea | 0.01 (0, 0.03) | 0.06 (0.03, 0.11) |  | 1 (0, 2) | 3 (1, 6) |  | 1.04 (0.28,2.35) | 2.43 (0.85, 4.44) | 2.73 (0.55, 4.97) |
| Eritrea | 0.05 (0.02, 0.09) | 0.07 (0.03, 0.11) |  | 10 (3, 19) | 25 (10, 45) |  | 3.65 (1.33,7.08) | 3.4 (1.36, 6.05) | -0.14 (-0.5, 0.23) |
| Estonia | 0.03 (0.01, 0.06) | 0.03 (0.01, 0.06) |  | 5 (2, 9) | 4 (1, 9) |  | 0.71 (0.24,1.35) | 0.45 (0.15, 0.91) | -1.78 (-2.42, -1.13) |
| Eswatini | 0.02 (0.01, 0.04) | 0.05 (0.02, 0.09) |  | 2 (0, 3) | 6 (2, 11) |  | 1.85 (0.56,3.77) | 3.87 (1.42, 7.1) | 3.36 (1.81, 4.93) |
| Ethiopia | 0.04 (0.02, 0.08) | 0.08 (0.03, 0.12) |  | 232 (93, 438) | 465 (197, 800) |  | 2.91 (1.19,5.48) | 2.83 (1.21, 4.88) | -0.25 (-1.4, 0.9) |
| Fiji | 0 (0, 0.01) | 0.01 (0, 0.02) |  | 0 (0, 1) | 1 (0, 2) |  | 0.24 (0.02,0.7) | 0.35 (0.05, 1) | 0.26 (-1.15, 1.69) |
| Finland | 0.02 (0.01, 0.04) | 0.02 (0.01, 0.04) |  | 15 (5, 28) | 20 (7, 40) |  | 0.6 (0.21,1.14) | 0.35 (0.11, 0.7) | -3.13 (-4.41, -1.84) |
| France | 0.08 (0.03, 0.12) | 0.06 (0.03, 0.11) |  | 792 (366, 1298) | 636 (267, 1085) |  | 2.65 (1.22,4.36) | 0.97 (0.41, 1.64) | -3.55 (-3.94, -3.15) |
| Gabon | 0.01 (0, 0.02) | 0.03 (0.01, 0.06) |  | 1 (0, 2) | 4 (1, 8) |  | 0.35 (0.07,1.03) | 1.21 (0.42, 2.36) | 5.06 (2.41, 7.77) |
| Gambia | 0.03 (0.01, 0.06) | 0.07 (0.03, 0.12) |  | 2 (1, 4) | 10 (4, 19) |  | 1.49 (0.46,3.12) | 2.88 (1.13, 5.22) | 2.47 (1.57, 3.37) |
| Georgia | 0.09 (0.04, 0.14) | 0.1 (0.04, 0.16) |  | 34 (15, 56) | 57 (25, 94) |  | 1.99 (0.88,3.24) | 2.48 (1.08, 4.12) | 2.61 (1.77, 3.46) |
| Germany | 0.05 (0.02, 0.08) | 0.06 (0.02, 0.1) |  | 901 (372, 1575) | 1329 (555, 2273) | | 2.07 (0.85,3.61) | 1.48 (0.62, 2.53) | -1.57 (-1.95, -1.19) |
| Ghana | 0.04 (0.02, 0.07) | 0.1 (0.05, 0.16) |  | 37 (13, 72) | 244 (89, 417) |  | 1.81 (0.61,3.38) | 4.79 (1.74, 8.07) | 4.13 (2.52, 5.77) |
| Greece | 0.1 (0.05, 0.16) | 0.09 (0.04, 0.14) |  | 140 (62, 216) | 274 (118, 447) |  | 2.23 (1,3.47) | 2.01 (0.87, 3.29) | 0.06 (-0.48, 0.61) |
| Greenland | 0.04 (0.02, 0.07) | 0.04 (0.01, 0.06) |  | 0 (0, 1) | 0 (0, 1) |  | 2.72 (1.02,4.9) | 1.57 (0.63, 2.86) | -1.65 (-2.42, -0.88) |
| Grenada | 0.02 (0, 0.06) | 0.01 (0, 0.03) |  | 0 (0, 0) | 0 (0, 0) |  | 0.61 (0.13,1.42) | 0.23 (0.02, 0.68) | -4.83 (-6.22, -3.41) |
| Guam | 0 (0, 0.01) | 0 (0, 0.01) |  | 0 (0, 0) | 0 (0, 0) |  | 0.09 (0,0.82) | 0.02 (0, 0.21) | -6.47 (-10.06, -2.74) |
| Guatemala | 0.04 (0.02, 0.07) | 0.03 (0.01, 0.05) |  | 15 (6, 27) | 28 (10, 53) |  | 1.22 (0.48,2.15) | 0.78 (0.3, 1.48) | -3.5 (-4.39, -2.59) |
| Guinea | 0.03 (0.01, 0.05) | 0.08 (0.03, 0.13) |  | 18 (6, 37) | 72 (28, 124) |  | 1.36 (0.47,2.75) | 3.2 (1.25, 5.46) | 3.52 (2.36, 4.69) |
| Guinea-Bissau | 0.02 (0.01, 0.04) | 0.07 (0.03, 0.12) |  | 3 (1, 6) | 9 (3, 16) |  | 1.81 (0.52,3.72) | 3.62 (1.39, 6.47) | 2.88 (1.68, 4.09) |
| Guyana | 0 (0, 0.01) | 0 (0, 0.01) |  | 0 (0, 0) | 0 (0, 1) |  | 0.06 (0.01,0.17) | 0.06 (0, 0.24) | -0.5 (-2.3, 1.34) |
| Haiti | 0.06 (0.02, 0.1) | 0.04 (0.02, 0.08) |  | 40 (14, 76) | 56 (18, 113) |  | 3.61 (1.26,6.75) | 2.29 (0.74, 4.6) | -2.27 (-2.92, -1.62) |
| Honduras | 0.04 (0.01, 0.07) | 0.03 (0.01, 0.06) |  | 16 (6, 28) | 52 (19, 100) |  | 1.89 (0.72,3.46) | 2.24 (0.81, 4.23) | -0.09 (-1.02, 0.85) |
| Hungary | 0.09 (0.04, 0.15) | 0.07 (0.03, 0.11) |  | 279 (124, 442) | 209 (88, 353) |  | 5.39 (2.41,8.59) | 2.79 (1.18, 4.73) | -1.93 (-2.38, -1.48) |
| Iceland | 0.02 (0.01, 0.04) | 0.03 (0.01, 0.06) |  | 1 (0, 1) | 1 (0, 3) |  | 0.43 (0.13,0.91) | 0.55 (0.18, 1.08) | 0.81 (0.07, 1.55) |
| India | 0.1 (0.05, 0.16) | 0.19 (0.09, 0.28) |  | 26759 (11551, 43154) | 92560 (42900, 146648) | | 17.59 (7.38,28.61) | 21.21 (9.82, 33.12) | 0.53 (0.31, 0.76) |
| Indonesia | 0.01 (0, 0.02) | 0.05 (0.02, 0.08) |  | 247 (79, 498) | 2334 (990, 4045) | | 0.75 (0.24,1.51) | 3.4 (1.48, 5.78) | 4.98 (3.68, 6.29) |
| Iran (Islamic Republic of) | 0.12 (0.06, 0.18) | 0.14 (0.07, 0.22) |  | 307 (137, 491) | 1105 (519, 1708) | | 3.41 (1.53,5.44) | 3.47 (1.64, 5.38) | 0.32 (0.08, 0.56) |
| Iraq | 0.14 (0.07, 0.21) | 0.15 (0.07, 0.23) |  | 75 (32, 127) | 168 (78, 267) |  | 2.42 (1.04,4.05) | 2.11 (0.99, 3.33) | -0.91 (-1.22, -0.61) |
| Ireland | 0.03 (0.01, 0.06) | 0.01 (0, 0.02) |  | 43 (16, 78) | 8 (2, 18) |  | 2.64 (1.01,4.79) | 0.25 (0.07, 0.55) | -7.77 (-8.71, -6.81) |
| Israel | 0.14 (0.06, 0.21) | 0.12 (0.05, 0.18) |  | 84 (40, 132) | 108 (47, 170) |  | 4.11 (1.93,6.39) | 2.01 (0.89, 3.18) | -2.2 (-2.52, -1.88) |
| Italy | 0.14 (0.07, 0.21) | 0.12 (0.06, 0.19) |  | 1850 (870, 2818) | 2049 (945, 3158) | | 5.56 (2.63,8.45) | 2.91 (1.34, 4.48) | -2.57 (-3.01, -2.12) |
| Jamaica | 0.07 (0.03, 0.12) | 0.05 (0.02, 0.09) |  | 13 (6, 22) | 20 (8, 37) |  | 1.62 (0.71,2.75) | 1.44 (0.54, 2.65) | -1.24 (-2.31, -0.15) |
| Japan | 0.05 (0.02, 0.08) | 0.08 (0.04, 0.13) |  | 583 (242, 1013) | 1991 (816, 3386) | | 1.02 (0.42,1.77) | 1.03 (0.42, 1.75) | -0.26 (-0.84, 0.33) |
| Jordan | 0.13 (0.06, 0.21) | 0.13 (0.06, 0.2) |  | 21 (9, 33) | 51 (23, 85) |  | 4.39 (1.95,7.11) | 2.09 (0.98, 3.44) | -2.88 (-3.12, -2.64) |
| Kazakhstan | 0.09 (0.04, 0.14) | 0.07 (0.03, 0.12) |  | 292 (129, 461) | 347 (149, 594) |  | 7.72 (3.42,12.22) | 6.79 (2.84, 11.47) | -0.78 (-1.49, -0.06) |
| Kenya | 0.01 (0, 0.02) | 0.05 (0.02, 0.08) |  | 13 (4, 29) | 148 (55, 267) |  | 0.41 (0.12,0.94) | 2.12 (0.78, 3.84) | 6.46 (4.46, 8.49) |
| Kiribati | 0.02 (0.01, 0.04) | 0.06 (0.02, 0.09) |  | 0 (0, 1) | 1 (0, 2) |  | 2.58 (0.92,4.95) | 5.47 (2.34, 9.31) | 2.52 (1.27, 3.79) |
| Kuwait | 0.14 (0.07, 0.22) | 0.17 (0.08, 0.25) |  | 4 (2, 6) | 19 (8, 32) |  | 1.89 (0.89,2.97) | 1.81 (0.79, 3.01) | -0.15 (-0.53, 0.24) |
| Kyrgyzstan | 0.13 (0.06, 0.2) | 0.13 (0.06, 0.2) |  | 176 (81, 269) | 105 (48, 165) |  | 17.72 (8.22,27.27) | 7.29 (3.37, 11.49) | -3.68 (-4.32, -3.03) |
| Lao People's Democratic Republic | 0.02 (0.01, 0.04) | 0.06 (0.03, 0.1) |  | 20 (7, 41) | 73 (29, 128) |  | 2.7 (0.9,5.49) | 4.87 (1.94, 8.71) | 2.06 (1.19, 2.94) |
| Latvia | 0.03 (0.01, 0.06) | 0.03 (0.01, 0.06) |  | 12 (4, 22) | 7 (2, 13) |  | 1.03 (0.36,1.88) | 0.48 (0.17, 0.97) | -2.82 (-3.57, -2.06) |
| Lebanon | 0.12 (0.06, 0.19) | 0.1 (0.05, 0.17) |  | 28 (12, 47) | 54 (20, 96) |  | 3.36 (1.45,5.62) | 2.39 (0.9, 4.28) | -0.53 (-0.86, -0.2) |
| Lesotho | 0.03 (0.01, 0.05) | 0.06 (0.03, 0.11) |  | 10 (3, 21) | 25 (10, 46) |  | 3.27 (1.07,6.7) | 6.67 (2.69, 12.09) | 2.81 (1.84, 3.8) |
| Liberia | 0.01 (0, 0.02) | 0.06 (0.02, 0.1) |  | 1 (0, 3) | 9 (3, 16) |  | 0.25 (0.07,0.53) | 1.18 (0.43, 2.13) | 5.77 (3.64, 7.94) |
| Libya | 0.1 (0.05, 0.15) | 0.12 (0.06, 0.18) |  | 18 (7, 31) | 60 (26, 104) |  | 2.26 (0.9,3.9) | 2.79 (1.23, 4.79) | 0.49 (-0.04, 1.02) |
| Lithuania | 0.04 (0.02, 0.07) | 0.02 (0.01, 0.04) |  | 37 (14, 64) | 10 (4, 21) |  | 2.28 (0.86,3.98) | 0.5 (0.17, 0.99) | -5.14 (-5.92, -4.36) |
| Luxembourg | 0.05 (0.02, 0.09) | 0.06 (0.03, 0.1) |  | 5 (2, 8) | 7 (3, 11) |  | 2.45 (0.94,4.35) | 1.49 (0.6, 2.54) | -1.87 (-2.4, -1.34) |
| Madagascar | 0 (0, 0.01) | 0.03 (0.01, 0.05) |  | 5 (1, 12) | 61 (22, 117) |  | 0.25 (0.06,0.61) | 1.79 (0.65, 3.39) | 7.88 (5.38, 10.44) |
| Malawi | 0.01 (0, 0.03) | 0.05 (0.02, 0.08) |  | 8 (3, 18) | 42 (16, 77) |  | 0.64 (0.21,1.33) | 1.86 (0.74, 3.37) | 4.91 (3.35, 6.49) |
| Malaysia | 0.02 (0.01, 0.04) | 0.06 (0.02, 0.09) |  | 49 (16, 94) | 231 (101, 403) |  | 1.4 (0.47,2.69) | 2.19 (0.95, 3.82) | -0.38 (-1.61, 0.86) |
| Maldives | 0.02 (0.01, 0.04) | 0.11 (0.05, 0.17) |  | 1 (0, 1) | 5 (2, 8) |  | 2.07 (0.58,4.56) | 4.16 (1.85, 6.81) | 1.61 (0.9, 2.31) |
| Mali | 0.03 (0.01, 0.05) | 0.07 (0.03, 0.12) |  | 24 (8, 47) | 112 (46, 199) |  | 1.42 (0.54,2.73) | 3.01 (1.26, 5.26) | 2.67 (1.27, 4.09) |
| Malta | 0.16 (0.07, 0.23) | 0.13 (0.06, 0.2) |  | 11 (5, 16) | 12 (5, 20) |  | 6.77 (3.08,10.24) | 2.88 (1.26, 4.71) | -3.84 (-4.39, -3.28) |
| Marshall Islands | 0 (0, 0) | 0 (0, 0) |  | 0 (0, 0) | 0 (0, 0) |  | 0.06 (0,0.55) | 0.01 (0, 0.11) | -6.45 (-10.08, -2.68) |
| Mauritania | 0.02 (0.01, 0.04) | 0.05 (0.02, 0.09) |  | 3 (1, 7) | 10 (4, 18) |  | 0.92 (0.32,1.74) | 1.16 (0.46, 2.15) | 0.7 (-0.64, 2.06) |
| Mauritius | 0 (0, 0.01) | 0 (0, 0) |  | 0 (0, 1) | 0 (0, 0) |  | 0.17 (0,0.48) | 0.01 (0, 0.04) | -11.76 (-15.15, -8.23) |
| Mexico | 0.15 (0.07, 0.22) | 0.07 (0.03, 0.11) |  | 1115 (549, 1652) | 1225 (536, 2054) | | 6.95 (3.36,10.36) | 2.6 (1.13, 4.36) | -3.87 (-4.14, -3.6) |
| Micronesia (Federated States of) | 0 (0, 0.01) | 0 (0, 0) |  | 0 (0, 0) | 0 (0, 0) |  | 0.23 (0,1.44) | 0.02 (0, 0.29) | -6.87 (-10.51, -3.09) |
| Monaco | 0.12 (0.03, 0.22) | 0.1 (0.04, 0.17) |  | 1 (0, 2) | 1 (0, 2) |  | 3 (0.77,5.62) | 1.83 (0.74, 3.29) | -0.88 (-1.55, -0.21) |
| Mongolia | 0.09 (0.04, 0.14) | 0.07 (0.03, 0.11) |  | 16 (7, 27) | 12 (5, 20) |  | 4.6 (1.97,7.69) | 1.83 (0.76, 3.11) | -3.99 (-4.7, -3.27) |
| Montenegro | 0.1 (0.05, 0.16) | 0.09 (0.04, 0.14) |  | 3 (1, 5) | 5 (2, 8) |  | 1.54 (0.67,2.5) | 1.27 (0.54, 2.11) | -0.68 (-1.13, -0.23) |
| Morocco | 0.05 (0.02, 0.09) | 0.11 (0.05, 0.17) |  | 77 (30, 144) | 427 (184, 707) |  | 1.53 (0.6,2.86) | 3.55 (1.53, 5.85) | 2.91 (2.35, 3.47) |
| Mozambique | 0.01 (0, 0.02) | 0.04 (0.01, 0.06) |  | 7 (2, 15) | 54 (20, 101) |  | 0.34 (0.09,0.75) | 1.57 (0.61, 2.93) | 6.74 (4.95, 8.55) |
| Myanmar | 0.03 (0.01, 0.06) | 0.06 (0.03, 0.1) |  | 406 (158, 779) | 1224 (503, 2066) | | 5.11 (2,9.7) | 8.31 (3.4, 14.06) | 1.42 (0.83, 2) |
| Namibia | 0.06 (0.03, 0.1) | 0.07 (0.03, 0.12) |  | 16 (6, 28) | 24 (10, 42) |  | 5.93 (2.18,10.59) | 5.26 (2.13, 9.19) | 0.16 (-0.68, 1.02) |
| Nauru | 0 (0, 0) | 0 (0, 0) |  | 0 (0, 0) | 0 (0, 0) |  | 0.04 (0,0) | 0.02 (0, 0) | -3.5 (-6.26, -0.66) |
| Nepal | 0.13 (0.06, 0.2) | 0.19 (0.09, 0.28) |  | 979 (421, 1618) | 3110 (1427, 4834) | | 29.13 (12.49,47.68) | 38 (16.93, 59.13) | 1.04 (0.84, 1.25) |
| Netherlands | 0.04 (0.02, 0.07) | 0.04 (0.02, 0.08) |  | 175 (68, 318) | 233 (90, 417) |  | 2.35 (0.92,4.24) | 1.52 (0.59, 2.71) | -2.47 (-3.24, -1.7) |
| New Zealand | 0.01 (0, 0.01) | 0.01 (0, 0.03) |  | 5 (1, 12) | 16 (5, 33) |  | 0.32 (0.06,0.75) | 0.42 (0.13, 0.87) | 0 (-1.34, 1.35) |
| Nicaragua | 0.02 (0.01, 0.04) | 0.03 (0.01, 0.05) |  | 4 (1, 7) | 17 (6, 33) |  | 0.81 (0.29,1.55) | 1.26 (0.42, 2.43) | 1.01 (0.26, 1.77) |
| Niger | 0.06 (0.03, 0.1) | 0.09 (0.04, 0.14) |  | 33 (12, 66) | 92 (37, 180) |  | 3.27 (1.25,6.31) | 3.46 (1.37, 6.61) | 0.86 (-0.33, 2.07) |
| Nigeria | 0.08 (0.04, 0.14) | 0.12 (0.05, 0.18) |  | 463 (184, 805) | 1030 (420, 1789) | | 2.96 (1.16,5.03) | 3.41 (1.4, 5.8) | 0.88 (0.16, 1.61) |
| Niue | 0.02 (0.01, 0.04) | 0.02 (0.01, 0.04) |  | 0 (0, 0) | 0 (0, 0) |  | 1.88 (0.47,4.17) | 1.27 (0.32, 2.77) | -3.09 (-3.8, -2.39) |
| North Macedonia | 0.1 (0.05, 0.16) | 0.03 (0.01, 0.06) |  | 31 (14, 50) | 12 (5, 22) |  | 4.32 (1.95,6.92) | 1.04 (0.38, 1.86) | -4.17 (-5.31, -3.03) |
| Northern Mariana Islands | 0.04 (0.01, 0.08) | 0.08 (0.04, 0.14) |  | 0 (0, 0) | 1 (0, 1) |  | 3.33 (0.99,6.77) | 4.78 (2.01, 8.38) | 0.59 (-0.23, 1.41) |
| Norway | 0.04 (0.02, 0.07) | 0.03 (0.01, 0.05) |  | 28 (12, 50) | 32 (12, 60) |  | 0.95 (0.39,1.67) | 0.71 (0.25, 1.32) | -1.45 (-2.5, -0.4) |
| Oman | 0.09 (0.04, 0.15) | 0.11 (0.05, 0.18) |  | 9 (3, 17) | 14 (6, 23) |  | 4.31 (1.72,8.05) | 3.33 (1.44, 5.41) | -0.11 (-0.53, 0.3) |
| Pakistan | 0.12 (0.06, 0.18) | 0.18 (0.08, 0.26) |  | 4241 (1864, 6949) | 7855 (3713, 12261) | | 15.74 (6.87,25.84) | 19.72 (9.42, 30.51) | 0.75 (0.52, 0.98) |
| Palau | 0 (0, 0.01) | 0 (0, 0.01) |  | 0 (0, 0) | 0 (0, 0) |  | 0.21 (0,1.4) | 0.08 (0, 0.68) | -3.02 (-6.13, 0.2) |
| Palestine | 0.14 (0.06, 0.2) | 0.12 (0.06, 0.19) |  | 17 (8, 31) | 24 (11, 39) |  | 5.43 (2.45,9.66) | 3.41 (1.6, 5.48) | -1.59 (-1.79, -1.38) |
| Panama | 0.04 (0.02, 0.07) | 0.03 (0.01, 0.06) |  | 6 (2, 11) | 11 (4, 22) |  | 0.94 (0.37,1.7) | 0.57 (0.19, 1.14) | -4.32 (-5.62, -3.02) |
| Papua New Guinea | 0 (0, 0) | 0 (0, 0) |  | 1 (0, 3) | 1 (0, 3) |  | 0.14 (0.03,0.42) | 0.07 (0.01, 0.19) | -3.97 (-6.93, -0.91) |
| Paraguay | 0.03 (0.01, 0.06) | 0.06 (0.02, 0.1) |  | 7 (3, 12) | 38 (14, 67) |  | 0.76 (0.29,1.43) | 1.67 (0.64, 2.96) | 3.48 (2.35, 4.63) |
| Peru | 0.01 (0, 0.02) | 0.03 (0.01, 0.06) |  | 7 (2, 16) | 49 (19, 94) |  | 0.14 (0.04,0.31) | 0.32 (0.12, 0.62) | 3.16 (2.12, 4.21) |
| Philippines | 0.01 (0, 0.01) | 0.01 (0, 0.01) |  | 43 (11, 96) | 87 (24, 194) |  | 0.43 (0.11,0.92) | 0.32 (0.09, 0.71) | -0.11 (-1.75, 1.56) |
| Poland | 0.06 (0.03, 0.11) | 0.06 (0.03, 0.11) |  | 514 (216, 849) | 422 (178, 714) |  | 3.27 (1.38,5.39) | 1.53 (0.65, 2.59) | -2.81 (-3.19, -2.42) |
| Portugal | 0.04 (0.02, 0.07) | 0.05 (0.02, 0.09) |  | 85 (35, 148) | 169 (66, 300) |  | 1.75 (0.73,3.02) | 1.5 (0.58, 2.69) | 0.45 (-0.65, 1.56) |
| Puerto Rico | 0.02 (0.01, 0.04) | 0 (0, 0.01) |  | 8 (2, 16) | 3 (1, 8) |  | 0.5 (0.16,1.01) | 0.09 (0.02, 0.24) | -7.81 (-9.54, -6.04) |
| Qatar | 0.15 (0.07, 0.23) | 0.19 (0.09, 0.28) |  | 1 (1, 2) | 7 (3, 11) |  | 4.55 (1.9,8.29) | 3.68 (1.87, 6.19) | -0.67 (-1.24, -0.09) |
| Republic of Korea | 0.08 (0.03, 0.13) | 0.15 (0.07, 0.22) |  | 202 (86, 341) | 957 (451, 1520) |  | 3.21 (1.38,5.49) | 3.41 (1.59, 5.43) | -1.06 (-2.09, -0.03) |
| Republic of Moldova | 0.1 (0.05, 0.16) | 0.04 (0.02, 0.07) |  | 107 (49, 167) | 24 (9, 44) |  | 6.99 (3.18,10.94) | 1.1 (0.43, 2.02) | -6.62 (-7.29, -5.94) |
| Romania | 0.1 (0.05, 0.15) | 0.02 (0.01, 0.04) |  | 779 (339, 1238) | 95 (31, 185) |  | 7.5 (3.28,11.95) | 0.61 (0.2, 1.19) | -7.95 (-8.59, -7.3) |
| Russian Federation | 0.08 (0.03, 0.12) | 0.04 (0.01, 0.06) |  | 2240 (954, 3597) | 837 (328, 1486) |  | 4.67 (2.01,7.49) | 1.08 (0.42, 1.91) | -5.34 (-5.87, -4.8) |
| Rwanda | 0.04 (0.01, 0.06) | 0.09 (0.04, 0.14) |  | 33 (12, 65) | 80 (33, 136) |  | 3.3 (1.2,6.53) | 4.73 (1.98, 8.07) | 1.31 (-0.44, 3.1) |
| Saint Kitts and Nevis | 0.02 (0, 0.07) | 0 (0, 0) |  | 0 (0, 0) | 0 (0, 0) |  | 0.58 (0.03,1.73) | 0 (0, 0) | -25.25 (-28.66, -21.69) |
| Saint Lucia | 0.02 (0, 0.07) | 0 (0, 0.01) |  | 0 (0, 1) | 0 (0, 0) |  | 0.86 (0,3.34) | 0.04 (0, 0.53) | -11.13 (-12.76, -9.47) |
| Saint Vincent and the Grenadines | 0.02 (0, 0.07) | 0 (0, 0.02) |  | 0 (0, 0) | 0 (0, 0) |  | 0.3 (0,1.11) | 0.03 (0, 0.33) | -9.71 (-11.38, -8.01) |
| Samoa | 0.02 (0, 0.08) | 0.02 (0.01, 0.04) |  | 1 (0, 3) | 1 (0, 2) |  | 2.7 (0,10.53) | 1.45 (0.38, 3) | -3.58 (-4.19, -2.97) |
| San Marino | 0.14 (0.06, 0.23) | 0.11 (0.05, 0.19) |  | 0 (0, 1) | 1 (0, 1) |  | 2.68 (1.07,4.42) | 1.62 (0.67, 2.83) | -1.69 (-2.36, -1.02) |
| Sao Tome and Principe | 0.02 (0, 0.05) | 0.06 (0.02, 0.11) |  | 0 (0, 1) | 2 (1, 3) |  | 1.47 (0.26,4.44) | 4.44 (1.6, 8.47) | 4.53 (3.1, 5.97) |
| Saudi Arabia | 0.11 (0.05, 0.17) | 0.15 (0.07, 0.22) |  | 95 (39, 178) | 200 (89, 316) |  | 4.12 (1.72,7.77) | 3.11 (1.39, 4.95) | -1.07 (-1.33, -0.81) |
| Senegal | 0.03 (0.01, 0.05) | 0.06 (0.03, 0.1) |  | 17 (6, 36) | 62 (26, 108) |  | 1.38 (0.46,2.82) | 2.22 (0.93, 3.87) | 1.98 (0.94, 3.03) |
| Serbia | 0.1 (0.05, 0.16) | 0.03 (0.01, 0.06) |  | 178 (79, 290) | 70 (26, 123) |  | 3.99 (1.81,6.52) | 1.04 (0.39, 1.84) | -4.12 (-4.95, -3.29) |
| Seychelles | 0 (0, 0.03) | 0.02 (0, 0.09) |  | 0 (0, 0) | 0 (0, 1) |  | 0.11 (0,1.52) | 0.66 (0, 3.37) | 5.43 (2.87, 8.05) |
| Sierra Leone | 0.03 (0.01, 0.05) | 0.06 (0.03, 0.1) |  | 11 (4, 21) | 29 (11, 54) |  | 1.29 (0.45,2.46) | 2.15 (0.83, 3.95) | 2.45 (1.2, 3.72) |
| Singapore | 0.03 (0, 0.09) | 0.07 (0.02, 0.14) |  | 17 (1, 50) | 30 (9, 61) |  | 2.58 (0.08,7.5) | 1.01 (0.29, 2.06) | -3.99 (-5.81, -2.14) |
| Slovakia | 0.09 (0.04, 0.14) | 0.08 (0.03, 0.12) |  | 50 (23, 84) | 50 (22, 86) |  | 2.26 (1.02,3.76) | 1.45 (0.64, 2.5) | -0.75 (-1.3, -0.19) |
| Slovenia | 0.11 (0.05, 0.17) | 0.11 (0.05, 0.17) |  | 46 (20, 78) | 40 (18, 67) |  | 5.49 (2.41,9.28) | 2.22 (0.99, 3.73) | -4.3 (-4.78, -3.82) |
| Solomon Islands | 0 (0, 0) | 0 (0, 0) |  | 0 (0, 0) | 0 (0, 0) |  | 0.1 (0,0.39) | 0.05 (0, 0.27) | -2.58 (-5.5, 0.42) |
| Somalia | 0.01 (0, 0.02) | 0.04 (0.02, 0.07) |  | 5 (2, 12) | 49 (17, 106) |  | 0.62 (0.19,1.41) | 2.63 (0.9, 5.54) | 4.57 (3.34, 5.82) |
| South Africa | 0.03 (0.01, 0.05) | 0.05 (0.02, 0.08) |  | 103 (38, 189) | 348 (151, 589) |  | 1.46 (0.53,2.7) | 2.45 (1.05, 4.18) | 1.67 (0.82, 2.53) |
| South Sudan | 0.09 (0.04, 0.14) | 0.07 (0.03, 0.11) |  | 47 (19, 81) | 39 (15, 71) |  | 4.71 (1.9,8.15) | 2.64 (1.02, 4.83) | -1.72 (-2.48, -0.96) |
| Spain | 0.05 (0.02, 0.09) | 0.09 (0.04, 0.15) |  | 690 (296, 1153) | 1814 (805, 2905) | | 3.37 (1.46,5.66) | 3.85 (1.71, 6.15) | 0.24 (-0.25, 0.74) |
| Sri Lanka | 0.01 (0, 0.02) | 0.06 (0.02, 0.09) |  | 13 (4, 30) | 174 (65, 321) |  | 0.31 (0.09,0.71) | 1.94 (0.73, 3.57) | 6.13 (5.24, 7.04) |
| Sudan | 0.05 (0.02, 0.08) | 0.09 (0.04, 0.13) |  | 94 (29, 186) | 260 (95, 473) |  | 2.44 (0.73,4.77) | 3.22 (1.17, 5.69) | 1.16 (0.59, 1.73) |
| Suriname | 0 (0, 0) | 0 (0, 0.01) |  | 0 (0, 0) | 0 (0, 0) |  | 0.05 (0.01,0.11) | 0.07 (0.01, 0.19) | -0.02 (-1.96, 1.96) |
| Sweden | 0.04 (0.01, 0.06) | 0.03 (0.01, 0.05) |  | 40 (15, 73) | 51 (18, 95) |  | 0.61 (0.23,1.11) | 0.47 (0.16, 0.88) | -1.42 (-2.32, -0.52) |
| Switzerland | 0.1 (0.05, 0.16) | 0.09 (0.04, 0.15) |  | 138 (64, 218) | 139 (61, 222) |  | 3.35 (1.55,5.26) | 1.65 (0.73, 2.62) | -2.44 (-2.78, -2.09) |
| Syrian Arab Republic | 0.13 (0.06, 0.2) | 0.12 (0.06, 0.18) |  | 75 (34, 128) | 142 (62, 242) |  | 3.2 (1.43,5.47) | 2.8 (1.21, 4.75) | -0.59 (-0.78, -0.39) |
| Taiwan (Province of China) | 0.06 (0.02, 0.1) | 0.06 (0.03, 0.11) |  | 120 (49, 202) | 382 (167, 680) |  | 2.26 (0.92,3.85) | 2.1 (0.91, 3.7) | -0.2 (-1.08, 0.68) |
| Tajikistan | 0.15 (0.07, 0.23) | 0.12 (0.05, 0.18) |  | 78 (37, 122) | 72 (33, 117) |  | 7.11 (3.32,11.11) | 4.65 (2.1, 7.65) | -1.02 (-1.5, -0.55) |
| Thailand | 0.02 (0.01, 0.03) | 0.08 (0.04, 0.13) |  | 167 (58, 332) | 1070 (448, 1829) | | 1.37 (0.48,2.72) | 2.6 (1.09, 4.45) | 2.08 (1.32, 2.85) |
| Timor-Leste | 0.01 (0, 0.02) | 0.03 (0.01, 0.05) |  | 1 (0, 2) | 6 (2, 13) |  | 0.91 (0.24,1.93) | 2.05 (0.61, 4.19) | 2.22 (0.61, 3.86) |
| Togo | 0.05 (0.02, 0.09) | 0.11 (0.05, 0.17) |  | 10 (4, 19) | 46 (19, 78) |  | 2.48 (0.97,4.45) | 4.47 (1.84, 7.58) | 2.45 (1.26, 3.66) |
| Tokelau | 0.02 (0.01, 0.04) | 0.02 (0.01, 0.04) |  | 0 (0, 0) | 0 (0, 0) |  | 1.81 (0.45,4.14) | 0.96 (0.23, 2.04) | -3.66 (-4.36, -2.95) |
| Tonga | 0.02 (0, 0.04) | 0.02 (0, 0.05) |  | 0 (0, 1) | 0 (0, 1) |  | 1.84 (0.41,4.25) | 1.44 (0.29, 3.16) | -2.46 (-3.27, -1.65) |
| Trinidad and Tobago | 0.01 (0, 0.04) | 0.01 (0, 0.02) |  | 1 (0, 3) | 1 (0, 3) |  | 0.33 (0.04,0.91) | 0.1 (0, 0.33) | -5.7 (-6.85, -4.53) |
| Tunisia | 0.12 (0.06, 0.19) | 0.12 (0.05, 0.18) |  | 58 (25, 98) | 139 (58, 238) |  | 3.14 (1.32,5.21) | 2.75 (1.12, 4.68) | -0.85 (-1.13, -0.57) |
| Turkey | 0.11 (0.05, 0.17) | 0.1 (0.05, 0.16) |  | 1033 (476, 1652) | 1886 (841, 3183) | | 7.36 (3.31,11.66) | 5.19 (2.3, 8.76) | -0.41 (-0.9, 0.09) |
| Turkmenistan | 0.11 (0.05, 0.17) | 0.09 (0.04, 0.14) |  | 35 (16, 54) | 18 (8, 30) |  | 5.84 (2.64,8.9) | 1.39 (0.62, 2.32) | -5.75 (-6.39, -5.1) |
| Tuvalu | 0 (0, 0.01) | 0 (0, 0.01) |  | 0 (0, 0) | 0 (0, 0) |  | 0.33 (0,1.31) | 0.36 (0, 1.15) | -0.45 (-2.16, 1.29) |
| Uganda | 0.04 (0.02, 0.07) | 0.07 (0.03, 0.11) |  | 65 (24, 131) | 145 (55, 257) |  | 2.74 (1,5.53) | 3.4 (1.33, 5.95) | 0.7 (-1.28, 2.71) |
| Ukraine | 0.09 (0.04, 0.15) | 0.05 (0.02, 0.08) |  | 1748 (752, 2793) | 352 (134, 623) |  | 8.2 (3.52,13.17) | 1.33 (0.51, 2.34) | -7.57 (-8.07, -7.07) |
| United Arab Emirates | 0.14 (0.07, 0.21) | 0.14 (0.06, 0.21) |  | 16 (6, 27) | 135 (52, 240) |  | 6.62 (2.82,11.32) | 4.81 (2.19, 7.94) | -1.04 (-1.6, -0.48) |
| United Kingdom | 0.02 (0.01, 0.05) | 0.01 (0, 0.03) |  | 496 (177, 924) | 308 (94, 631) |  | 1.44 (0.51,2.67) | 0.51 (0.15, 1.04) | -2.41 (-3.25, -1.57) |
| United Republic of Tanzania | 0.01 (0, 0.02) | 0.04 (0.02, 0.07) |  | 16 (5, 32) | 111 (45, 197) |  | 0.39 (0.13,0.79) | 1.28 (0.51, 2.23) | 4.89 (3.17, 6.65) |
| United States of America | 0.1 (0.05, 0.15) | 0.07 (0.03, 0.11) |  | 5060 (2313, 7899) | 6313 (2732, 10509) | | 3.84 (1.75,6.01) | 2.47 (1.07, 4.11) | -1.67 (-2.12, -1.23) |
| United States Virgin Islands | 0.02 (0.01, 0.04) | 0 (0, 0) |  | 0 (0, 0) | 0 (0, 0) |  | 0.25 (0.08,0.56) | 0.03 (0, 0.09) | -9.39 (-11.48, -7.24) |
| Uruguay | 0.01 (0, 0.03) | 0.02 (0.01, 0.04) |  | 11 (3, 23) | 23 (6, 48) |  | 0.67 (0.21,1.48) | 0.99 (0.27, 2.07) | 0.82 (0.13, 1.51) |
| Uzbekistan | 0.14 (0.06, 0.21) | 0.11 (0.05, 0.17) |  | 225 (98, 370) | 130 (60, 207) |  | 5.7 (2.5,9.39) | 2.49 (1.15, 3.95) | -3.94 (-4.58, -3.3) |
| Vanuatu | 0 (0, 0.01) | 0.01 (0, 0.02) |  | 0 (0, 0) | 0 (0, 1) |  | 0.6 (0.07,1.7) | 0.59 (0.09, 1.6) | -0.67 (-2.24, 0.93) |
| Venezuela (Bolivarian Republic of) | 0.03 (0.01, 0.06) | 0.03 (0.01, 0.05) |  | 28 (11, 50) | 93 (35, 178) |  | 0.74 (0.29,1.35) | 0.79 (0.3, 1.52) | -1.61 (-2.49, -0.72) |
| Viet Nam | 0.02 (0.01, 0.04) | 0.05 (0.02, 0.08) |  | 233 (58, 475) | 840 (220, 1581) |  | 1.85 (0.46,3.78) | 3.05 (0.81, 5.71) | 2.33 (1.59, 3.08) |
| Yemen | 0.05 (0.02, 0.09) | 0.09 (0.04, 0.15) |  | 45 (15, 90) | 190 (79, 330) |  | 2.9 (0.9,5.77) | 3.86 (1.58, 6.55) | 1.31 (0.9, 1.71) |
| Zambia | 0.05 (0.02, 0.08) | 0.07 (0.03, 0.11) |  | 25 (10, 46) | 66 (26, 111) |  | 2.25 (0.89,4.14) | 2.95 (1.17, 4.94) | 1.63 (0.61, 2.65) |
| Zimbabwe | 0.03 (0.01, 0.05) | 0.06 (0.03, 0.1) |  | 13 (5, 25) | 42 (17, 74) |  | 1.03 (0.37,1.93) | 2.06 (0.81, 3.7) | 3.53 (2.27, 4.79) |

(B) Women

| Country | Percentage (%) | |  | Death number | |  | Age-standardized mortality rate | | |
| --- | --- | --- | --- | --- | --- | --- | --- | --- | --- |
|  | 1990  (uncertainty interval) | 2019  (uncertainty interval) |  | 1990  (uncertainty interval) | 2019  (uncertainty interval) |  | 1990  (uncertainty interval)  (per 100,000 person-years) | 2019  (uncertainty interval)  (per 100,000 person-years) | Annual percentage change, 1990-2019 (confidence interval) (%) |
| Afghanistan | 0.13 (0.06, 0.2) | 0.12 (0.06, 0.18) |  | 154 (46, 291) | 233 (83, 410) |  | 5.5 (1.73,10.43) | 4.75 (1.65, 8.45) | -0.39 (-0.7, -0.07) |
| Albania | 0.11 (0.05, 0.17) | 0.04 (0.02, 0.07) |  | 28 (10, 46) | 8 (3, 16) |  | 2.79 (1.01,4.6) | 0.36 (0.13, 0.69) | -6.29 (-6.85, -5.72) |
| Algeria | 0.1 (0.05, 0.16) | 0.13 (0.06, 0.19) |  | 82 (31, 148) | 238 (104, 398) |  | 2.35 (0.86,4.21) | 2.19 (0.97, 3.66) | -0.23 (-0.53, 0.06) |
| American Samoa | 0.02 (0, 0.04) | 0.02 (0.01, 0.04) |  | 0 (0, 0) | 0 (0, 0) |  | 0.88 (0.23,1.91) | 0.62 (0.16, 1.35) | -2.87 (-3.53, -2.21) |
| Andorra | 0.07 (0.03, 0.13) | 0.09 (0.04, 0.15) |  | 0 (0, 0) | 1 (0, 2) |  | 1.03 (0.35,1.98) | 1.11 (0.44, 1.95) | 0.37 (0.08, 0.66) |
| Angola | 0.09 (0.04, 0.15) | 0.07 (0.03, 0.11) |  | 55 (19, 107) | 75 (28, 137) |  | 4.06 (1.49,8.09) | 1.86 (0.7, 3.44) | -2.28 (-3.03, -1.52) |
| Antigua and Barbuda | 0.03 (0, 0.06) | 0 (0, 0) |  | 0 (0, 0) | 0 (0, 0) |  | 0.1 (0.02,0.22) | 0 (0, 0.01) | -17.03 (-19.64, -14.33) |
| Argentina | 0.03 (0.01, 0.05) | 0.03 (0.01, 0.06) |  | 67 (24, 130) | 233 (88, 426) |  | 0.4 (0.14,0.76) | 0.68 (0.26, 1.23) | 1.78 (1.45, 2.11) |
| Armenia | 0.1 (0.04, 0.16) | 0.13 (0.06, 0.19) |  | 32 (12, 56) | 69 (26, 117) |  | 2.51 (0.96,4.36) | 2.91 (1.09, 4.91) | 1.04 (0.55, 1.54) |
| Australia | 0.01 (0, 0.02) | 0.01 (0, 0.02) |  | 18 (5, 36) | 52 (16, 110) |  | 0.15 (0.05,0.32) | 0.2 (0.06, 0.41) | 0.35 (-0.42, 1.13) |
| Austria | 0.08 (0.04, 0.13) | 0.09 (0.04, 0.14) |  | 67 (30, 112) | 121 (52, 202) |  | 0.79 (0.35,1.31) | 0.97 (0.41, 1.62) | 0.51 (0.04, 0.98) |
| Azerbaijan | 0.08 (0.04, 0.13) | 0.1 (0.04, 0.15) |  | 49 (21, 83) | 59 (25, 118) |  | 1.71 (0.74,2.93) | 1.82 (0.74, 3.66) | -0.44 (-1.17, 0.3) |
| Bahamas | 0.04 (0.01, 0.08) | 0.03 (0.01, 0.07) |  | 0 (0, 0) | 0 (0, 1) |  | 0.2 (0.05,0.44) | 0.18 (0.04, 0.4) | -0.89 (-1.72, -0.05) |
| Bahrain | 0.13 (0.05, 0.22) | 0.17 (0.08, 0.29) |  | 3 (1, 5) | 8 (3, 13) |  | 6.02 (2.12,10.79) | 4.28 (1.95, 7.45) | -1.55 (-2.12, -0.96) |
| Bangladesh | 0.09 (0.04, 0.14) | 0.18 (0.09, 0.26) |  | 994 (383, 2001) | 3406 (1435, 7626) | | 5.85 (2.22,12.07) | 6.5 (2.78, 14.97) | 0.03 (-0.3, 0.37) |
| Barbados | 0.02 (0, 0.05) | 0 (0, 0) |  | 0 (0, 0) | 0 (0, 0) |  | 0.09 (0.01,0.23) | 0 (0, 0.02) | -14.39 (-16.27, -12.47) |
| Belarus | 0.06 (0.02, 0.09) | 0.04 (0.01, 0.06) |  | 131 (39, 242) | 31 (10, 74) |  | 1.54 (0.46,2.87) | 0.26 (0.09, 0.64) | -7.3 (-7.82, -6.78) |
| Belgium | 0.04 (0.01, 0.07) | 0.05 (0.02, 0.08) |  | 62 (23, 114) | 130 (51, 231) |  | 0.61 (0.22,1.11) | 0.79 (0.31, 1.4) | 0.53 (-0.23, 1.31) |
| Belize | 0.06 (0.02, 0.09) | 0.04 (0.02, 0.08) |  | 0 (0, 0) | 1 (0, 1) |  | 0.59 (0.23,1.04) | 0.48 (0.18, 0.93) | -1.12 (-1.87, -0.36) |
| Benin | 0.05 (0.02, 0.09) | 0.11 (0.05, 0.17) |  | 13 (5, 24) | 45 (20, 79) |  | 1.41 (0.54,2.59) | 2.1 (0.91, 3.69) | 1.76 (0.63, 2.91) |
| Bermuda | 0.15 (0.07, 0.23) | 0.08 (0.04, 0.14) |  | 0 (0, 1) | 0 (0, 1) |  | 0.96 (0.42,1.51) | 0.36 (0.15, 0.65) | -3.94 (-4.43, -3.46) |
| Bhutan | 0.1 (0.05, 0.15) | 0.14 (0.07, 0.21) |  | 11 (4, 18) | 34 (16, 53) |  | 11.22 (4.79,19.57) | 15.03 (6.98, 23.88) | 0.99 (0.73, 1.24) |
| Bolivia (Plurinational State of) | 0.03 (0.01, 0.05) | 0.05 (0.02, 0.09) |  | 16 (6, 29) | 62 (24, 114) |  | 1.18 (0.45,2.15) | 1.69 (0.66, 3.14) | 1.6 (-0.05, 3.27) |
| Bosnia and Herzegovina | 0.1 (0.05, 0.16) | 0.08 (0.04, 0.13) |  | 44 (19, 71) | 33 (14, 56) |  | 2.41 (1.06,3.9) | 0.98 (0.42, 1.69) | -3.84 (-4.3, -3.39) |
| Botswana | 0.04 (0.01, 0.07) | 0.06 (0.02, 0.1) |  | 3 (1, 7) | 9 (3, 17) |  | 1.35 (0.47,2.97) | 1.47 (0.5, 2.84) | 1 (0.18, 1.83) |
| Brazil | 0.02 (0.01, 0.04) | 0.05 (0.02, 0.09) |  | 325 (117, 604) | 1682 (699, 2891) | | 0.85 (0.31,1.59) | 1.3 (0.54, 2.23) | 0.22 (-0.65, 1.1) |
| Brunei Darussalam | 0 (0, 0.01) | 0.01 (0, 0.03) |  | 0 (0, 0) | 0 (0, 1) |  | 0.28 (0.02,0.92) | 0.36 (0.02, 1.17) | -1.86 (-3.93, 0.26) |
| Bulgaria | 0.1 (0.05, 0.16) | 0.05 (0.02, 0.09) |  | 116 (52, 184) | 60 (24, 107) |  | 2.05 (0.92,3.26) | 0.66 (0.26, 1.18) | -3.76 (-4.82, -2.68) |
| Burkina Faso | 0.04 (0.02, 0.08) | 0.08 (0.04, 0.13) |  | 14 (5, 27) | 46 (20, 82) |  | 0.76 (0.29,1.45) | 1.14 (0.49, 2.01) | 1.51 (0.31, 2.72) |
| Burundi | 0.03 (0.01, 0.05) | 0.08 (0.03, 0.13) |  | 13 (4, 27) | 39 (15, 72) |  | 1.21 (0.39,2.42) | 2.31 (0.87, 4.27) | 2.68 (1.24, 4.13) |
| Cabo Verde | 0.02 (0, 0.08) | 0.05 (0, 0.12) |  | 1 (0, 3) | 2 (0, 4) |  | 0.6 (0,2.2) | 0.61 (0.03, 1.61) | 2.9 (0.86, 4.97) |
| Cambodia | 0.01 (0, 0.03) | 0.05 (0.02, 0.08) |  | 7 (2, 15) | 63 (24, 113) |  | 0.37 (0.11,0.81) | 1.19 (0.46, 2.14) | 0.04 (-1.44, 1.55) |
| Cameroon | 0.07 (0.03, 0.11) | 0.1 (0.05, 0.16) |  | 33 (14, 59) | 85 (36, 150) |  | 1.82 (0.74,3.23) | 1.75 (0.74, 3) | 4.8 (3.68, 5.93) |
| Canada | 0.04 (0.02, 0.07) | 0.04 (0.02, 0.07) |  | 109 (42, 192) | 297 (107, 543) |  | 0.56 (0.22,0.99) | 0.67 (0.25, 1.23) | 0.13 (-1.28, 1.57) |
| Central African Republic | 0.12 (0.06, 0.19) | 0.12 (0.05, 0.19) |  | 26 (9, 52) | 42 (15, 89) |  | 5.98 (2.12,11.82) | 5.18 (1.86, 11.35) | 0.42 (-0.33, 1.18) |
| Chad | 0.07 (0.03, 0.11) | 0.08 (0.04, 0.13) |  | 23 (9, 43) | 39 (17, 71) |  | 1.78 (0.71,3.3) | 1.89 (0.83, 3.38) | -0.61 (-1.54, 0.33) |
| Chile | 0.04 (0.02, 0.07) | 0.03 (0.01, 0.05) |  | 32 (12, 57) | 60 (19, 119) |  | 0.69 (0.26,1.24) | 0.43 (0.13, 0.85) | 0.94 (-0.09, 1.99) |
| China | 0.09 (0.04, 0.14) | 0.09 (0.04, 0.14) |  | 52447 (22682, 88354) | 39844 (17849, 68638) | | 16.53 (7.24,27.19) | 4.39 (1.97, 7.55) | -2.08 (-2.64, -1.51) |
| Colombia | 0.02 (0.01, 0.03) | 0.02 (0.01, 0.03) |  | 40 (14, 77) | 133 (41, 273) |  | 0.53 (0.19,1.03) | 0.42 (0.13, 0.85) | -4.61 (-5.33, -3.89) |
| Comoros | 0 (0, 0.02) | 0.02 (0, 0.07) |  | 0 (0, 1) | 1 (0, 3) |  | 0.05 (0,0.62) | 0.31 (0, 1.26) | -2.48 (-3.38, -1.58) |
| Congo | 0.02 (0.01, 0.05) | 0.08 (0.03, 0.13) |  | 5 (1, 12) | 22 (7, 43) |  | 1.08 (0.3,2.8) | 2.3 (0.8, 4.59) | 5.84 (3.15, 8.6) |
| Cook Islands | 0.02 (0, 0.07) | 0.02 (0.01, 0.04) |  | 0 (0, 0) | 0 (0, 0) |  | 0.94 (0,3.63) | 0.46 (0.12, 0.99) | 3.71 (1.58, 5.88) |
| Costa Rica | 0.03 (0.01, 0.05) | 0.03 (0.01, 0.05) |  | 5 (1, 9) | 14 (4, 30) |  | 0.56 (0.18,1.16) | 0.48 (0.13, 1.02) | -3.9 (-4.48, -3.32) |
| Croatia | 0.1 (0.05, 0.16) | 0.1 (0.04, 0.15) |  | 35 (16, 57) | 67 (28, 115) |  | 0.95 (0.44,1.57) | 1.09 (0.47, 1.85) | -2.81 (-4.12, -1.49) |
| Cuba | 0.03 (0.01, 0.05) | 0.03 (0.01, 0.05) |  | 18 (6, 34) | 50 (15, 100) |  | 0.35 (0.12,0.67) | 0.46 (0.14, 0.93) | 0.83 (0.59, 1.08) |
| Cyprus | 0.12 (0.05, 0.18) | 0.11 (0.05, 0.17) |  | 12 (5, 21) | 20 (8, 33) |  | 4.44 (1.55,7.66) | 2.26 (0.85, 3.76) | 0.46 (-0.44, 1.37) |
| Czechia | 0.08 (0.03, 0.13) | 0.08 (0.04, 0.13) |  | 67 (30, 115) | 120 (50, 202) |  | 0.78 (0.35,1.35) | 0.89 (0.37, 1.49) | -2.45 (-3.01, -1.89) |
| Côte d'Ivoire | 0.02 (0.01, 0.04) | 0.07 (0.03, 0.11) |  | 7 (3, 15) | 42 (17, 75) |  | 0.54 (0.19,1.1) | 1.11 (0.45, 1.96) | 1.07 (0.6, 1.53) |
| Democratic People's Republic of Korea | 0.08 (0.04, 0.13) | 0.13 (0.06, 0.2) |  | 812 (348, 1496) | 2088 (911, 3429) | | 11 (4.71,20.62) | 11 (4.85, 18.16) | -0.34 (-0.68, -0.01) |
| Democratic Republic of the Congo | 0.06 (0.03, 0.1) | 0.09 (0.04, 0.14) |  | 147 (48, 321) | 549 (198, 1308) |  | 2.73 (0.89,6.2) | 3.76 (1.36, 9.13) | 1.7 (0.29, 3.14) |
| Denmark | 0.03 (0.01, 0.06) | 0.04 (0.02, 0.07) |  | 41 (15, 75) | 90 (31, 167) |  | 0.83 (0.32,1.51) | 1.24 (0.44, 2.31) | 0.25 (-0.56, 1.07) |
| Djibouti | 0.03 (0.01, 0.05) | 0.07 (0.03, 0.12) |  | 0 (0, 1) | 2 (0, 4) |  | 0.55 (0.12,1.3) | 1.03 (0.21, 2.16) | 2.21 (1.41, 3.02) |
| Dominica | 0.02 (0, 0.07) | 0 (0, 0.01) |  | 0 (0, 0) | 0 (0, 0) |  | 0.2 (0,0.58) | 0.01 (0, 0.08) | -10.98 (-13.01, -8.9) |
| Dominican Republic | 0.04 (0.02, 0.07) | 0.02 (0.01, 0.05) |  | 7 (3, 12) | 14 (4, 30) |  | 0.47 (0.18,0.85) | 0.31 (0.09, 0.66) | -1.28 (-1.86, -0.7) |
| Ecuador | 0.01 (0, 0.02) | 0.02 (0.01, 0.04) |  | 6 (2, 13) | 25 (8, 50) |  | 0.31 (0.1,0.63) | 0.39 (0.13, 0.79) | 0.18 (-0.76, 1.14) |
| Egypt | 0.12 (0.06, 0.19) | 0.11 (0.05, 0.18) |  | 345 (154, 577) | 564 (227, 984) |  | 3.1 (1.43,5.21) | 2.97 (1.26, 5.27) | 0.26 (0, 0.52) |
| El Salvador | 0.07 (0.03, 0.12) | 0.06 (0.02, 0.1) |  | 21 (9, 37) | 37 (14, 67) |  | 1.41 (0.62,2.46) | 0.94 (0.35, 1.71) | -2.67 (-3.55, -1.79) |
| Equatorial Guinea | 0.01 (0, 0.03) | 0.06 (0.03, 0.11) |  | 0 (0, 1) | 3 (1, 9) |  | 0.54 (0.15,1.31) | 1.6 (0.48, 4.51) | 3.8 (1.75, 5.9) |
| Eritrea | 0.05 (0.02, 0.08) | 0.07 (0.03, 0.11) |  | 6 (2, 12) | 19 (6, 38) |  | 1.39 (0.47,2.93) | 1.72 (0.56, 3.32) | 1 (0.61, 1.39) |
| Estonia | 0.03 (0.01, 0.06) | 0.03 (0.01, 0.06) |  | 2 (1, 5) | 3 (1, 6) |  | 0.18 (0.06,0.36) | 0.14 (0.04, 0.28) | -1.34 (-1.86, -0.81) |
| Eswatini | 0.02 (0.01, 0.04) | 0.05 (0.02, 0.09) |  | 1 (0, 2) | 3 (1, 7) |  | 0.64 (0.17,1.58) | 1.22 (0.41, 2.48) | 3.1 (1.52, 4.71) |
| Ethiopia | 0.04 (0.02, 0.07) | 0.08 (0.03, 0.12) |  | 104 (34, 204) | 235 (100, 406) |  | 1.38 (0.48,2.7) | 1.47 (0.63, 2.52) | -0.06 (-1.13, 1.03) |
| Fiji | 0 (0, 0.01) | 0.01 (0, 0.02) |  | 0 (0, 0) | 0 (0, 1) |  | 0.1 (0.01,0.3) | 0.13 (0.02, 0.35) | 0.17 (-1.36, 1.72) |
| Finland | 0.02 (0.01, 0.04) | 0.02 (0.01, 0.04) |  | 5 (2, 10) | 10 (3, 20) |  | 0.11 (0.04,0.22) | 0.12 (0.04, 0.25) | -0.84 (-2.13, 0.48) |
| France | 0.08 (0.03, 0.12) | 0.06 (0.03, 0.11) |  | 553 (234, 925) | 497 (202, 877) |  | 0.9 (0.39,1.51) | 0.42 (0.17, 0.75) | -2.71 (-3.12, -2.3) |
| Gabon | 0.01 (0, 0.02) | 0.03 (0.01, 0.06) |  | 0 (0, 2) | 3 (1, 6) |  | 0.19 (0.03,0.59) | 0.66 (0.2, 1.29) | 5.13 (2.56, 7.75) |
| Gambia | 0.03 (0.01, 0.06) | 0.07 (0.03, 0.12) |  | 1 (0, 2) | 6 (2, 13) |  | 0.71 (0.23,1.57) | 1.5 (0.57, 2.92) | 2.79 (1.87, 3.72) |
| Georgia | 0.09 (0.04, 0.14) | 0.1 (0.04, 0.16) |  | 31 (14, 59) | 42 (17, 83) |  | 0.96 (0.41,1.78) | 0.97 (0.41, 1.89) | 1.69 (0.89, 2.51) |
| Germany | 0.05 (0.02, 0.08) | 0.06 (0.02, 0.1) |  | 526 (216, 922) | 1031 (411, 1785) | | 0.59 (0.24,1.03) | 0.79 (0.32, 1.37) | 0.86 (0.53, 1.2) |
| Ghana | 0.04 (0.01, 0.07) | 0.1 (0.04, 0.16) |  | 16 (6, 30) | 81 (34, 137) |  | 0.63 (0.23,1.14) | 1.11 (0.46, 1.9) | 1.97 (0.49, 3.46) |
| Greece | 0.1 (0.05, 0.16) | 0.09 (0.04, 0.14) |  | 106 (47, 172) | 237 (89, 427) |  | 1.31 (0.58,2.16) | 1.29 (0.5, 2.29) | 0.41 (-0.34, 1.16) |
| Greenland | 0.04 (0.02, 0.07) | 0.04 (0.01, 0.06) |  | 0 (0, 1) | 1 (0, 1) |  | 3.46 (1.23,6.35) | 1.91 (0.69, 3.53) | -2.56 (-3.44, -1.66) |
| Grenada | 0.02 (0, 0.06) | 0.01 (0, 0.03) |  | 0 (0, 0) | 0 (0, 0) |  | 0.21 (0.04,0.48) | 0.1 (0.01, 0.31) | -3.9 (-4.95, -2.85) |
| Guam | 0 (0, 0.01) | 0 (0, 0.01) |  | 0 (0, 0) | 0 (0, 0) |  | 0.04 (0,0.35) | 0.01 (0, 0.09) | -6.97 (-10.45, -3.35) |
| Guatemala | 0.04 (0.02, 0.07) | 0.03 (0.01, 0.05) |  | 11 (4, 20) | 27 (10, 52) |  | 0.99 (0.39,1.76) | 0.54 (0.2, 1.04) | -4.04 (-4.95, -3.13) |
| Guinea | 0.03 (0.01, 0.05) | 0.08 (0.03, 0.12) |  | 13 (5, 26) | 43 (18, 81) |  | 0.88 (0.32,1.75) | 1.8 (0.74, 3.33) | 2.87 (1.68, 4.07) |
| Guinea-Bissau | 0.02 (0.01, 0.04) | 0.07 (0.03, 0.11) |  | 1 (0, 3) | 5 (2, 10) |  | 0.7 (0.23,1.48) | 1.63 (0.64, 3.02) | 3.44 (2.22, 4.67) |
| Guyana | 0 (0, 0.01) | 0 (0, 0.01) |  | 0 (0, 0) | 0 (0, 0) |  | 0.02 (0,0.06) | 0.02 (0, 0.09) | -0.48 (-2.32, 1.39) |
| Haiti | 0.06 (0.02, 0.1) | 0.04 (0.02, 0.08) |  | 23 (8, 55) | 34 (11, 80) |  | 1.67 (0.55,4.31) | 1.18 (0.38, 2.86) | -1.89 (-2.58, -1.2) |
| Honduras | 0.04 (0.01, 0.06) | 0.03 (0.01, 0.06) |  | 12 (4, 23) | 46 (14, 96) |  | 1.37 (0.48,2.6) | 1.76 (0.56, 3.59) | -0.1 (-1.11, 0.92) |
| Hungary | 0.09 (0.04, 0.15) | 0.07 (0.03, 0.11) |  | 162 (72, 266) | 173 (72, 297) |  | 1.93 (0.85,3.16) | 1.36 (0.57, 2.32) | -0.81 (-1.25, -0.37) |
| Iceland | 0.02 (0.01, 0.04) | 0.03 (0.01, 0.06) |  | 1 (0, 1) | 2 (1, 3) |  | 0.35 (0.1,0.73) | 0.46 (0.15, 0.92) | 0.86 (0.17, 1.56) |
| India | 0.1 (0.05, 0.15) | 0.19 (0.09, 0.27) |  | 16451 (6994, 28038) | 75428 (34346, 122410) | | 12.04 (5.09,20.52) | 15.72 (7.17, 25.52) | 0.58 (0.3, 0.86) |
| Indonesia | 0.01 (0, 0.02) | 0.05 (0.02, 0.08) |  | 155 (50, 323) | 1083 (461, 1876) | | 0.41 (0.13,0.86) | 1.31 (0.56, 2.25) | 3.72 (2.45, 5) |
| Iran (Islamic Republic of) | 0.12 (0.05, 0.18) | 0.14 (0.07, 0.21) |  | 190 (81, 326) | 689 (319, 1090) |  | 2.29 (0.98,3.99) | 2.32 (1.07, 3.66) | 0.08 (-0.24, 0.41) |
| Iraq | 0.14 (0.07, 0.21) | 0.15 (0.07, 0.23) |  | 37 (16, 63) | 91 (42, 149) |  | 1.04 (0.46,1.79) | 0.99 (0.45, 1.69) | -0.1 (-0.26, 0.07) |
| Ireland | 0.03 (0.01, 0.06) | 0.01 (0, 0.02) |  | 27 (10, 51) | 8 (2, 18) |  | 1.14 (0.41,2.15) | 0.17 (0.04, 0.4) | -6.22 (-7.23, -5.19) |
| Israel | 0.14 (0.06, 0.21) | 0.12 (0.05, 0.18) |  | 57 (26, 89) | 92 (41, 155) |  | 2.36 (1.1,3.7) | 1.22 (0.54, 2.06) | -2.17 (-2.43, -1.9) |
| Italy | 0.14 (0.07, 0.21) | 0.12 (0.06, 0.19) |  | 860 (393, 1335) | 1438 (640, 2246) | | 1.57 (0.72,2.43) | 1.17 (0.53, 1.86) | -1.1 (-1.54, -0.66) |
| Jamaica | 0.07 (0.03, 0.12) | 0.05 (0.02, 0.09) |  | 5 (2, 9) | 6 (2, 11) |  | 0.51 (0.22,0.88) | 0.3 (0.11, 0.56) | -3.11 (-3.98, -2.22) |
| Japan | 0.05 (0.02, 0.08) | 0.08 (0.04, 0.13) |  | 309 (126, 541) | 1059 (436, 1905) | | 0.34 (0.14,0.59) | 0.32 (0.13, 0.56) | -0.48 (-0.98, 0.02) |
| Jordan | 0.13 (0.06, 0.2) | 0.13 (0.06, 0.2) |  | 12 (5, 20) | 19 (9, 33) |  | 2.57 (1.08,4.29) | 0.96 (0.44, 1.66) | -4.16 (-4.76, -3.56) |
| Kazakhstan | 0.09 (0.04, 0.14) | 0.07 (0.03, 0.12) |  | 190 (82, 315) | 225 (90, 388) |  | 2.6 (1.13,4.3) | 2.56 (1.05, 4.41) | -0.22 (-1.02, 0.59) |
| Kenya | 0.01 (0, 0.02) | 0.05 (0.02, 0.08) |  | 8 (2, 20) | 100 (39, 216) |  | 0.24 (0.07,0.58) | 1.1 (0.43, 2.36) | 6.32 (4.16, 8.53) |
| Kiribati | 0.02 (0.01, 0.04) | 0.06 (0.02, 0.09) |  | 0 (0, 0) | 1 (0, 1) |  | 0.79 (0.28,1.55) | 1.94 (0.78, 3.38) | 3.16 (1.9, 4.44) |
| Kuwait | 0.14 (0.07, 0.22) | 0.17 (0.08, 0.25) |  | 2 (1, 3) | 6 (2, 9) |  | 0.87 (0.38,1.41) | 0.75 (0.34, 1.25) | 0.16 (-0.19, 0.5) |
| Kyrgyzstan | 0.13 (0.06, 0.2) | 0.13 (0.06, 0.2) |  | 150 (58, 242) | 80 (36, 131) |  | 7.98 (3.08,12.81) | 3.64 (1.64, 6.02) | -3.1 (-3.66, -2.54) |
| Lao People's Democratic Republic | 0.02 (0.01, 0.04) | 0.06 (0.03, 0.1) |  | 11 (4, 22) | 36 (15, 68) |  | 1.23 (0.41,2.46) | 2.05 (0.85, 3.94) | 1.69 (0.83, 2.56) |
| Latvia | 0.03 (0.01, 0.06) | 0.03 (0.01, 0.06) |  | 6 (2, 12) | 5 (2, 11) |  | 0.26 (0.09,0.47) | 0.16 (0.05, 0.34) | -1.76 (-2.41, -1.1) |
| Lebanon | 0.12 (0.06, 0.19) | 0.1 (0.05, 0.17) |  | 19 (8, 32) | 33 (13, 63) |  | 2.06 (0.87,3.56) | 1.16 (0.48, 2.23) | -1.83 (-2.08, -1.57) |
| Lesotho | 0.03 (0.01, 0.05) | 0.06 (0.03, 0.11) |  | 8 (2, 18) | 20 (7, 40) |  | 1.55 (0.49,3.57) | 3.36 (1.22, 6.45) | 3.79 (2.63, 4.95) |
| Liberia | 0.01 (0, 0.02) | 0.06 (0.02, 0.09) |  | 1 (0, 2) | 7 (3, 15) |  | 0.19 (0.05,0.43) | 0.93 (0.35, 1.83) | 5.92 (3.82, 8.05) |
| Libya | 0.1 (0.05, 0.15) | 0.12 (0.06, 0.18) |  | 10 (4, 17) | 32 (14, 55) |  | 1.29 (0.54,2.21) | 1.49 (0.66, 2.58) | 0.46 (-0.19, 1.12) |
| Lithuania | 0.04 (0.02, 0.07) | 0.02 (0.01, 0.04) |  | 16 (6, 30) | 6 (2, 13) |  | 0.57 (0.2,1.04) | 0.13 (0.04, 0.27) | -5.26 (-5.9, -4.61) |
| Luxembourg | 0.05 (0.02, 0.09) | 0.06 (0.03, 0.1) |  | 2 (1, 4) | 6 (2, 10) |  | 0.7 (0.26,1.27) | 0.81 (0.32, 1.46) | 0.65 (0.13, 1.18) |
| Madagascar | 0 (0, 0.01) | 0.03 (0.01, 0.05) |  | 2 (1, 6) | 38 (13, 78) |  | 0.11 (0.03,0.29) | 0.92 (0.31, 1.89) | 8.22 (5.75, 10.76) |
| Malawi | 0.01 (0, 0.03) | 0.05 (0.02, 0.08) |  | 5 (1, 11) | 24 (9, 47) |  | 0.3 (0.09,0.65) | 0.73 (0.27, 1.4) | 4.31 (2.74, 5.91) |
| Malaysia | 0.02 (0.01, 0.04) | 0.06 (0.02, 0.09) |  | 22 (7, 42) | 106 (45, 190) |  | 0.58 (0.19,1.11) | 1.01 (0.42, 1.82) | 0.86 (-0.26, 2) |
| Maldives | 0.02 (0.01, 0.04) | 0.11 (0.05, 0.17) |  | 1 (0, 2) | 5 (2, 8) |  | 2.69 (0.74,5.74) | 4.57 (2.04, 7.49) | 0.78 (0.06, 1.5) |
| Mali | 0.03 (0.01, 0.05) | 0.07 (0.03, 0.11) |  | 16 (5, 32) | 65 (23, 124) |  | 0.87 (0.3,1.72) | 1.83 (0.68, 3.41) | 2.45 (1.16, 3.77) |
| Malta | 0.16 (0.07, 0.23) | 0.13 (0.06, 0.2) |  | 2 (1, 4) | 4 (1, 6) |  | 1.06 (0.48,1.64) | 0.57 (0.23, 0.97) | -3.06 (-3.7, -2.41) |
| Marshall Islands | 0 (0, 0) | 0 (0, 0) |  | 0 (0, 0) | 0 (0, 0) |  | 0.03 (0,0.28) | 0 (0, 0.06) | -5.85 (-9.48, -2.07) |
| Mauritania | 0.02 (0.01, 0.04) | 0.05 (0.02, 0.09) |  | 3 (1, 5) | 7 (3, 13) |  | 0.54 (0.14,1.1) | 0.82 (0.31, 1.56) | 1.42 (0.15, 2.72) |
| Mauritius | 0 (0, 0.01) | 0 (0, 0) |  | 0 (0, 1) | 0 (0, 0) |  | 0.05 (0,0.16) | 0 (0, 0.01) | -11.75 (-15.1, -8.27) |
| Mexico | 0.16 (0.08, 0.24) | 0.07 (0.03, 0.11) |  | 879 (428, 1317) | 1047 (436, 1756) | | 5.43 (2.65,8.2) | 1.87 (0.78, 3.15) | -3.96 (-4.25, -3.67) |
| Micronesia (Federated States of) | 0 (0, 0.01) | 0 (0, 0) |  | 0 (0, 0) | 0 (0, 0) |  | 0.13 (0,0.81) | 0.02 (0, 0.18) | -6.37 (-10.01, -2.59) |
| Monaco | 0.12 (0.03, 0.22) | 0.1 (0.04, 0.17) |  | 1 (0, 1) | 1 (0, 1) |  | 1.02 (0.25,2.08) | 0.95 (0.37, 1.66) | 0.9 (0.11, 1.69) |
| Mongolia | 0.09 (0.04, 0.14) | 0.07 (0.03, 0.11) |  | 13 (5, 21) | 9 (4, 15) |  | 2.51 (1.09,4.19) | 1 (0.42, 1.71) | -3.53 (-4.16, -2.89) |
| Montenegro | 0.1 (0.05, 0.16) | 0.09 (0.04, 0.14) |  | 2 (1, 3) | 2 (1, 4) |  | 0.48 (0.21,0.86) | 0.39 (0.16, 0.67) | -0.68 (-1.06, -0.29) |
| Morocco | 0.05 (0.02, 0.09) | 0.11 (0.05, 0.17) |  | 49 (14, 99) | 238 (103, 404) |  | 0.89 (0.27,1.79) | 1.89 (0.82, 3.13) | 3.07 (2.7, 3.43) |
| Mozambique | 0.01 (0, 0.02) | 0.04 (0.01, 0.06) |  | 3 (1, 7) | 24 (9, 48) |  | 0.13 (0.04,0.3) | 0.51 (0.18, 1.01) | 5.95 (4.16, 7.78) |
| Myanmar | 0.03 (0.01, 0.06) | 0.06 (0.03, 0.1) |  | 273 (88, 545) | 754 (282, 1356) |  | 2.82 (0.94,5.6) | 3.53 (1.34, 6.4) | 0.34 (-0.2, 0.89) |
| Namibia | 0.06 (0.03, 0.1) | 0.07 (0.03, 0.12) |  | 7 (3, 16) | 13 (5, 28) |  | 2.2 (0.79,4.74) | 1.9 (0.71, 3.9) | -0.08 (-0.86, 0.71) |
| Nauru | 0 (0, 0) | 0 (0, 0) |  | 0 (0, 0) | 0 (0, 0) |  | 0.02 (0,0) | 0.01 (0, 0) | -3.49 (-6.38, -0.5) |
| Nepal | 0.13 (0.06, 0.2) | 0.19 (0.09, 0.28) |  | 841 (336, 1456) | 2916 (1282, 4713) | | 24.59 (10.04,42.44) | 32.24 (14.11, 52.09) | 0.98 (0.82, 1.14) |
| Netherlands | 0.04 (0.02, 0.07) | 0.04 (0.02, 0.08) |  | 76 (30, 140) | 196 (67, 369) |  | 0.59 (0.23,1.08) | 0.9 (0.31, 1.69) | 0.8 (0, 1.61) |
| New Zealand | 0.01 (0, 0.01) | 0.01 (0, 0.03) |  | 3 (1, 7) | 14 (4, 30) |  | 0.13 (0.03,0.32) | 0.3 (0.09, 0.63) | 2.15 (0.89, 3.42) |
| Nicaragua | 0.02 (0.01, 0.04) | 0.03 (0.01, 0.05) |  | 2 (1, 4) | 15 (5, 27) |  | 0.3 (0.1,0.6) | 0.78 (0.25, 1.45) | 2.69 (1.63, 3.76) |
| Niger | 0.06 (0.02, 0.1) | 0.09 (0.04, 0.14) |  | 18 (7, 33) | 65 (27, 120) |  | 1.72 (0.66,3.16) | 2.15 (0.87, 3.93) | 1.23 (0.13, 2.36) |
| Nigeria | 0.08 (0.04, 0.13) | 0.12 (0.05, 0.18) |  | 279 (119, 486) | 618 (266, 1046) |  | 1.44 (0.61,2.5) | 1.78 (0.78, 2.96) | 1.11 (0.35, 1.88) |
| Niue | 0.02 (0.01, 0.04) | 0.02 (0.01, 0.04) |  | 0 (0, 0) | 0 (0, 0) |  | 0.95 (0.22,2.14) | 0.66 (0.17, 1.5) | -3.03 (-3.74, -2.32) |
| North Macedonia | 0.1 (0.05, 0.16) | 0.03 (0.01, 0.06) |  | 21 (10, 35) | 8 (3, 14) |  | 2.69 (1.24,4.51) | 0.55 (0.21, 1.01) | -4.67 (-5.71, -3.62) |
| Northern Mariana Islands | 0.04 (0.01, 0.07) | 0.08 (0.04, 0.14) |  | 0 (0, 0) | 0 (0, 1) |  | 1.54 (0.43,3.21) | 1.86 (0.75, 3.38) | -0.16 (-0.95, 0.62) |
| Norway | 0.04 (0.02, 0.07) | 0.02 (0.01, 0.05) |  | 16 (7, 30) | 29 (9, 58) |  | 0.36 (0.15,0.66) | 0.48 (0.15, 0.96) | 0.52 (-0.62, 1.66) |
| Oman | 0.09 (0.04, 0.15) | 0.11 (0.05, 0.18) |  | 6 (2, 10) | 9 (4, 15) |  | 2.56 (1.07,4.63) | 2.03 (0.84, 3.48) | -0.1 (-0.46, 0.26) |
| Pakistan | 0.12 (0.05, 0.18) | 0.17 (0.08, 0.26) |  | 1313 (561, 2374) | 3050 (1373, 4933) | | 6.28 (2.69,11.45) | 8.1 (3.64, 13.05) | 0.85 (0.65, 1.05) |
| Palau | 0 (0, 0.01) | 0 (0, 0.01) |  | 0 (0, 0) | 0 (0, 0) |  | 0.19 (0,1.26) | 0.07 (0, 0.55) | -3.1 (-6.23, 0.14) |
| Palestine | 0.14 (0.06, 0.2) | 0.12 (0.06, 0.19) |  | 9 (4, 15) | 11 (5, 19) |  | 2.13 (0.88,3.61) | 1.14 (0.5, 2.08) | -2.47 (-2.65, -2.29) |
| Panama | 0.04 (0.02, 0.07) | 0.03 (0.01, 0.06) |  | 4 (2, 8) | 11 (3, 23) |  | 0.6 (0.23,1.11) | 0.44 (0.14, 0.93) | -3.18 (-4.4, -1.94) |
| Papua New Guinea | 0 (0, 0) | 0 (0, 0) |  | 1 (0, 2) | 1 (0, 3) |  | 0.1 (0.02,0.29) | 0.06 (0.01, 0.17) | -3.48 (-6.45, -0.42) |
| Paraguay | 0.03 (0.01, 0.06) | 0.06 (0.02, 0.1) |  | 5 (2, 9) | 17 (6, 31) |  | 0.44 (0.17,0.83) | 0.57 (0.22, 1.06) | 1.03 (-0.05, 2.11) |
| Peru | 0.01 (0, 0.02) | 0.03 (0.01, 0.05) |  | 5 (2, 13) | 43 (16, 85) |  | 0.1 (0.03,0.24) | 0.24 (0.09, 0.48) | 2.71 (1.7, 3.73) |
| Philippines | 0.01 (0, 0.01) | 0.01 (0, 0.01) |  | 21 (6, 46) | 32 (9, 73) |  | 0.21 (0.06,0.45) | 0.1 (0.03, 0.23) | -2.05 (-3.56, -0.51) |
| Poland | 0.06 (0.03, 0.11) | 0.06 (0.03, 0.11) |  | 189 (83, 318) | 242 (101, 424) |  | 0.74 (0.32,1.25) | 0.51 (0.21, 0.88) | -1.3 (-1.66, -0.94) |
| Portugal | 0.04 (0.02, 0.07) | 0.05 (0.02, 0.09) |  | 51 (20, 90) | 136 (53, 251) |  | 0.66 (0.27,1.18) | 0.68 (0.27, 1.25) | 1.13 (-0.03, 2.31) |
| Puerto Rico | 0.02 (0.01, 0.04) | 0 (0, 0.01) |  | 6 (2, 12) | 3 (1, 8) |  | 0.31 (0.1,0.64) | 0.06 (0.01, 0.16) | -7.85 (-9.61, -6.04) |
| Qatar | 0.15 (0.07, 0.23) | 0.19 (0.09, 0.28) |  | 1 (0, 2) | 3 (1, 5) |  | 3.66 (1.58,6.43) | 4.54 (2.13, 7.77) | 1.18 (0.9, 1.47) |
| Republic of Korea | 0.08 (0.03, 0.13) | 0.15 (0.07, 0.22) |  | 164 (55, 297) | 597 (279, 971) |  | 1.4 (0.44,2.57) | 1.15 (0.54, 1.87) | -2.2 (-3.31, -1.07) |
| Republic of Moldova | 0.1 (0.05, 0.16) | 0.04 (0.02, 0.07) |  | 61 (23, 103) | 15 (6, 28) |  | 2.71 (1.05,4.52) | 0.4 (0.15, 0.74) | -6.89 (-7.66, -6.11) |
| Romania | 0.1 (0.05, 0.15) | 0.02 (0.01, 0.04) |  | 432 (152, 717) | 51 (17, 103) |  | 3.3 (1.16,5.5) | 0.21 (0.07, 0.41) | -8.87 (-9.42, -8.32) |
| Russian Federation | 0.07 (0.03, 0.12) | 0.04 (0.01, 0.06) |  | 1345 (497, 2292) | 470 (183, 855) |  | 1.18 (0.44,2.01) | 0.29 (0.11, 0.52) | -4.99 (-5.34, -4.64) |
| Rwanda | 0.03 (0.01, 0.06) | 0.09 (0.04, 0.14) |  | 20 (6, 37) | 62 (26, 120) |  | 1.5 (0.49,2.88) | 2.27 (0.95, 4.43) | 1.78 (-0.02, 3.62) |
| Saint Kitts and Nevis | 0.02 (0, 0.07) | 0 (0, 0) |  | 0 (0, 0) | 0 (0, 0) |  | 0.31 (0.02,0.96) | 0 (0, 0) | -25.11 (-28.56, -21.49) |
| Saint Lucia | 0.02 (0, 0.07) | 0 (0, 0.01) |  | 0 (0, 0) | 0 (0, 0) |  | 0.23 (0,0.92) | 0.01 (0, 0.14) | -11.02 (-12.53, -9.49) |
| Saint Vincent and the Grenadines | 0.02 (0, 0.07) | 0 (0, 0.02) |  | 0 (0, 0) | 0 (0, 0) |  | 0.11 (0,0.39) | 0.01 (0, 0.1) | -9.94 (-11.49, -8.37) |
| Samoa | 0.02 (0, 0.08) | 0.02 (0.01, 0.04) |  | 1 (0, 2) | 1 (0, 1) |  | 1.38 (0,5.12) | 1 (0.27, 2.13) | -2.6 (-3.24, -1.96) |
| San Marino | 0.14 (0.06, 0.23) | 0.11 (0.05, 0.19) |  | 0 (0, 0) | 0 (0, 1) |  | 1.37 (0.54,2.31) | 1.01 (0.4, 1.85) | -0.56 (-1.24, 0.13) |
| Sao Tome and Principe | 0.02 (0, 0.05) | 0.06 (0.02, 0.11) |  | 0 (0, 1) | 1 (0, 2) |  | 0.74 (0.13,2.18) | 2.32 (0.73, 4.68) | 4.38 (3.02, 5.76) |
| Saudi Arabia | 0.11 (0.05, 0.17) | 0.15 (0.07, 0.22) |  | 68 (28, 119) | 110 (48, 180) |  | 3.51 (1.44,6.21) | 2.55 (1.13, 4.19) | -1.18 (-1.41, -0.95) |
| Senegal | 0.03 (0.01, 0.05) | 0.06 (0.03, 0.1) |  | 8 (3, 17) | 37 (16, 66) |  | 0.62 (0.22,1.29) | 1.13 (0.49, 2.03) | 2.44 (1.39, 3.51) |
| Serbia | 0.1 (0.05, 0.16) | 0.03 (0.01, 0.06) |  | 97 (43, 164) | 42 (16, 76) |  | 1.82 (0.82,3.07) | 0.49 (0.18, 0.89) | -4.17 (-4.9, -3.43) |
| Seychelles | 0 (0, 0.03) | 0.02 (0, 0.09) |  | 0 (0, 0) | 0 (0, 1) |  | 0.04 (0,0.52) | 0.28 (0, 1.45) | 6.84 (4.33, 9.41) |
| Sierra Leone | 0.03 (0.01, 0.05) | 0.06 (0.03, 0.1) |  | 5 (2, 10) | 20 (8, 38) |  | 0.62 (0.21,1.25) | 1.3 (0.51, 2.5) | 3.35 (2.12, 4.6) |
| Singapore | 0.03 (0, 0.09) | 0.07 (0.02, 0.14) |  | 6 (0, 19) | 16 (4, 36) |  | 0.65 (0.02,1.96) | 0.4 (0.11, 0.87) | -2.14 (-3.98, -0.27) |
| Slovakia | 0.09 (0.04, 0.14) | 0.08 (0.03, 0.12) |  | 25 (11, 45) | 27 (12, 47) |  | 0.74 (0.32,1.32) | 0.48 (0.22, 0.82) | -0.9 (-1.32, -0.47) |
| Slovenia | 0.11 (0.05, 0.17) | 0.11 (0.05, 0.17) |  | 18 (8, 31) | 23 (10, 40) |  | 1.19 (0.51,2.06) | 0.7 (0.31, 1.18) | -2.9 (-3.29, -2.51) |
| Solomon Islands | 0 (0, 0) | 0 (0, 0) |  | 0 (0, 0) | 0 (0, 0) |  | 0.05 (0,0.21) | 0.03 (0, 0.14) | -2.47 (-5.35, 0.5) |
| Somalia | 0.01 (0, 0.01) | 0.04 (0.02, 0.07) |  | 2 (1, 6) | 32 (10, 74) |  | 0.23 (0.06,0.57) | 1.14 (0.37, 2.58) | 5.3 (4.02, 6.6) |
| South Africa | 0.02 (0.01, 0.04) | 0.05 (0.02, 0.08) |  | 67 (25, 129) | 253 (106, 438) |  | 0.63 (0.23,1.19) | 1.1 (0.46, 1.9) | 2.05 (1.21, 2.9) |
| South Sudan | 0.08 (0.04, 0.13) | 0.07 (0.03, 0.11) |  | 15 (5, 29) | 12 (5, 25) |  | 1.62 (0.56,3.11) | 0.92 (0.35, 1.83) | -1.6 (-2.37, -0.82) |
| Spain | 0.05 (0.02, 0.09) | 0.09 (0.04, 0.15) |  | 321 (131, 546) | 1080 (436, 1854) | | 0.97 (0.39,1.65) | 1.24 (0.52, 2.09) | 0.7 (0.27, 1.12) |
| Sri Lanka | 0.01 (0, 0.02) | 0.06 (0.02, 0.09) |  | 6 (2, 15) | 103 (35, 194) |  | 0.16 (0.04,0.37) | 0.85 (0.28, 1.6) | 5.31 (4.38, 6.24) |
| Sudan | 0.05 (0.02, 0.08) | 0.08 (0.04, 0.13) |  | 44 (15, 99) | 112 (44, 232) |  | 1.2 (0.41,2.73) | 1.62 (0.67, 3.36) | 1.27 (0.66, 1.88) |
| Suriname | 0 (0, 0) | 0 (0, 0.01) |  | 0 (0, 0) | 0 (0, 0) |  | 0.03 (0.01,0.07) | 0.04 (0.01, 0.1) | -0.23 (-2.09, 1.67) |
| Sweden | 0.03 (0.01, 0.06) | 0.03 (0.01, 0.05) |  | 24 (9, 45) | 58 (19, 117) |  | 0.25 (0.09,0.45) | 0.41 (0.13, 0.82) | 1.42 (0.37, 2.48) |
| Switzerland | 0.1 (0.05, 0.16) | 0.09 (0.04, 0.15) |  | 64 (29, 105) | 107 (48, 181) |  | 0.91 (0.41,1.47) | 0.87 (0.38, 1.47) | 0.18 (-0.13, 0.49) |
| Syrian Arab Republic | 0.12 (0.06, 0.19) | 0.12 (0.05, 0.18) |  | 51 (20, 94) | 85 (34, 157) |  | 2.57 (1,4.91) | 2.33 (0.95, 4.34) | -0.45 (-0.68, -0.22) |
| Taiwan (Province of China) | 0.06 (0.02, 0.1) | 0.06 (0.03, 0.11) |  | 61 (23, 107) | 163 (64, 311) |  | 1.16 (0.43,2.04) | 0.71 (0.28, 1.35) | -2.01 (-2.83, -1.18) |
| Tajikistan | 0.15 (0.07, 0.22) | 0.12 (0.05, 0.18) |  | 79 (36, 132) | 60 (25, 112) |  | 5.07 (2.32,8.43) | 3.23 (1.33, 6.59) | -1.43 (-1.73, -1.12) |
| Thailand | 0.02 (0.01, 0.03) | 0.08 (0.04, 0.13) |  | 99 (33, 203) | 491 (204, 863) |  | 0.64 (0.21,1.31) | 0.88 (0.37, 1.54) | 0.59 (-0.2, 1.38) |
| Timor-Leste | 0.01 (0, 0.02) | 0.03 (0.01, 0.05) |  | 1 (0, 1) | 3 (1, 7) |  | 0.6 (0.16,1.44) | 0.99 (0.31, 2.27) | 0.92 (-0.64, 2.5) |
| Togo | 0.05 (0.02, 0.09) | 0.11 (0.05, 0.17) |  | 7 (3, 13) | 34 (14, 62) |  | 1.38 (0.54,2.55) | 2.1 (0.88, 3.79) | 1.87 (0.53, 3.23) |
| Tokelau | 0.02 (0.01, 0.04) | 0.02 (0.01, 0.04) |  | 0 (0, 0) | 0 (0, 0) |  | 1.48 (0.37,3.41) | 0.9 (0.21, 1.95) | -3.21 (-3.9, -2.52) |
| Tonga | 0.02 (0, 0.04) | 0.02 (0, 0.05) |  | 0 (0, 0) | 0 (0, 0) |  | 0.53 (0.12,1.27) | 0.41 (0.08, 1.02) | -2.31 (-2.98, -1.63) |
| Trinidad and Tobago | 0.01 (0, 0.04) | 0.01 (0, 0.02) |  | 0 (0, 1) | 0 (0, 1) |  | 0.11 (0.01,0.31) | 0.03 (0, 0.1) | -5.87 (-7.01, -4.72) |
| Tunisia | 0.12 (0.06, 0.19) | 0.12 (0.05, 0.18) |  | 31 (13, 56) | 77 (33, 138) |  | 1.71 (0.75,3.13) | 1.33 (0.58, 2.4) | -1.2 (-1.53, -0.88) |
| Turkey | 0.11 (0.05, 0.16) | 0.1 (0.05, 0.16) |  | 554 (235, 925) | 1047 (387, 1836) | | 3.42 (1.46,5.72) | 2.34 (0.86, 4.1) | -0.77 (-1.41, -0.14) |
| Turkmenistan | 0.11 (0.05, 0.17) | 0.09 (0.04, 0.14) |  | 30 (12, 51) | 14 (5, 40) |  | 3.14 (1.27,5.3) | 0.73 (0.25, 2.11) | -5.69 (-6.39, -4.97) |
| Tuvalu | 0 (0, 0.01) | 0 (0, 0.01) |  | 0 (0, 0) | 0 (0, 0) |  | 0.18 (0,0.75) | 0.24 (0, 0.81) | 0.25 (-1.44, 1.98) |
| Uganda | 0.04 (0.02, 0.07) | 0.07 (0.03, 0.11) |  | 26 (8, 55) | 77 (30, 155) |  | 1 (0.32,2.08) | 1.22 (0.49, 2.45) | 0.75 (-1.26, 2.8) |
| Ukraine | 0.09 (0.04, 0.15) | 0.05 (0.02, 0.08) |  | 1120 (411, 1885) | 190 (69, 470) |  | 2.45 (0.9,4.13) | 0.36 (0.13, 0.9) | -7.52 (-7.84, -7.2) |
| United Arab Emirates | 0.14 (0.07, 0.21) | 0.13 (0.06, 0.2) |  | 4 (2, 8) | 14 (6, 26) |  | 5.05 (2.03,10.17) | 2.75 (1.24, 4.95) | -1.49 (-2.71, -0.27) |
| United Kingdom | 0.02 (0.01, 0.05) | 0.01 (0, 0.03) |  | 308 (111, 570) | 292 (85, 624) |  | 0.52 (0.19,0.97) | 0.35 (0.1, 0.75) | -0.14 (-1.1, 0.83) |
| United Republic of Tanzania | 0.01 (0, 0.02) | 0.04 (0.02, 0.07) |  | 8 (3, 17) | 69 (26, 131) |  | 0.18 (0.06,0.38) | 0.65 (0.24, 1.21) | 5.4 (3.57, 7.26) |
| United States of America | 0.1 (0.05, 0.15) | 0.07 (0.03, 0.11) |  | 3864 (1790, 6208) | 6472 (2540, 11076) | | 1.91 (0.88,3.07) | 1.87 (0.74, 3.2) | -0.18 (-0.82, 0.48) |
| United States Virgin Islands | 0.02 (0.01, 0.04) | 0 (0, 0) |  | 0 (0, 0) | 0 (0, 0) |  | 0.18 (0.05,0.39) | 0.01 (0, 0.04) | -11.14 (-13.24, -8.99) |
| Uruguay | 0.01 (0, 0.03) | 0.02 (0.01, 0.04) |  | 4 (1, 8) | 13 (3, 28) |  | 0.15 (0.04,0.36) | 0.34 (0.09, 0.72) | 2.52 (1.92, 3.12) |
| Uzbekistan | 0.14 (0.06, 0.21) | 0.11 (0.05, 0.17) |  | 195 (81, 327) | 112 (48, 220) |  | 3.15 (1.31,5.26) | 1.78 (0.74, 3.94) | -2.78 (-3.32, -2.23) |
| Vanuatu | 0 (0, 0.01) | 0.01 (0, 0.02) |  | 0 (0, 0) | 0 (0, 1) |  | 0.3 (0.04,0.89) | 0.31 (0.04, 0.91) | -0.62 (-2.19, 0.99) |
| Venezuela (Bolivarian Republic of) | 0.03 (0.01, 0.06) | 0.03 (0.01, 0.05) |  | 22 (8, 41) | 79 (30, 143) |  | 0.51 (0.19,0.94) | 0.51 (0.19, 0.93) | -1.35 (-2.25, -0.45) |
| Viet Nam | 0.02 (0.01, 0.04) | 0.05 (0.02, 0.08) |  | 158 (39, 338) | 479 (107, 946) |  | 0.74 (0.18,1.57) | 1.02 (0.23, 2.01) | 1.66 (0.92, 2.41) |
| Yemen | 0.05 (0.02, 0.09) | 0.09 (0.04, 0.15) |  | 35 (13, 78) | 126 (51, 226) |  | 1.74 (0.63,3.82) | 2.42 (0.98, 4.33) | 1.37 (0.94, 1.8) |
| Zambia | 0.04 (0.02, 0.07) | 0.07 (0.03, 0.11) |  | 11 (4, 21) | 34 (13, 62) |  | 1.06 (0.4,1.99) | 1.24 (0.48, 2.25) | 1.24 (0.24, 2.25) |
| Zimbabwe | 0.03 (0.01, 0.05) | 0.06 (0.03, 0.1) |  | 8 (3, 17) | 31 (10, 62) |  | 0.52 (0.17,1.05) | 1.06 (0.34, 2.09) | 4.7 (3.1, 6.33) |

# Table S3. Number, percentage, and age-standardized rate of DALYs from COPD attributable to ozone pollution by country, 1990-2019

(A) Men

| Country | Percentage (%) | |  | Death number | |  | Age-standardized DALY rate | | |
| --- | --- | --- | --- | --- | --- | --- | --- | --- | --- |
|  | 1990  (uncertainty interval) | 2019  (uncertainty interval) |  | 1990  (uncertainty interval) | 2019  (uncertainty interval) |  | 1990  (uncertainty interval)  (per 100,000 person-years) | 2019  (uncertainty interval)  (per 100,000 person-years) | Annual percentage change, 1990-2019 (confidence interval) (%) |
| Afghanistan | 0.11 (0.05, 0.17) | 0.09 (0.04, 0.13) |  | 5149 (2045, 9343) | 5812 (2412, 9805) | | 144.19 (57.02,259.19) | 98.78 (41.46, 166.48) | -1.18 (-1.51, -0.84) |
| Albania | 0.09 (0.04, 0.14) | 0.03 (0.01, 0.05) |  | 918 (410, 1457) | 233 (86, 435) |  | 117.64 (52.49,185.52) | 11.8 (4.37, 21.75) | -7.18 (-7.61, -6.76) |
| Algeria | 0.07 (0.03, 0.11) | 0.08 (0.04, 0.12) |  | 2723 (1117, 4700) | 6673 (2999, 11205) | | 53.77 (22.5,93.03) | 43.37 (19.41, 73.69) | -1.09 (-1.39, -0.8) |
| American Samoa | 0.02 (0, 0.04) | 0.02 (0, 0.04) |  | 3 (1, 7) | 4 (1, 9) |  | 32.88 (8.42,71.76) | 19.91 (5.05, 42.86) | -3.34 (-4.04, -2.64) |
| Andorra | 0.05 (0.02, 0.1) | 0.06 (0.03, 0.11) |  | 13 (5, 26) | 29 (12, 51) |  | 54.13 (18.82,102.59) | 42.23 (17.68, 73.26) | -0.79 (-1.14, -0.43) |
| Angola | 0.07 (0.03, 0.12) | 0.04 (0.02, 0.07) |  | 1727 (735, 3031) | 1735 (705, 2987) | | 99.83 (42.44,168.95) | 40.27 (17.08, 68.03) | -2.67 (-3.37, -1.96) |
| Antigua and Barbuda | 0.02 (0, 0.05) | 0 (0, 0) |  | 1 (0, 3) | 0 (0, 0) |  | 5.03 (0.9,12) | 0.02 (0, 0.3) | -18.3 (-20.82, -15.69) |
| Argentina | 0.02 (0.01, 0.04) | 0.03 (0.01, 0.05) |  | 2637 (955, 5014) | 5057 (1928, 9181) | | 19.08 (6.94,36.1) | 21.91 (8.33, 39.78) | 0.26 (-0.09, 0.6) |
| Armenia | 0.08 (0.04, 0.13) | 0.1 (0.05, 0.16) |  | 1253 (565, 2050) | 1378 (607, 2243) | | 120.22 (53.99,195.89) | 82.08 (36.17, 134.07) | -1.66 (-2.03, -1.3) |
| Australia | 0.01 (0, 0.01) | 0.01 (0, 0.02) |  | 619 (189, 1279) | 899 (298, 1821) |  | 7.37 (2.25,15.2) | 4.47 (1.48, 9.09) | -2.3 (-3.17, -1.42) |
| Austria | 0.06 (0.03, 0.09) | 0.06 (0.03, 0.1) |  | 1613 (743, 2609) | 2450 (1111, 3947) | | 36.68 (16.85,59.37) | 30.79 (13.94, 49.53) | -0.88 (-1.29, -0.47) |
| Azerbaijan | 0.07 (0.03, 0.11) | 0.07 (0.03, 0.12) |  | 1479 (658, 2412) | 1858 (782, 3167) | | 73.83 (32.74,119.73) | 51.48 (21.45, 87.52) | -2.07 (-3.01, -1.11) |
| Bahamas | 0.03 (0.01, 0.06) | 0.03 (0.01, 0.06) |  | 7 (2, 14) | 14 (3, 34) |  | 11.05 (2.69,24) | 8.88 (1.98, 20.74) | -1.35 (-2.08, -0.61) |
| Bahrain | 0.09 (0.03, 0.16) | 0.1 (0.04, 0.16) |  | 93 (34, 165) | 231 (103, 397) |  | 145.18 (53.41,251.13) | 75.11 (33.61, 128.05) | -2.63 (-3.3, -1.97) |
| Bangladesh | 0.08 (0.03, 0.12) | 0.14 (0.07, 0.2) |  | 58903 (24717, 97685) | 117981 (56134, 202615) | | 241 (101.24,400.65) | 181.43 (86.09, 316.24) | -1.37 (-1.67, -1.06) |
| Barbados | 0.02 (0, 0.04) | 0 (0, 0) |  | 5 (1, 11) | 0 (0, 1) |  | 3.55 (0.43,8.87) | 0.05 (0, 0.54) | -15.66 (-17.5, -13.78) |
| Belarus | 0.05 (0.02, 0.08) | 0.03 (0.01, 0.05) |  | 3628 (1407, 6224) | 981 (373, 1959) |  | 80.49 (31.11,137.98) | 16.98 (6.53, 33.57) | -6.67 (-7.39, -5.95) |
| Belgium | 0.03 (0.01, 0.05) | 0.03 (0.01, 0.06) |  | 2256 (821, 4063) | 2670 (1081, 4806) | | 36.19 (13.18,65.11) | 25.59 (10.33, 45.94) | -1.97 (-2.71, -1.23) |
| Belize | 0.04 (0.02, 0.07) | 0.03 (0.01, 0.07) |  | 9 (3, 15) | 29 (11, 57) |  | 19.45 (7.72,34.39) | 22 (8.26, 42.92) | -0.5 (-1.49, 0.51) |
| Benin | 0.04 (0.02, 0.07) | 0.07 (0.03, 0.12) |  | 474 (190, 886) | 1506 (663, 2549) | | 51.49 (20.91,95.66) | 71.82 (31.04, 119.5) | 1.52 (0.43, 2.62) |
| Bermuda | 0.12 (0.06, 0.18) | 0.06 (0.03, 0.1) |  | 12 (6, 19) | 10 (4, 18) |  | 48.15 (22.68,74.94) | 18.26 (7.85, 31.5) | -3.32 (-3.62, -3.02) |
| Bhutan | 0.08 (0.04, 0.12) | 0.11 (0.05, 0.17) |  | 228 (86, 436) | 556 (243, 1073) |  | 225.03 (87.62,414.38) | 214.31 (95.13, 410.4) | -0.04 (-0.28, 0.19) |
| Bolivia (Plurinational State of) | 0.02 (0.01, 0.04) | 0.04 (0.02, 0.07) |  | 322 (122, 626) | 1011 (400, 1794) | | 24.96 (9.45,48.17) | 28.24 (11.16, 50.73) | 0.74 (-0.85, 2.35) |
| Bosnia and Herzegovina | 0.08 (0.04, 0.12) | 0.06 (0.03, 0.1) |  | 1229 (541, 1921) | 944 (406, 1570) |  | 78.57 (35.1,123.5) | 36.75 (16.02, 61.06) | -3.25 (-3.69, -2.81) |
| Botswana | 0.03 (0.01, 0.05) | 0.04 (0.02, 0.07) |  | 137 (48, 283) | 338 (126, 629) |  | 60.11 (21.38,121.31) | 65.61 (24.69, 120.64) | 0.5 (-0.38, 1.37) |
| Brazil | 0.02 (0.01, 0.04) | 0.05 (0.02, 0.08) |  | 10546 (3932, 19243) | 35296 (14699, 59886) | | 27.61 (10.27,50.56) | 34.85 (14.56, 58.88) | -0.5 (-1.3, 0.31) |
| Brunei Darussalam | 0 (0, 0.01) | 0.01 (0, 0.02) |  | 2 (0, 7) | 7 (0, 22) |  | 8.03 (0.55,25.93) | 9.37 (0.43, 30.8) | -1.21 (-3.03, 0.66) |
| Bulgaria | 0.08 (0.04, 0.13) | 0.04 (0.02, 0.07) |  | 4745 (2142, 7469) | 1709 (703, 2926) | | 85.57 (38.84,134.8) | 28.24 (11.67, 48.29) | -3.47 (-4.55, -2.37) |
| Burkina Faso | 0.03 (0.01, 0.06) | 0.05 (0.02, 0.09) |  | 466 (180, 892) | 1492 (655, 2578) | | 24.89 (9.7,46.87) | 39.64 (17.47, 66.52) | 1.74 (0.49, 3.02) |
| Burundi | 0.02 (0.01, 0.04) | 0.06 (0.03, 0.1) |  | 528 (188, 1062) | 1732 (663, 3279) | | 52 (18.52,102.59) | 81.69 (31.51, 153.19) | 1.93 (0.45, 3.44) |
| Cabo Verde | 0.02 (0, 0.06) | 0.03 (0, 0.08) |  | 21 (0, 73) | 33 (2, 87) |  | 21.02 (0,73.94) | 19.33 (0.96, 52.26) | -1.25 (-2.74, 0.26) |
| Cambodia | 0.01 (0, 0.02) | 0.03 (0.01, 0.06) |  | 276 (84, 597) | 1975 (814, 3561) | | 16.04 (4.91,34.66) | 46.62 (19.37, 82.46) | 4.59 (3.45, 5.75) |
| Cameroon | 0.05 (0.02, 0.08) | 0.06 (0.03, 0.1) |  | 1199 (491, 2110) | 3320 (1303, 5854) | | 61.56 (25.32,109.1) | 62.2 (25.12, 106.86) | 0.24 (-1.06, 1.56) |
| Canada | 0.03 (0.01, 0.05) | 0.03 (0.01, 0.05) |  | 3340 (1305, 5772) | 4692 (1815, 8329) | | 24.46 (9.56,42.24) | 14.45 (5.61, 25.65) | -2.08 (-2.58, -1.57) |
| Central African Republic | 0.1 (0.05, 0.16) | 0.09 (0.04, 0.15) |  | 1022 (425, 1773) | 1448 (576, 2599) | | 202.67 (83.53,357.03) | 160.96 (65.44, 286.91) | -0.99 (-1.93, -0.04) |
| Chad | 0.05 (0.02, 0.08) | 0.06 (0.03, 0.09) |  | 762 (303, 1528) | 1716 (668, 2955) | | 57.71 (23.09,114.41) | 60.91 (24.08, 103.22) | 0.86 (-0.07, 1.79) |
| Chile | 0.03 (0.01, 0.06) | 0.02 (0.01, 0.04) |  | 854 (333, 1502) | 952 (331, 1834) |  | 20.42 (7.95,35.88) | 9.22 (3.2, 17.74) | -3.04 (-3.57, -2.49) |
| China | 0.08 (0.04, 0.12) | 0.08 (0.03, 0.12) |  | 1064683 (468202, 1750136) | 831542 (364682, 1347313) | | 333.91 (145.19,542.13) | 107.67 (47.78, 172.57) | -3.74 (-4.4, -3.06) |
| Colombia | 0.01 (0.01, 0.03) | 0.01 (0, 0.03) |  | 1001 (355, 1904) | 2020 (665, 4010) | | 12.93 (4.55,24.45) | 8.49 (2.79, 16.84) | -3.25 (-4.2, -2.29) |
| Comoros | 0 (0, 0.02) | 0.01 (0, 0.05) |  | 2 (0, 25) | 23 (0, 97) |  | 2.45 (0,25.37) | 11.29 (0, 47.08) | 4.95 (2.2, 7.76) |
| Congo | 0.02 (0.01, 0.04) | 0.05 (0.02, 0.09) |  | 145 (44, 306) | 563 (193, 1034) |  | 33.23 (10.29,69.88) | 52.2 (18.15, 94.66) | 2.22 (0.17, 4.31) |
| Cook Islands | 0.02 (0, 0.06) | 0.01 (0, 0.03) |  | 1 (0, 5) | 1 (0, 2) |  | 20.66 (0,75.45) | 9.37 (2.37, 20.02) | -4.36 (-5, -3.72) |
| Costa Rica | 0.02 (0.01, 0.04) | 0.02 (0.01, 0.04) |  | 83 (29, 169) | 216 (64, 469) |  | 10.52 (3.63,21.46) | 9.59 (2.83, 20.85) | -2.56 (-3.8, -1.29) |
| Croatia | 0.07 (0.03, 0.12) | 0.07 (0.03, 0.11) |  | 1291 (583, 2068) | 1606 (718, 2645) | | 52.79 (24.01,85.03) | 43.08 (19.17, 70.99) | -0.66 (-0.86, -0.45) |
| Cuba | 0.02 (0.01, 0.04) | 0.02 (0.01, 0.04) |  | 431 (155, 818) | 1048 (339, 2066) | | 8.6 (3.09,16.33) | 11.71 (3.79, 23.1) | 0.3 (-0.64, 1.24) |
| Cyprus | 0.09 (0.04, 0.14) | 0.08 (0.03, 0.12) |  | 257 (112, 418) | 404 (183, 657) |  | 78.74 (35.11,128.55) | 47.52 (21.26, 77.4) | -2.02 (-2.33, -1.7) |
| Czechia | 0.06 (0.03, 0.1) | 0.06 (0.03, 0.1) |  | 2796 (1231, 4546) | 3345 (1456, 5603) | | 50.8 (22.3,82.6) | 36.26 (15.73, 60.71) | -0.26 (-0.83, 0.32) |
| Côte d'Ivoire | 0.02 (0.01, 0.03) | 0.05 (0.02, 0.07) |  | 441 (154, 895) | 2143 (859, 3796) | | 23.11 (8.11,46.27) | 42.9 (17.19, 75.62) | 2.07 (0.14, 4.03) |
| Democratic People's Republic of Korea | 0.07 (0.03, 0.11) | 0.11 (0.05, 0.17) |  | 12572 (5039, 21465) | 34471 (14649, 54975) | | 260.79 (107.94,440.42) | 308.61 (131.8, 486.27) | 0.37 (0.05, 0.69) |
| Democratic Republic of the Congo | 0.05 (0.02, 0.08) | 0.06 (0.03, 0.1) |  | 4583 (1649, 8243) | 11677 (4398, 21349) | | 69.52 (25.75,124.13) | 83.64 (31.58, 148.62) | 1.12 (-0.28, 2.54) |
| Denmark | 0.03 (0.01, 0.05) | 0.03 (0.01, 0.05) |  | 1000 (373, 1824) | 1279 (475, 2287) | | 28.26 (10.54,51.51) | 23.46 (8.72, 41.96) | -1.75 (-2.37, -1.12) |
| Djibouti | 0.02 (0.01, 0.04) | 0.05 (0.02, 0.08) |  | 13 (4, 30) | 102 (36, 215) |  | 22.18 (6.29,49.88) | 37.46 (13.36, 76.06) | 1.72 (0.98, 2.47) |
| Dominica | 0.02 (0, 0.06) | 0 (0, 0.01) |  | 3 (0, 9) | 0 (0, 2) |  | 9.8 (0.23,29.71) | 0.4 (0, 3.58) | -11.44 (-13.49, -9.35) |
| Dominican Republic | 0.03 (0.01, 0.06) | 0.02 (0.01, 0.04) |  | 178 (71, 323) | 367 (108, 776) |  | 10.45 (4.2,18.96) | 8.5 (2.5, 17.92) | -0.66 (-1.38, 0.05) |
| Ecuador | 0.01 (0, 0.02) | 0.02 (0.01, 0.03) |  | 132 (41, 252) | 449 (150, 900) |  | 5.54 (1.71,10.67) | 7.25 (2.4, 14.67) | 0.12 (-0.8, 1.05) |
| Egypt | 0.09 (0.04, 0.14) | 0.07 (0.03, 0.12) |  | 11752 (5140, 19417) | 23726 (8976, 43010) | | 83.67 (36.46,137.72) | 69.58 (26.63, 125.51) | -0.47 (-0.72, -0.23) |
| El Salvador | 0.06 (0.03, 0.09) | 0.04 (0.02, 0.08) |  | 381 (165, 633) | 430 (164, 768) |  | 29.02 (12.58,48.24) | 16.67 (6.34, 29.94) | -3.34 (-4.16, -2.52) |
| Equatorial Guinea | 0.01 (0, 0.02) | 0.04 (0.02, 0.07) |  | 16 (4, 37) | 67 (23, 128) |  | 19.89 (5.36,45.57) | 39.01 (13.66, 72.1) | 2.04 (-0.21, 4.34) |
| Eritrea | 0.04 (0.02, 0.06) | 0.05 (0.02, 0.08) |  | 262 (93, 509) | 626 (259, 1148) |  | 70.57 (25.58,136.32) | 61.86 (25.09, 110.6) | -0.35 (-0.71, 0) |
| Estonia | 0.02 (0.01, 0.05) | 0.02 (0.01, 0.04) |  | 100 (34, 192) | 73 (23, 150) |  | 13.59 (4.69,25.94) | 7.4 (2.33, 15.23) | -2.34 (-2.97, -1.7) |
| Eswatini | 0.01 (0, 0.03) | 0.04 (0.01, 0.06) |  | 36 (11, 74) | 147 (54, 272) |  | 32.66 (10.06,65.59) | 70.35 (25.9, 129.48) | 3.53 (1.97, 5.1) |
| Ethiopia | 0.04 (0.01, 0.06) | 0.05 (0.02, 0.09) |  | 5486 (2157, 10468) | 8929 (3722, 15366) | | 55.84 (22.17,105.52) | 46.78 (19.7, 80.88) | -0.81 (-1.97, 0.36) |
| Fiji | 0 (0, 0.01) | 0.01 (0, 0.02) |  | 6 (1, 19) | 16 (2, 45) |  | 4.07 (0.4,11.72) | 5.56 (0.84, 15.27) | 0.15 (-1.29, 1.61) |
| Finland | 0.01 (0, 0.03) | 0.01 (0, 0.02) |  | 264 (89, 492) | 297 (99, 598) |  | 9.8 (3.32,18.3) | 5.23 (1.73, 10.54) | -3.41 (-4.66, -2.14) |
| France | 0.06 (0.03, 0.1) | 0.05 (0.02, 0.08) |  | 11814 (5476, 19252) | 8416 (3471, 14199) | | 36.25 (16.77,59.46) | 13.6 (5.7, 22.98) | -3.42 (-3.81, -3.02) |
| Gabon | 0 (0, 0.01) | 0.02 (0.01, 0.04) |  | 14 (3, 44) | 86 (29, 172) |  | 6.17 (1.23,18.81) | 20.38 (6.93, 40.36) | 4.84 (2.2, 7.54) |
| Gambia | 0.02 (0.01, 0.04) | 0.05 (0.02, 0.08) |  | 41 (12, 89) | 211 (81, 391) |  | 25.03 (7.72,53.05) | 48.92 (19.44, 90.26) | 2.52 (1.62, 3.43) |
| Georgia | 0.07 (0.03, 0.11) | 0.08 (0.03, 0.12) |  | 731 (319, 1221) | 1094 (467, 1828) | | 33.33 (14.67,55.37) | 46.91 (20.06, 78.52) | 3.22 (2.35, 4.1) |
| Germany | 0.03 (0.01, 0.06) | 0.04 (0.02, 0.06) |  | 15250 (6271, 26642) | 20346 (8435, 34987) | | 32.6 (13.41,56.98) | 23.48 (9.83, 40.37) | -1.51 (-1.88, -1.13) |
| Ghana | 0.03 (0.01, 0.05) | 0.07 (0.03, 0.11) |  | 815 (279, 1615) | 5462 (1967, 9454) | | 30.58 (10.38,59.62) | 83.08 (30.14, 142.03) | 4.35 (2.72, 6.01) |
| Greece | 0.06 (0.03, 0.1) | 0.05 (0.02, 0.08) |  | 2127 (942, 3301) | 3306 (1404, 5366) | | 31.46 (14.06,48.9) | 27.43 (11.54, 43.92) | -0.01 (-0.54, 0.52) |
| Greenland | 0.03 (0.01, 0.05) | 0.03 (0.01, 0.05) |  | 6 (2, 11) | 8 (3, 15) |  | 46.36 (17.43,83.94) | 25.13 (9.72, 45.76) | -1.86 (-2.62, -1.1) |
| Grenada | 0.02 (0, 0.05) | 0.01 (0, 0.03) |  | 3 (1, 7) | 2 (0, 6) |  | 9.96 (2.05,23.49) | 3.74 (0.37, 11.47) | -4.8 (-6.11, -3.47) |
| Guam | 0 (0, 0.01) | 0 (0, 0.01) |  | 0 (0, 4) | 0 (0, 3) |  | 1.38 (0,13.08) | 0.28 (0, 3.48) | -6.29 (-9.88, -2.56) |
| Guatemala | 0.03 (0.01, 0.05) | 0.02 (0.01, 0.04) |  | 303 (121, 534) | 440 (164, 839) |  | 19.14 (7.51,33.61) | 9.98 (3.78, 18.99) | -4.16 (-5.07, -3.24) |
| Guinea | 0.02 (0.01, 0.04) | 0.05 (0.02, 0.09) |  | 361 (125, 753) | 1466 (566, 2523) | | 23.12 (8.11,47.85) | 55.75 (21.29, 95.57) | 3.68 (2.52, 4.85) |
| Guinea-Bissau | 0.02 (0.01, 0.04) | 0.05 (0.02, 0.08) |  | 66 (20, 137) | 212 (80, 396) |  | 34.94 (10.41,71.77) | 67.19 (26.11, 122.52) | 2.79 (1.59, 4.01) |
| Guyana | 0 (0, 0.01) | 0 (0, 0.01) |  | 2 (0, 5) | 3 (0, 12) |  | 0.94 (0.14,2.86) | 1.07 (0.07, 4.29) | -0.22 (-2.04, 1.63) |
| Haiti | 0.05 (0.02, 0.08) | 0.04 (0.01, 0.07) |  | 886 (309, 1663) | 1151 (366, 2363) | | 63.04 (22.12,117.65) | 38.87 (12.45, 77.99) | -2.34 (-2.96, -1.71) |
| Honduras | 0.03 (0.01, 0.05) | 0.03 (0.01, 0.05) |  | 303 (115, 547) | 938 (335, 1816) |  | 31.62 (12.05,56.92) | 35.9 (12.76, 69.05) | -0.25 (-1.17, 0.68) |
| Hungary | 0.08 (0.03, 0.12) | 0.05 (0.02, 0.09) |  | 5536 (2470, 8775) | 3998 (1663, 6883) | | 95.22 (42.31,150.62) | 52.27 (21.84, 89.54) | -1.79 (-2.21, -1.36) |
| Iceland | 0.01 (0, 0.03) | 0.02 (0.01, 0.04) |  | 8 (3, 18) | 21 (7, 40) |  | 6.48 (1.97,13.61) | 7.71 (2.53, 15.09) | 0.63 (-0.08, 1.33) |
| India | 0.09 (0.04, 0.13) | 0.15 (0.07, 0.22) |  | 582615 (254811, 932253) | 1756586 (809063, 2802405) | | 298.04 (127.85,482.19) | 348.44 (161.37, 552.76) | 0.45 (0.26, 0.64) |
| Indonesia | 0.01 (0, 0.02) | 0.04 (0.02, 0.06) |  | 5384 (1748, 10909) | 46994 (19320, 82804) | | 12.84 (4.1,25.89) | 54.11 (22.98, 93.85) | 4.77 (3.47, 6.08) |
| Iran (Islamic Republic of) | 0.08 (0.04, 0.12) | 0.09 (0.04, 0.14) |  | 7079 (3172, 11335) | 20344 (9507, 31223) | | 58.03 (26.05,92.61) | 57.64 (26.96, 88.74) | 0.29 (0.05, 0.53) |
| Iraq | 0.08 (0.04, 0.13) | 0.1 (0.05, 0.15) |  | 1458 (602, 2539) | 3426 (1580, 5511) | | 41.2 (17.59,71.45) | 34.6 (15.9, 54.86) | -1.1 (-1.44, -0.76) |
| Ireland | 0.03 (0.01, 0.05) | 0 (0, 0.01) |  | 708 (271, 1274) | 111 (31, 251) |  | 39.28 (15.03,71.07) | 3.18 (0.89, 7.24) | -8.26 (-9.17, -7.35) |
| Israel | 0.1 (0.05, 0.15) | 0.08 (0.03, 0.12) |  | 1347 (624, 2094) | 1649 (728, 2570) | | 61.71 (28.53,95.22) | 31.22 (13.8, 48.73) | -2.09 (-2.4, -1.78) |
| Italy | 0.1 (0.05, 0.15) | 0.08 (0.04, 0.13) |  | 29206 (13699, 44306) | 24580 (11420, 37870) | | 79.99 (37.58,121.98) | 36.02 (16.73, 55.31) | -3.15 (-3.65, -2.65) |
| Jamaica | 0.06 (0.03, 0.09) | 0.04 (0.02, 0.07) |  | 232 (103, 391) | 355 (134, 667) |  | 28.17 (12.54,47.55) | 25.21 (9.61, 46.97) | -1.27 (-2.38, -0.13) |
| Japan | 0.03 (0.01, 0.05) | 0.04 (0.02, 0.07) |  | 8919 (3672, 15385) | 23585 (9655, 40192) | | 13.69 (5.67,23.76) | 13.09 (5.41, 22.16) | -0.49 (-1.07, 0.09) |
| Jordan | 0.09 (0.04, 0.13) | 0.07 (0.03, 0.11) |  | 430 (186, 707) | 1038 (475, 1722) | | 74.14 (32.01,120.98) | 34.28 (15.48, 57.23) | -3.03 (-3.28, -2.78) |
| Kazakhstan | 0.08 (0.04, 0.13) | 0.06 (0.03, 0.1) |  | 6856 (3062, 10917) | 7415 (3200, 12735) | | 148.92 (65.85,235.77) | 117.94 (50.72, 201.36) | -1.24 (-1.87, -0.61) |
| Kenya | 0.01 (0, 0.01) | 0.03 (0.01, 0.06) |  | 252 (76, 576) | 3245 (1208, 5889) | | 6.92 (2.08,15.76) | 35.82 (13.34, 64.53) | 6.51 (4.55, 8.5) |
| Kiribati | 0.02 (0.01, 0.03) | 0.05 (0.02, 0.08) |  | 8 (3, 16) | 27 (12, 49) |  | 51.67 (17.92,99.3) | 100.1 (42.52, 173) | 2.17 (0.91, 3.45) |
| Kuwait | 0.07 (0.03, 0.11) | 0.08 (0.04, 0.13) |  | 81 (37, 128) | 311 (137, 521) |  | 28.39 (13.23,44.99) | 25.96 (11.45, 43.55) | -0.24 (-0.63, 0.16) |
| Kyrgyzstan | 0.12 (0.06, 0.18) | 0.1 (0.05, 0.16) |  | 3890 (1793, 5945) | 2152 (1003, 3400) | | 336.89 (154.63,513.86) | 121.6 (56.43, 191) | -4.31 (-4.93, -3.69) |
| Lao People's Democratic Republic | 0.02 (0.01, 0.04) | 0.05 (0.02, 0.08) |  | 454 (149, 923) | 1483 (588, 2671) | | 49.59 (16.37,100.34) | 80.82 (32.66, 142.76) | 1.69 (0.83, 2.56) |
| Latvia | 0.03 (0.01, 0.05) | 0.02 (0.01, 0.05) |  | 254 (92, 460) | 119 (42, 248) |  | 19.82 (7.14,36.09) | 8.36 (2.97, 17.29) | -3.15 (-3.95, -2.35) |
| Lebanon | 0.08 (0.04, 0.13) | 0.06 (0.02, 0.1) |  | 554 (231, 920) | 897 (338, 1629) |  | 54.86 (23.59,91.03) | 38.32 (14.5, 69.11) | -0.53 (-0.89, -0.17) |
| Lesotho | 0.02 (0.01, 0.04) | 0.05 (0.02, 0.09) |  | 220 (74, 459) | 589 (237, 1092) |  | 57.87 (19.3,118.64) | 124.39 (50.68, 226.35) | 3.07 (2.08, 4.07) |
| Liberia | 0.01 (0, 0.02) | 0.03 (0.01, 0.06) |  | 24 (7, 52) | 189 (69, 355) |  | 4.24 (1.27,9.09) | 19.74 (7.3, 35.84) | 5.68 (3.54, 7.88) |
| Libya | 0.06 (0.03, 0.09) | 0.07 (0.03, 0.11) |  | 342 (139, 580) | 1182 (502, 2025) | | 37.32 (14.92,63.55) | 47.53 (20.26, 81.87) | 0.5 (-0.01, 1.02) |
| Lithuania | 0.04 (0.01, 0.06) | 0.02 (0.01, 0.03) |  | 733 (280, 1283) | 180 (62, 358) |  | 42.79 (16.33,74.8) | 8.55 (2.95, 16.99) | -5.42 (-6.22, -4.62) |
| Luxembourg | 0.04 (0.01, 0.07) | 0.04 (0.02, 0.07) |  | 79 (30, 141) | 95 (39, 166) |  | 37.16 (14.07,66.56) | 21.06 (8.58, 36.36) | -2.2 (-2.74, -1.65) |
| Madagascar | 0 (0, 0.01) | 0.02 (0.01, 0.04) |  | 103 (23, 260) | 1385 (487, 2705) | | 4.22 (0.96,10.51) | 30.41 (10.99, 58.17) | 7.85 (5.35, 10.4) |
| Malawi | 0.01 (0, 0.02) | 0.03 (0.01, 0.05) |  | 182 (60, 391) | 923 (364, 1676) |  | 11.29 (3.75,23.68) | 32.24 (12.66, 58.21) | 4.81 (3.26, 6.39) |
| Malaysia | 0.02 (0.01, 0.03) | 0.04 (0.02, 0.06) |  | 920 (310, 1767) | 4256 (1823, 7551) | | 23.43 (7.83,44.84) | 35.03 (15.33, 61.27) | -0.29 (-1.48, 0.91) |
| Maldives | 0.02 (0, 0.03) | 0.06 (0.03, 0.1) |  | 14 (4, 32) | 77 (34, 126) |  | 32.68 (9.07,71.75) | 55.57 (25.15, 90.87) | 0.9 (0.16, 1.65) |
| Mali | 0.02 (0.01, 0.04) | 0.05 (0.02, 0.08) |  | 568 (196, 1129) | 2563 (1007, 4627) | | 28.06 (9.92,54.27) | 58.5 (23.68, 105.9) | 2.61 (1.19, 4.04) |
| Malta | 0.11 (0.05, 0.17) | 0.08 (0.04, 0.13) |  | 176 (81, 266) | 172 (74, 281) |  | 99.46 (45.81,149.44) | 39.82 (17.13, 65.51) | -4.05 (-4.61, -3.5) |
| Marshall Islands | 0 (0, 0) | 0 (0, 0) |  | 0 (0, 1) | 0 (0, 0) |  | 1.15 (0,10.3) | 0.12 (0, 2.08) | -6.54 (-10.17, -2.77) |
| Mauritania | 0.02 (0.01, 0.03) | 0.03 (0.01, 0.06) |  | 71 (25, 136) | 176 (67, 330) |  | 15.93 (5.63,30.46) | 18.27 (7.19, 34) | 0.44 (-0.92, 1.82) |
| Mauritius | 0 (0, 0.01) | 0 (0, 0) |  | 8 (0, 23) | 1 (0, 5) |  | 2.68 (0,7.96) | 0.08 (0, 0.63) | -11.89 (-15.28, -8.35) |
| Mexico | 0.12 (0.06, 0.18) | 0.06 (0.03, 0.09) |  | 19068 (9380, 28073) | 19062 (8436, 32048) | | 103.45 (51.03,152.27) | 37.61 (16.6, 63.1) | -3.97 (-4.23, -3.71) |
| Micronesia (Federated States of) | 0 (0, 0.01) | 0 (0, 0) |  | 1 (0, 6) | 0 (0, 2) |  | 4.51 (0,28.17) | 0.45 (0, 5.49) | -7 (-10.63, -3.23) |
| Monaco | 0.07 (0.02, 0.14) | 0.06 (0.02, 0.1) |  | 13 (3, 25) | 12 (5, 22) |  | 43.54 (11.13,81.38) | 25.84 (10.06, 46.5) | -0.96 (-1.63, -0.29) |
| Mongolia | 0.07 (0.03, 0.12) | 0.05 (0.02, 0.09) |  | 341 (141, 575) | 293 (123, 505) |  | 78.98 (33.74,134.39) | 31.61 (13.45, 53.75) | -3.95 (-4.67, -3.22) |
| Montenegro | 0.07 (0.03, 0.11) | 0.06 (0.03, 0.1) |  | 52 (23, 83) | 72 (31, 121) |  | 21.54 (9.61,34.82) | 17.56 (7.54, 29.36) | -0.79 (-1.2, -0.38) |
| Morocco | 0.04 (0.02, 0.07) | 0.08 (0.04, 0.12) |  | 1574 (609, 2941) | 8176 (3512, 13728) | | 25.51 (9.94,47.28) | 58.33 (25.35, 97.25) | 2.93 (2.48, 3.38) |
| Mozambique | 0.01 (0, 0.01) | 0.02 (0.01, 0.04) |  | 151 (41, 341) | 1253 (468, 2366) | | 5.92 (1.63,13.17) | 28.34 (10.77, 53.2) | 6.93 (5.13, 8.77) |
| Myanmar | 0.03 (0.01, 0.05) | 0.05 (0.02, 0.08) |  | 8912 (3469, 16894) | 23293 (9588, 39167) | | 90.73 (35.47,172.75) | 132.75 (54.73, 224.79) | 1.01 (0.43, 1.59) |
| Namibia | 0.05 (0.02, 0.08) | 0.05 (0.02, 0.09) |  | 318 (119, 592) | 486 (199, 848) |  | 103.06 (37.91,187.03) | 90.58 (37.36, 158.89) | 0.12 (-0.73, 0.97) |
| Nauru | 0 (0, 0) | 0 (0, 0) |  | 0 (0, 0) | 0 (0, 0) |  | 0.7 (0,0) | 0.46 (0, 0) | -3.58 (-6.32, -0.76) |
| Nepal | 0.12 (0.06, 0.18) | 0.17 (0.08, 0.25) |  | 20835 (8928, 34785) | 57015 (26327, 90440) | | 497.43 (212.61,819.68) | 596.17 (273.26, 937.46) | 0.77 (0.54, 1.01) |
| Netherlands | 0.03 (0.01, 0.05) | 0.03 (0.01, 0.05) |  | 2734 (1054, 4923) | 3213 (1239, 5780) | | 33.86 (13.08,60.81) | 20.19 (7.78, 36.29) | -2.76 (-3.53, -1.99) |
| New Zealand | 0 (0, 0.01) | 0.01 (0, 0.02) |  | 83 (16, 196) | 224 (69, 468) |  | 4.88 (0.94,11.6) | 5.97 (1.84, 12.48) | -0.24 (-1.6, 1.14) |
| Nicaragua | 0.02 (0.01, 0.04) | 0.02 (0.01, 0.04) |  | 68 (25, 130) | 276 (92, 532) |  | 11.66 (4.26,22.4) | 17.11 (5.76, 32.85) | 0.71 (-0.05, 1.47) |
| Niger | 0.05 (0.02, 0.08) | 0.06 (0.03, 0.1) |  | 752 (283, 1574) | 2012 (801, 4093) | | 57.53 (21.9,115.68) | 58.74 (23.81, 114.87) | 0.74 (-0.46, 1.97) |
| Nigeria | 0.06 (0.03, 0.09) | 0.08 (0.03, 0.12) |  | 9634 (3814, 16774) | 19345 (7853, 34454) | | 49.2 (19.6,85.39) | 54.16 (22.21, 95.89) | 0.72 (-0.01, 1.46) |
| Niue | 0.02 (0, 0.03) | 0.02 (0, 0.04) |  | 0 (0, 1) | 0 (0, 0) |  | 33.44 (8.46,74.86) | 21.4 (5.31, 47.57) | -3.33 (-4.04, -2.62) |
| North Macedonia | 0.08 (0.04, 0.13) | 0.03 (0.01, 0.05) |  | 595 (269, 953) | 221 (82, 399) |  | 72.13 (32.59,115.75) | 15.81 (5.85, 28.31) | -4.55 (-5.62, -3.47) |
| Northern Mariana Islands | 0.03 (0.01, 0.07) | 0.07 (0.03, 0.13) |  | 6 (2, 12) | 17 (7, 31) |  | 56.85 (16.83,115.3) | 77.36 (32.9, 133.11) | 0.38 (-0.45, 1.22) |
| Norway | 0.03 (0.01, 0.05) | 0.02 (0.01, 0.03) |  | 458 (187, 804) | 467 (168, 872) |  | 15.13 (6.18,26.54) | 10.22 (3.67, 19.07) | -1.81 (-2.84, -0.78) |
| Oman | 0.07 (0.03, 0.12) | 0.06 (0.03, 0.1) |  | 205 (78, 381) | 285 (123, 463) |  | 70.8 (27.96,131.81) | 46.73 (19.64, 75.89) | -0.67 (-1.07, -0.25) |
| Pakistan | 0.1 (0.05, 0.15) | 0.14 (0.07, 0.2) |  | 79636 (34959, 130854) | 157225 (74927, 249168) | | 268.98 (117.82,444.62) | 322.71 (152.69, 505.11) | 0.58 (0.35, 0.82) |
| Palau | 0 (0, 0.01) | 0 (0, 0.01) |  | 0 (0, 1) | 0 (0, 1) |  | 3.56 (0,24.1) | 1.29 (0, 10.97) | -3.12 (-6.22, 0.08) |
| Palestine | 0.09 (0.05, 0.15) | 0.08 (0.04, 0.12) |  | 328 (143, 587) | 493 (227, 805) |  | 90.05 (40.08,161.01) | 52.66 (24.86, 84.91) | -1.88 (-2.07, -1.68) |
| Panama | 0.03 (0.01, 0.06) | 0.02 (0.01, 0.05) |  | 107 (43, 197) | 155 (50, 318) |  | 15.01 (5.98,27.69) | 7.91 (2.57, 16.14) | -4.69 (-5.95, -3.42) |
| Papua New Guinea | 0 (0, 0) | 0 (0, 0) |  | 21 (4, 62) | 25 (4, 72) |  | 2.5 (0.44,7.28) | 1.16 (0.17, 3.35) | -4.02 (-6.98, -0.97) |
| Paraguay | 0.03 (0.01, 0.05) | 0.05 (0.02, 0.08) |  | 113 (43, 214) | 665 (254, 1211) |  | 11.64 (4.5,22.15) | 26.87 (10.16, 48.55) | 3.64 (2.5, 4.8) |
| Peru | 0.01 (0, 0.01) | 0.02 (0.01, 0.04) |  | 133 (39, 295) | 699 (267, 1350) |  | 2.38 (0.7,5.22) | 4.58 (1.74, 8.86) | 2.46 (1.43, 3.5) |
| Philippines | 0 (0, 0.01) | 0 (0, 0.01) |  | 877 (224, 1942) | 1862 (509, 4167) | | 6.96 (1.79,15.27) | 5.72 (1.57, 12.75) | 0.17 (-1.52, 1.88) |
| Poland | 0.05 (0.02, 0.09) | 0.05 (0.02, 0.08) |  | 10279 (4319, 16993) | 7341 (3048, 12374) | | 58.93 (24.73,97.27) | 25.3 (10.51, 42.76) | -3.12 (-3.48, -2.75) |
| Portugal | 0.03 (0.01, 0.05) | 0.03 (0.01, 0.06) |  | 1494 (615, 2601) | 2154 (831, 3841) | | 26.73 (11.05,46.54) | 19.87 (7.73, 35.63) | -0.14 (-1.2, 0.94) |
| Puerto Rico | 0.01 (0, 0.03) | 0 (0, 0.01) |  | 126 (40, 257) | 47 (7, 117) |  | 7.68 (2.44,15.6) | 1.45 (0.23, 3.64) | -7.83 (-9.57, -6.06) |
| Qatar | 0.07 (0.03, 0.11) | 0.06 (0.03, 0.1) |  | 33 (14, 60) | 170 (81, 293) |  | 67.25 (28.77,122.61) | 45.95 (22.87, 77.92) | -1.36 (-1.84, -0.88) |
| Republic of Korea | 0.05 (0.02, 0.08) | 0.07 (0.04, 0.11) |  | 3903 (1698, 6587) | 13070 (6148, 20559) | | 44.89 (19.11,76.09) | 39.67 (18.75, 62.9) | -1.71 (-2.77, -0.64) |
| Republic of Moldova | 0.09 (0.04, 0.14) | 0.03 (0.01, 0.06) |  | 2473 (1141, 3883) | 464 (184, 856) |  | 138.76 (63.54,216.92) | 20 (7.94, 36.87) | -6.89 (-7.55, -6.24) |
| Romania | 0.08 (0.04, 0.13) | 0.02 (0.01, 0.03) |  | 15594 (6796, 24632) | 1759 (588, 3409) | | 130.99 (57.19,207.54) | 11.32 (3.78, 21.92) | -7.78 (-8.42, -7.14) |
| Russian Federation | 0.07 (0.03, 0.11) | 0.03 (0.01, 0.05) |  | 50061 (21337, 80544) | 16519 (6390, 29339) | | 85.19 (36.33,136.81) | 19.29 (7.51, 34.23) | -5.52 (-6.13, -4.9) |
| Rwanda | 0.03 (0.01, 0.05) | 0.06 (0.03, 0.1) |  | 745 (267, 1475) | 1745 (731, 3037) | | 61.2 (22.28,120.47) | 79.28 (32.99, 134.57) | 0.75 (-1.01, 2.54) |
| Saint Kitts and Nevis | 0.02 (0, 0.06) | 0 (0, 0) |  | 1 (0, 4) | 0 (0, 0) |  | 9.08 (0.44,27.03) | 0.01 (0, 0) | -25.45 (-28.88, -21.85) |
| Saint Lucia | 0.02 (0, 0.06) | 0 (0, 0.01) |  | 5 (0, 18) | 1 (0, 8) |  | 13.14 (0,51.99) | 0.67 (0, 8.28) | -10.82 (-12.45, -9.17) |
| Saint Vincent and the Grenadines | 0.01 (0, 0.05) | 0 (0, 0.02) |  | 1 (0, 5) | 0 (0, 3) |  | 4.34 (0,16.05) | 0.44 (0, 5.27) | -9.64 (-11.33, -7.91) |
| Samoa | 0.02 (0, 0.07) | 0.02 (0, 0.04) |  | 19 (0, 72) | 16 (4, 34) |  | 47.25 (0,185.67) | 24.25 (6.46, 51.2) | -3.76 (-4.37, -3.14) |
| San Marino | 0.08 (0.03, 0.12) | 0.06 (0.03, 0.1) |  | 6 (2, 9) | 7 (3, 13) |  | 38.55 (15.02,63.63) | 23.23 (9.36, 42.06) | -1.69 (-2.38, -1) |
| Sao Tome and Principe | 0.01 (0, 0.04) | 0.04 (0.01, 0.07) |  | 7 (1, 20) | 34 (12, 65) |  | 23.69 (4.08,71.83) | 75.06 (27.32, 142.53) | 4.61 (3.23, 6.01) |
| Saudi Arabia | 0.09 (0.04, 0.13) | 0.09 (0.04, 0.14) |  | 1933 (797, 3708) | 4867 (2177, 7936) | | 66.99 (27.63,125.94) | 51.73 (22.8, 81.75) | -0.92 (-1.17, -0.67) |
| Senegal | 0.02 (0.01, 0.04) | 0.04 (0.02, 0.07) |  | 354 (115, 752) | 1240 (535, 2233) | | 23.45 (7.82,48.85) | 37.11 (16.03, 65.83) | 2 (0.95, 3.07) |
| Serbia | 0.08 (0.04, 0.13) | 0.02 (0.01, 0.04) |  | 3571 (1620, 5776) | 1212 (453, 2184) | | 70.7 (31.76,114.29) | 16.83 (6.25, 30.13) | -4.57 (-5.4, -3.74) |
| Seychelles | 0 (0, 0.02) | 0.01 (0, 0.06) |  | 0 (0, 6) | 5 (0, 25) |  | 1.87 (0,25.84) | 10.6 (0, 54.49) | 5.18 (2.65, 7.78) |
| Sierra Leone | 0.02 (0.01, 0.04) | 0.04 (0.02, 0.07) |  | 210 (74, 420) | 620 (236, 1175) |  | 22.19 (7.88,43.35) | 36.75 (13.9, 67.91) | 2.46 (1.19, 3.74) |
| Singapore | 0.02 (0, 0.07) | 0.04 (0.01, 0.07) |  | 309 (10, 900) | 478 (137, 948) |  | 37.83 (1.21,109.65) | 14.17 (4.09, 28.41) | -4.16 (-5.95, -2.34) |
| Slovakia | 0.07 (0.03, 0.11) | 0.05 (0.02, 0.09) |  | 987 (441, 1632) | 909 (395, 1575) |  | 40.05 (18.08,66.42) | 24.09 (10.55, 41.84) | -0.95 (-1.54, -0.35) |
| Slovenia | 0.08 (0.04, 0.13) | 0.07 (0.04, 0.12) |  | 799 (352, 1351) | 576 (258, 964) |  | 88.33 (38.91,149.88) | 30.65 (13.74, 51.32) | -4.88 (-5.38, -4.38) |
| Solomon Islands | 0 (0, 0) | 0 (0, 0) |  | 2 (0, 6) | 1 (0, 8) |  | 2.07 (0,8.21) | 0.99 (0, 5.17) | -2.74 (-5.66, 0.27) |
| Somalia | 0.01 (0, 0.01) | 0.03 (0.01, 0.05) |  | 121 (34, 287) | 1228 (408, 2658) | | 11.55 (3.47,27.08) | 48.38 (16.18, 104.22) | 4.51 (3.28, 5.75) |
| South Africa | 0.02 (0.01, 0.03) | 0.04 (0.02, 0.06) |  | 2163 (809, 3917) | 7174 (3138, 12052) | | 25.46 (9.44,46.4) | 41.65 (18.12, 70.21) | 1.61 (0.75, 2.48) |
| South Sudan | 0.06 (0.03, 0.1) | 0.05 (0.02, 0.08) |  | 958 (371, 1667) | 765 (290, 1460) |  | 80.37 (32.26,139.27) | 43.86 (17.16, 81.09) | -1.83 (-2.59, -1.06) |
| Spain | 0.04 (0.02, 0.07) | 0.07 (0.03, 0.11) |  | 11155 (4761, 18715) | 21874 (9839, 34511) | | 49.56 (21.27,83.16) | 48.55 (21.8, 76.94) | -0.36 (-0.84, 0.11) |
| Sri Lanka | 0.01 (0, 0.01) | 0.03 (0.01, 0.06) |  | 280 (75, 622) | 3225 (1156, 5919) | | 5.5 (1.5,12.28) | 30.66 (11.34, 56.04) | 5.42 (4.5, 6.36) |
| Sudan | 0.04 (0.01, 0.06) | 0.06 (0.03, 0.1) |  | 2032 (620, 4007) | 5332 (1942, 9854) | | 43.62 (13.13,86.41) | 55.8 (20.43, 101.62) | 1.05 (0.49, 1.61) |
| Suriname | 0 (0, 0) | 0 (0, 0.01) |  | 1 (0, 2) | 3 (0, 8) |  | 0.74 (0.13,1.85) | 1.13 (0.16, 3.26) | 0.14 (-1.8, 2.12) |
| Sweden | 0.02 (0.01, 0.04) | 0.02 (0.01, 0.03) |  | 611 (229, 1118) | 679 (237, 1265) |  | 9.07 (3.4,16.53) | 6.44 (2.26, 11.99) | -1.75 (-2.65, -0.85) |
| Switzerland | 0.07 (0.03, 0.11) | 0.06 (0.03, 0.09) |  | 2244 (1030, 3545) | 1899 (838, 3026) | | 52.04 (23.92,82.34) | 23.19 (10.24, 36.99) | -2.79 (-3.12, -2.45) |
| Syrian Arab Republic | 0.09 (0.04, 0.13) | 0.08 (0.04, 0.12) |  | 1626 (724, 2726) | 2958 (1297, 5189) | | 60.11 (26.98,101.43) | 49.88 (21.93, 86.03) | -0.8 (-0.98, -0.62) |
| Taiwan (Province of China) | 0.04 (0.02, 0.07) | 0.05 (0.02, 0.09) |  | 2118 (877, 3568) | 4856 (2102, 8626) | | 31.72 (13.01,53.24) | 27 (11.67, 47.97) | -0.51 (-1.38, 0.36) |
| Tajikistan | 0.13 (0.06, 0.19) | 0.1 (0.04, 0.14) |  | 1737 (807, 2700) | 1758 (807, 2844) | | 142.74 (66.93,222.03) | 82.12 (37.67, 134.18) | -1.67 (-2.18, -1.15) |
| Thailand | 0.01 (0, 0.02) | 0.05 (0.02, 0.08) |  | 3642 (1194, 7292) | 17711 (7360, 30406) | | 23.87 (8.17,47.7) | 40.5 (16.69, 69.1) | 1.54 (0.79, 2.28) |
| Timor-Leste | 0.01 (0, 0.02) | 0.02 (0.01, 0.04) |  | 18 (5, 43) | 122 (35, 247) |  | 15.38 (4.06,33.54) | 33.12 (9.62, 67.48) | 2.05 (0.42, 3.7) |
| Togo | 0.04 (0.01, 0.06) | 0.07 (0.03, 0.11) |  | 224 (86, 412) | 1108 (430, 1936) | | 42.18 (16.39,76.95) | 77.86 (32.11, 132.87) | 2.58 (1.41, 3.76) |
| Tokelau | 0.02 (0, 0.04) | 0.01 (0, 0.03) |  | 0 (0, 0) | 0 (0, 0) |  | 31.27 (7.59,72.21) | 15.72 (3.86, 34.37) | -3.86 (-4.56, -3.15) |
| Tonga | 0.02 (0, 0.04) | 0.02 (0, 0.04) |  | 7 (1, 16) | 8 (2, 17) |  | 29.28 (6.28,68.25) | 22.69 (4.68, 50.29) | -2.47 (-3.26, -1.67) |
| Trinidad and Tobago | 0.01 (0, 0.03) | 0 (0, 0.02) |  | 21 (3, 56) | 14 (0, 50) |  | 5.52 (0.71,15.05) | 1.68 (0.04, 5.81) | -5.52 (-6.69, -4.33) |
| Tunisia | 0.08 (0.04, 0.12) | 0.07 (0.03, 0.11) |  | 1102 (474, 1841) | 2427 (1021, 4113) | | 47.89 (20.37,79.6) | 42.67 (18.1, 73.07) | -0.83 (-1.12, -0.54) |
| Turkey | 0.09 (0.04, 0.13) | 0.07 (0.03, 0.11) |  | 21393 (9762, 34905) | 32820 (14750, 55142) | | 133.56 (61.76,215.58) | 84.01 (37.71, 142.16) | -0.99 (-1.45, -0.52) |
| Turkmenistan | 0.09 (0.04, 0.14) | 0.06 (0.03, 0.1) |  | 829 (372, 1295) | 431 (187, 742) |  | 108.08 (48.47,166.47) | 25.25 (11.11, 42.94) | -5.92 (-6.54, -5.29) |
| Tuvalu | 0 (0, 0.01) | 0 (0, 0.01) |  | 0 (0, 1) | 0 (0, 1) |  | 6.26 (0,24.98) | 6.31 (0, 20.49) | -0.64 (-2.36, 1.1) |
| Uganda | 0.03 (0.01, 0.05) | 0.05 (0.02, 0.08) |  | 1362 (480, 2762) | 3124 (1162, 5602) | | 47.64 (17.16,96.05) | 57.78 (21.85, 102.46) | 0.54 (-1.4, 2.52) |
| Ukraine | 0.08 (0.04, 0.13) | 0.04 (0.02, 0.07) |  | 37488 (16077, 59952) | 7374 (2742, 13322) | | 151.01 (64.71,240.95) | 26.14 (9.75, 46.93) | -7.48 (-8.03, -6.91) |
| United Arab Emirates | 0.1 (0.05, 0.16) | 0.1 (0.05, 0.15) |  | 587 (206, 1026) | 5201 (1902, 9348) | | 128.2 (53.93,219.74) | 97.36 (42.77, 164.94) | -0.84 (-1.33, -0.34) |
| United Kingdom | 0.02 (0.01, 0.04) | 0.01 (0, 0.02) |  | 8148 (2897, 15185) | 4397 (1330, 9030) | | 21.66 (7.7,40.41) | 7.36 (2.22, 15.1) | -2.55 (-3.38, -1.71) |
| United Republic of Tanzania | 0.01 (0, 0.01) | 0.03 (0.01, 0.05) |  | 330 (109, 683) | 2284 (923, 4091) | | 6.81 (2.26,13.82) | 21.66 (8.77, 38.23) | 4.78 (3.07, 6.53) |
| United States of America | 0.06 (0.03, 0.1) | 0.04 (0.02, 0.07) |  | 90330 (41646, 141192) | 102245 (44217, 169975) | | 66.35 (30.54,103.73) | 39.79 (17.22, 66.13) | -1.9 (-2.32, -1.48) |
| United States Virgin Islands | 0.01 (0, 0.03) | 0 (0, 0) |  | 1 (0, 3) | 0 (0, 1) |  | 3.92 (1.16,8.57) | 0.44 (0, 1.41) | -9.14 (-11.24, -6.99) |
| Uruguay | 0.01 (0, 0.03) | 0.02 (0, 0.03) |  | 197 (60, 434) | 369 (102, 779) |  | 11.68 (3.59,25.69) | 16.29 (4.52, 34.23) | 0.62 (-0.04, 1.28) |
| Uzbekistan | 0.11 (0.05, 0.17) | 0.08 (0.04, 0.13) |  | 4906 (2177, 7951) | 3354 (1540, 5412) | | 107.62 (47.03,174.87) | 42.76 (19.65, 67.59) | -4.39 (-5.06, -3.72) |
| Vanuatu | 0 (0, 0.01) | 0 (0, 0.01) |  | 4 (0, 12) | 9 (1, 26) |  | 11.13 (1.36,32.03) | 10.84 (1.6, 29.88) | -0.77 (-2.35, 0.84) |
| Venezuela (Bolivarian Republic of) | 0.03 (0.01, 0.05) | 0.02 (0.01, 0.04) |  | 534 (204, 972) | 1662 (614, 3218) | | 12.63 (4.87,22.84) | 12.94 (4.82, 25.01) | -1.67 (-2.52, -0.8) |
| Viet Nam | 0.02 (0.01, 0.03) | 0.03 (0.01, 0.06) |  | 4503 (1137, 9276) | 15858 (4101, 29817) | | 29.91 (7.48,61.36) | 47.36 (12.51, 89.22) | 2.21 (1.46, 2.96) |
| Yemen | 0.04 (0.02, 0.07) | 0.07 (0.03, 0.1) |  | 1081 (355, 2219) | 4205 (1737, 7456) | | 51.14 (16.77,103.06) | 67.58 (27.83, 117.83) | 1.28 (0.87, 1.7) |
| Zambia | 0.03 (0.01, 0.06) | 0.05 (0.02, 0.08) |  | 526 (198, 964) | 1436 (587, 2466) | | 39.18 (14.81,72.77) | 51.09 (20.47, 86.97) | 1.54 (0.52, 2.57) |
| Zimbabwe | 0.02 (0.01, 0.03) | 0.04 (0.02, 0.06) |  | 279 (98, 531) | 916 (363, 1651) |  | 16.74 (5.91,31.72) | 35.36 (14.09, 63.4) | 3.72 (2.47, 4.98) |

(B) Women

| Country | Percentage (%) | |  | Death number | |  | Age-standardized DALY rate | | |
| --- | --- | --- | --- | --- | --- | --- | --- | --- | --- |
|  | 1990  (uncertainty interval) | 2019  (uncertainty interval) |  | 1990  (uncertainty interval) | 2019  (uncertainty interval) |  | 1990  (uncertainty interval)  (per 100,000 person-years) | 2019  (uncertainty interval)  (per 100,000 person-years) | Annual percentage change, 1990-2019 (confidence interval) (%) |
| Afghanistan | 0.1 (0.05, 0.15) | 0.08 (0.04, 0.12) |  | 3613 (1079, 7074) | 5717 (2022, 10173) | | 108.66 (33.04,209.41) | 89.63 (31.58, 158.21) | -0.57 (-0.91, -0.22) |
| Albania | 0.08 (0.04, 0.13) | 0.03 (0.01, 0.05) |  | 402 (160, 655) | 114 (41, 222) |  | 39.6 (15.66,64.73) | 4.89 (1.77, 9.5) | -6.43 (-6.98, -5.88) |
| Algeria | 0.06 (0.03, 0.09) | 0.07 (0.03, 0.11) |  | 1775 (673, 3211) | 4260 (1901, 7173) | | 35.24 (13.19,63.25) | 30.28 (13.51, 50.6) | -0.67 (-0.96, -0.38) |
| American Samoa | 0.01 (0, 0.03) | 0.02 (0, 0.03) |  | 2 (0, 4) | 3 (1, 5) |  | 15.22 (4.03,32.75) | 10.74 (2.58, 23.28) | -2.88 (-3.55, -2.21) |
| Andorra | 0.04 (0.02, 0.08) | 0.05 (0.02, 0.08) |  | 4 (1, 7) | 12 (5, 22) |  | 14.84 (5.01,28.88) | 16.03 (6.35, 28.27) | 0.34 (0.05, 0.63) |
| Angola | 0.06 (0.03, 0.09) | 0.04 (0.01, 0.06) |  | 1220 (421, 2444) | 1545 (552, 2836) | | 71.17 (25.53,140.26) | 30.14 (11.17, 55.56) | -2.55 (-3.3, -1.79) |
| Antigua and Barbuda | 0.02 (0, 0.04) | 0 (0, 0) |  | 0 (0, 1) | 0 (0, 0) |  | 1.42 (0.26,3.31) | 0.01 (0, 0.14) | -16.96 (-19.56, -14.26) |
| Argentina | 0.02 (0.01, 0.03) | 0.02 (0.01, 0.04) |  | 1132 (404, 2201) | 3289 (1264, 6039) | | 6.44 (2.31,12.51) | 10.37 (3.97, 19.02) | 1.67 (1.32, 2.03) |
| Armenia | 0.07 (0.03, 0.11) | 0.09 (0.04, 0.14) |  | 535 (215, 926) | 886 (369, 1475) |  | 38.08 (15.39,65.68) | 36.54 (15.3, 60.64) | 0.2 (-0.26, 0.66) |
| Australia | 0.01 (0, 0.01) | 0.01 (0, 0.01) |  | 296 (91, 613) | 714 (220, 1477) |  | 2.67 (0.82,5.53) | 3.02 (0.93, 6.2) | -0.15 (-0.94, 0.65) |
| Austria | 0.05 (0.02, 0.08) | 0.05 (0.02, 0.09) |  | 923 (415, 1549) | 1619 (683, 2702) | | 11.57 (5.2,19.48) | 15.45 (6.56, 25.73) | 0.86 (0.44, 1.28) |
| Azerbaijan | 0.06 (0.03, 0.09) | 0.06 (0.03, 0.09) |  | 888 (395, 1514) | 1084 (463, 2087) | | 30.42 (13.44,51.63) | 26.21 (11.18, 51.13) | -1.42 (-2.15, -0.68) |
| Bahamas | 0.02 (0.01, 0.05) | 0.02 (0, 0.05) |  | 3 (1, 6) | 6 (1, 14) |  | 3.38 (0.87,7.42) | 2.99 (0.68, 6.88) | -0.9 (-1.73, -0.06) |
| Bahrain | 0.08 (0.03, 0.14) | 0.09 (0.04, 0.14) |  | 57 (20, 102) | 138 (60, 250) |  | 91.11 (31.74,164.81) | 56.5 (25.53, 98.33) | -2.22 (-2.84, -1.6) |
| Bangladesh | 0.06 (0.03, 0.1) | 0.11 (0.05, 0.17) |  | 20608 (7931, 40605) | 62168 (26487, 135511) | | 106.03 (41.27,208.53) | 106.34 (45.55, 234.24) | -0.29 (-0.54, -0.04) |
| Barbados | 0.01 (0, 0.03) | 0 (0, 0) |  | 2 (0, 6) | 0 (0, 1) |  | 1.34 (0.16,3.38) | 0.03 (0, 0.28) | -14.34 (-16.23, -12.42) |
| Belarus | 0.04 (0.02, 0.07) | 0.02 (0.01, 0.04) |  | 1858 (584, 3429) | 416 (138, 1037) |  | 21.82 (6.87,40.27) | 3.85 (1.27, 9.79) | -7.13 (-7.61, -6.65) |
| Belgium | 0.02 (0.01, 0.04) | 0.03 (0.01, 0.05) |  | 861 (318, 1571) | 1685 (663, 2976) | | 8.99 (3.36,16.4) | 12.6 (5.02, 22.3) | 0.88 (0.14, 1.62) |
| Belize | 0.04 (0.01, 0.06) | 0.03 (0.01, 0.05) |  | 5 (2, 8) | 12 (4, 23) |  | 10.03 (3.98,17.67) | 8.67 (3.14, 16.8) | -0.96 (-1.74, -0.18) |
| Benin | 0.03 (0.01, 0.05) | 0.05 (0.02, 0.09) |  | 264 (100, 485) | 927 (396, 1671) |  | 26.08 (10.09,47.87) | 37.42 (16.12, 66.33) | 1.6 (0.46, 2.75) |
| Bermuda | 0.1 (0.05, 0.15) | 0.05 (0.02, 0.09) |  | 5 (2, 8) | 4 (2, 7) |  | 14.69 (6.6,22.88) | 5.1 (1.99, 9.18) | -4.35 (-4.85, -3.84) |
| Bhutan | 0.07 (0.03, 0.12) | 0.11 (0.05, 0.16) |  | 215 (85, 373) | 548 (247, 871) |  | 192.64 (76.66,335.77) | 222.29 (100.28, 352.85) | 0.43 (0.18, 0.68) |
| Bolivia (Plurinational State of) | 0.02 (0.01, 0.04) | 0.04 (0.02, 0.07) |  | 287 (106, 534) | 953 (365, 1764) |  | 18.43 (6.84,34) | 23.24 (9.01, 42.98) | 1.12 (-0.53, 2.79) |
| Bosnia and Herzegovina | 0.07 (0.03, 0.12) | 0.05 (0.02, 0.08) |  | 795 (353, 1268) | 493 (210, 847) |  | 38.58 (17.03,61.74) | 14.46 (6.19, 24.64) | -4.16 (-4.6, -3.73) |
| Botswana | 0.02 (0.01, 0.04) | 0.03 (0.01, 0.05) |  | 67 (22, 152) | 173 (56, 340) |  | 22.85 (7.61,51.73) | 24.77 (8.21, 48.2) | 1.05 (0.23, 1.87) |
| Brazil | 0.02 (0.01, 0.03) | 0.04 (0.02, 0.07) |  | 6277 (2259, 11589) | 27647 (11637, 47648) | | 14.15 (5.07,26.23) | 21.32 (8.97, 36.77) | 0.18 (-0.67, 1.05) |
| Brunei Darussalam | 0 (0, 0.01) | 0.01 (0, 0.02) |  | 1 (0, 5) | 5 (0, 17) |  | 3.88 (0.24,12.73) | 4.54 (0.2, 14.87) | -2.08 (-4.1, -0.01) |
| Bulgaria | 0.08 (0.04, 0.12) | 0.03 (0.01, 0.06) |  | 2094 (946, 3297) | 894 (360, 1601) |  | 32.75 (14.64,51.73) | 10.64 (4.38, 19.11) | -3.63 (-4.68, -2.57) |
| Burkina Faso | 0.02 (0.01, 0.04) | 0.04 (0.02, 0.06) |  | 314 (120, 600) | 1015 (433, 1797) | | 14.17 (5.23,27.08) | 21.05 (9.19, 37.61) | 1.46 (0.24, 2.7) |
| Burundi | 0.02 (0.01, 0.04) | 0.05 (0.02, 0.08) |  | 293 (88, 604) | 866 (319, 1617) |  | 23.26 (7.33,47.79) | 42.38 (15.87, 78.01) | 2.44 (1, 3.9) |
| Cabo Verde | 0.02 (0, 0.05) | 0.02 (0, 0.06) |  | 14 (0, 52) | 23 (1, 60) |  | 10.56 (0,39.25) | 9.4 (0.39, 25) | -0.4 (-1.87, 1.08) |
| Cambodia | 0.01 (0, 0.02) | 0.03 (0.01, 0.05) |  | 147 (43, 324) | 1099 (423, 1984) | | 6.36 (1.87,13.81) | 17.9 (6.95, 32.14) | 4.33 (3.22, 5.44) |
| Cameroon | 0.04 (0.02, 0.06) | 0.04 (0.02, 0.07) |  | 736 (294, 1346) | 1830 (725, 3358) | | 33.86 (13.66,60.89) | 31.33 (12.82, 56.67) | -0.01 (-1.41, 1.41) |
| Canada | 0.03 (0.01, 0.04) | 0.03 (0.01, 0.05) |  | 1734 (662, 3069) | 4015 (1523, 7258) | | 9.2 (3.51,16.3) | 10.13 (3.93, 18.29) | 0.14 (-0.55, 0.83) |
| Central African Republic | 0.08 (0.04, 0.13) | 0.08 (0.03, 0.12) |  | 592 (204, 1193) | 915 (319, 1981) |  | 106.29 (37.76,210.73) | 89.81 (31.58, 191.36) | -0.7 (-1.62, 0.22) |
| Chad | 0.04 (0.02, 0.07) | 0.04 (0.02, 0.07) |  | 478 (189, 895) | 865 (374, 1592) |  | 33.44 (13.31,62.2) | 35.06 (15.48, 63.14) | 0.9 (-0.12, 1.92) |
| Chile | 0.03 (0.01, 0.05) | 0.02 (0.01, 0.04) |  | 529 (197, 945) | 763 (249, 1505) |  | 10.33 (3.84,18.36) | 5.56 (1.82, 10.97) | -2.7 (-3.24, -2.15) |
| China | 0.07 (0.03, 0.12) | 0.06 (0.03, 0.1) |  | 908700 (388776, 1531654) | 541318 (241361, 936881) | | 241.64 (104.79,407.53) | 55.65 (24.86, 96.02) | -5.11 (-5.82, -4.39) |
| Colombia | 0.01 (0.01, 0.03) | 0.01 (0, 0.03) |  | 719 (251, 1407) | 1728 (537, 3511) | | 8.7 (3.06,16.9) | 5.75 (1.79, 11.71) | -3.22 (-4.15, -2.28) |
| Comoros | 0 (0, 0.02) | 0.01 (0, 0.04) |  | 1 (0, 12) | 14 (0, 58) |  | 0.98 (0,11.12) | 5.48 (0, 23.08) | 5.76 (3.08, 8.51) |
| Congo | 0.02 (0.01, 0.03) | 0.04 (0.02, 0.07) |  | 99 (27, 236) | 435 (142, 864) |  | 18.59 (5.14,46.24) | 37.35 (12.55, 74.26) | 3.5 (1.39, 5.65) |
| Cook Islands | 0.01 (0, 0.05) | 0.01 (0, 0.02) |  | 1 (0, 4) | 1 (0, 2) |  | 16.76 (0,63.21) | 7.29 (1.87, 15.87) | -4.33 (-4.91, -3.75) |
| Costa Rica | 0.02 (0.01, 0.04) | 0.02 (0.01, 0.04) |  | 67 (22, 138) | 169 (47, 360) |  | 7.96 (2.58,16.24) | 5.99 (1.65, 12.71) | -3.24 (-4.51, -1.94) |
| Croatia | 0.06 (0.03, 0.1) | 0.06 (0.03, 0.09) |  | 584 (271, 956) | 865 (369, 1463) |  | 15.35 (7.16,24.88) | 15.3 (6.58, 25.62) | 0.22 (0, 0.45) |
| Cuba | 0.02 (0.01, 0.04) | 0.02 (0.01, 0.04) |  | 329 (116, 628) | 859 (265, 1726) |  | 6.34 (2.24,12.13) | 8.47 (2.62, 16.94) | 0.42 (-0.52, 1.37) |
| Cyprus | 0.09 (0.04, 0.14) | 0.07 (0.03, 0.11) |  | 187 (76, 311) | 239 (104, 382) |  | 53.63 (21.42,90.41) | 24.64 (10.51, 39.75) | -2.94 (-3.42, -2.45) |
| Czechia | 0.05 (0.02, 0.09) | 0.05 (0.02, 0.08) |  | 1088 (500, 1834) | 1802 (742, 3052) | | 13.12 (5.88,21.97) | 14.58 (6.05, 24.99) | 1.05 (0.59, 1.51) |
| Côte d'Ivoire | 0.01 (0, 0.02) | 0.03 (0.01, 0.05) |  | 168 (61, 346) | 921 (381, 1705) |  | 9.78 (3.5,20.09) | 19.52 (8.04, 35.02) | 2.8 (0.77, 4.88) |
| Democratic People's Republic of Korea | 0.06 (0.03, 0.1) | 0.1 (0.05, 0.15) |  | 14254 (6133, 25875) | 31315 (14143, 51240) | | 168.25 (71.79,307.05) | 161.5 (73.41, 263.29) | -0.49 (-0.83, -0.15) |
| Democratic Republic of the Congo | 0.04 (0.02, 0.07) | 0.06 (0.03, 0.1) |  | 3307 (1070, 7218) | 10668 (3773, 24711) | | 45.82 (14.8,100.12) | 62.02 (22.3, 146.34) | 1.66 (0.25, 3.09) |
| Denmark | 0.02 (0.01, 0.04) | 0.03 (0.01, 0.05) |  | 730 (267, 1337) | 1281 (439, 2384) | | 16.39 (5.92,30.16) | 19.53 (6.62, 36.17) | -0.75 (-1.55, 0.06) |
| Djibouti | 0.01 (0, 0.03) | 0.03 (0.01, 0.06) |  | 6 (1, 14) | 42 (9, 93) |  | 9.82 (2.13,23.14) | 17.78 (3.66, 38.59) | 2.1 (1.31, 2.9) |
| Dominica | 0.02 (0, 0.05) | 0 (0, 0.01) |  | 1 (0, 4) | 0 (0, 1) |  | 3.22 (0.07,9.57) | 0.13 (0, 1.19) | -11.14 (-13.16, -9.07) |
| Dominican Republic | 0.03 (0.01, 0.05) | 0.02 (0.01, 0.04) |  | 128 (50, 237) | 237 (66, 504) |  | 7.23 (2.8,13.18) | 5.01 (1.4, 10.57) | -1.44 (-2.02, -0.85) |
| Ecuador | 0.01 (0, 0.02) | 0.02 (0.01, 0.03) |  | 94 (30, 190) | 317 (109, 625) |  | 3.94 (1.26,8.03) | 4.45 (1.52, 8.9) | -0.3 (-1.26, 0.68) |
| Egypt | 0.08 (0.03, 0.12) | 0.07 (0.03, 0.1) |  | 7312 (3244, 11999) | 12300 (4776, 21614) | | 54.09 (24.03,89.59) | 48.98 (19.95, 86.84) | 0.02 (-0.22, 0.27) |
| El Salvador | 0.05 (0.02, 0.09) | 0.04 (0.02, 0.07) |  | 337 (148, 576) | 473 (179, 861) |  | 22.07 (9.71,37.63) | 13.08 (4.98, 23.77) | -3.08 (-3.94, -2.2) |
| Equatorial Guinea | 0.01 (0, 0.02) | 0.03 (0.01, 0.06) |  | 10 (3, 23) | 57 (17, 159) |  | 9.57 (2.56,22.98) | 24.32 (7.38, 68.28) | 3.29 (1.22, 5.4) |
| Eritrea | 0.03 (0.01, 0.05) | 0.04 (0.02, 0.07) |  | 146 (44, 313) | 432 (132, 841) |  | 27.12 (8.8,57.06) | 31.12 (9.75, 60.27) | 0.8 (0.4, 1.21) |
| Estonia | 0.02 (0.01, 0.04) | 0.02 (0.01, 0.03) |  | 41 (14, 80) | 38 (12, 77) |  | 3.04 (1.05,5.95) | 2.03 (0.64, 4.16) | -1.8 (-2.3, -1.3) |
| Eswatini | 0.01 (0, 0.02) | 0.03 (0.01, 0.05) |  | 17 (5, 41) | 63 (20, 129) |  | 10.96 (3.02,26.83) | 19.94 (6.37, 41.04) | 3 (1.4, 4.62) |
| Ethiopia | 0.03 (0.01, 0.05) | 0.04 (0.02, 0.07) |  | 2577 (780, 5058) | 4667 (1955, 8230) | | 27.73 (8.99,54.51) | 25.39 (10.66, 44.3) | -0.69 (-1.75, 0.39) |
| Fiji | 0 (0, 0.01) | 0.01 (0, 0.02) |  | 3 (0, 10) | 9 (1, 23) |  | 1.87 (0.18,5.7) | 2.31 (0.33, 6.29) | 0.06 (-1.48, 1.63) |
| Finland | 0.01 (0, 0.02) | 0.01 (0, 0.02) |  | 84 (29, 162) | 140 (45, 289) |  | 1.89 (0.65,3.62) | 1.96 (0.63, 4.05) | -0.95 (-2.25, 0.36) |
| France | 0.06 (0.03, 0.09) | 0.04 (0.02, 0.07) |  | 6389 (2813, 10699) | 5251 (2170, 9492) | | 11.21 (4.92,18.64) | 5.61 (2.37, 10.14) | -2.37 (-2.78, -1.96) |
| Gabon | 0 (0, 0.01) | 0.02 (0.01, 0.03) |  | 9 (2, 27) | 50 (14, 101) |  | 3.06 (0.52,9.26) | 10.12 (2.94, 19.88) | 4.87 (2.34, 7.46) |
| Gambia | 0.02 (0.01, 0.03) | 0.04 (0.01, 0.06) |  | 20 (7, 44) | 126 (50, 254) |  | 12.68 (4.07,27.9) | 26.56 (10.32, 53.02) | 2.71 (1.78, 3.65) |
| Georgia | 0.06 (0.03, 0.1) | 0.06 (0.03, 0.1) |  | 531 (235, 993) | 613 (255, 1173) |  | 15 (6.66,27.68) | 16.34 (6.78, 30.69) | 1.86 (1.07, 2.66) |
| Germany | 0.03 (0.01, 0.05) | 0.03 (0.01, 0.05) |  | 7665 (3168, 13408) | 13980 (5718, 24264) | | 9.21 (3.84,16.05) | 12.73 (5.36, 21.75) | 1.04 (0.71, 1.38) |
| Ghana | 0.02 (0.01, 0.04) | 0.04 (0.02, 0.07) |  | 376 (139, 685) | 1695 (718, 2907) | | 11.83 (4.36,21.5) | 19.81 (8.31, 33.9) | 1.78 (0.3, 3.28) |
| Greece | 0.05 (0.02, 0.08) | 0.04 (0.02, 0.08) |  | 1437 (642, 2293) | 2503 (968, 4417) | | 17.06 (7.63,27.52) | 15.3 (6.4, 26.59) | 0.05 (-0.6, 0.7) |
| Greenland | 0.03 (0.01, 0.05) | 0.03 (0.01, 0.05) |  | 10 (4, 18) | 10 (4, 18) |  | 62.31 (22.52,113.9) | 32.42 (12.16, 59.47) | -2.76 (-3.63, -1.88) |
| Grenada | 0.02 (0, 0.04) | 0.01 (0, 0.02) |  | 2 (0, 4) | 1 (0, 3) |  | 3.61 (0.72,8.32) | 1.77 (0.2, 5.14) | -3.92 (-4.96, -2.86) |
| Guam | 0 (0, 0.01) | 0 (0, 0.01) |  | 0 (0, 2) | 0 (0, 2) |  | 0.6 (0,5.45) | 0.14 (0, 1.7) | -6.38 (-9.91, -2.71) |
| Guatemala | 0.03 (0.01, 0.05) | 0.02 (0.01, 0.04) |  | 216 (81, 381) | 420 (154, 814) |  | 14.37 (5.66,25.44) | 7.41 (2.73, 14.24) | -4.15 (-5.07, -3.22) |
| Guinea | 0.02 (0.01, 0.03) | 0.04 (0.02, 0.07) |  | 276 (98, 560) | 898 (364, 1688) |  | 16.82 (5.98,33.88) | 33.62 (13.8, 63.94) | 2.8 (1.6, 4.01) |
| Guinea-Bissau | 0.01 (0, 0.03) | 0.04 (0.02, 0.07) |  | 29 (9, 63) | 119 (46, 234) |  | 13.94 (4.46,30.08) | 30.85 (12.04, 59.47) | 3.28 (2.07, 4.51) |
| Guyana | 0 (0, 0) | 0 (0, 0.01) |  | 1 (0, 2) | 1 (0, 5) |  | 0.33 (0.05,1.04) | 0.39 (0.03, 1.53) | -0.28 (-2.11, 1.59) |
| Haiti | 0.04 (0.02, 0.08) | 0.03 (0.01, 0.06) |  | 501 (170, 1169) | 750 (242, 1795) |  | 31.03 (10.7,73.78) | 21.42 (6.81, 49.9) | -1.95 (-2.62, -1.27) |
| Honduras | 0.02 (0.01, 0.04) | 0.03 (0.01, 0.05) |  | 257 (92, 478) | 875 (266, 1826) |  | 24.78 (8.85,46.34) | 29.13 (9, 60.36) | -0.35 (-1.31, 0.61) |
| Hungary | 0.07 (0.03, 0.11) | 0.05 (0.02, 0.08) |  | 2711 (1207, 4423) | 2842 (1195, 4835) | | 32.05 (14.06,52.39) | 25.57 (10.26, 43.66) | -0.36 (-0.79, 0.07) |
| Iceland | 0.01 (0, 0.02) | 0.02 (0.01, 0.03) |  | 9 (3, 19) | 21 (7, 42) |  | 5.54 (1.67,11.36) | 6.69 (2.2, 13.06) | 0.48 (-0.19, 1.15) |
| India | 0.08 (0.03, 0.12) | 0.14 (0.07, 0.21) |  | 338715 (142558, 588856) | 1306231 (597797, 2109424) | | 192.12 (81.8,328.04) | 243.5 (110.52, 394.34) | 0.51 (0.25, 0.78) |
| Indonesia | 0.01 (0, 0.01) | 0.03 (0.01, 0.05) |  | 3354 (1061, 6828) | 20020 (8717, 35067) | | 7.07 (2.29,14.54) | 20.22 (8.66, 35.16) | 3.35 (2.1, 4.62) |
| Iran (Islamic Republic of) | 0.06 (0.03, 0.1) | 0.08 (0.04, 0.12) |  | 3974 (1732, 6637) | 11509 (5373, 18039) | | 36.77 (15.85,62.39) | 34.31 (15.93, 54.09) | -0.16 (-0.44, 0.13) |
| Iraq | 0.06 (0.03, 0.09) | 0.06 (0.03, 0.1) |  | 745 (316, 1276) | 1861 (829, 3111) | | 18.84 (8.02,32.38) | 16.68 (7.67, 27.3) | -0.4 (-0.58, -0.23) |
| Ireland | 0.02 (0.01, 0.04) | 0 (0, 0.01) |  | 421 (150, 786) | 97 (25, 229) |  | 17.87 (6.33,33.33) | 2.28 (0.59, 5.37) | -6.68 (-7.65, -5.69) |
| Israel | 0.09 (0.04, 0.14) | 0.07 (0.03, 0.1) |  | 850 (391, 1332) | 1141 (504, 1902) | | 32.83 (15.19,51.19) | 16.78 (7.44, 28.26) | -2.22 (-2.49, -1.95) |
| Italy | 0.08 (0.04, 0.13) | 0.07 (0.03, 0.11) |  | 11677 (5412, 18128) | 15001 (6989, 23664) | | 21.44 (9.94,33.41) | 14.82 (6.82, 23.66) | -1.46 (-1.94, -0.98) |
| Jamaica | 0.04 (0.02, 0.08) | 0.03 (0.01, 0.05) |  | 85 (37, 148) | 81 (31, 154) |  | 8.72 (3.74,15.27) | 4.94 (1.91, 9.69) | -3.27 (-4.19, -2.34) |
| Japan | 0.02 (0.01, 0.04) | 0.04 (0.02, 0.07) |  | 4228 (1713, 7371) | 10826 (4492, 19101) | | 4.4 (1.78,7.68) | 4.27 (1.79, 7.44) | -0.41 (-0.93, 0.12) |
| Jordan | 0.07 (0.03, 0.11) | 0.05 (0.02, 0.08) |  | 234 (95, 393) | 363 (162, 624) |  | 41.48 (16.97,69.22) | 14.15 (6.35, 24.16) | -4.52 (-5.14, -3.9) |
| Kazakhstan | 0.07 (0.03, 0.11) | 0.05 (0.02, 0.09) |  | 3610 (1549, 6027) | 3907 (1605, 6620) | | 47.04 (20.32,78.29) | 40.7 (16.72, 69.03) | -0.81 (-1.51, -0.1) |
| Kenya | 0.01 (0, 0.01) | 0.03 (0.01, 0.05) |  | 172 (47, 427) | 2036 (795, 4412) | | 4.35 (1.22,10.62) | 19.01 (7.34, 41.32) | 6.21 (4.06, 8.4) |
| Kiribati | 0.02 (0.01, 0.03) | 0.05 (0.02, 0.08) |  | 3 (1, 6) | 13 (5, 24) |  | 16.25 (5.78,31.48) | 36.22 (14.84, 63.17) | 2.8 (1.54, 4.08) |
| Kuwait | 0.04 (0.02, 0.07) | 0.04 (0.02, 0.07) |  | 29 (13, 47) | 97 (43, 166) |  | 13.76 (6.18,22.58) | 10.7 (4.68, 18.2) | -0.29 (-0.64, 0.06) |
| Kyrgyzstan | 0.11 (0.05, 0.16) | 0.09 (0.04, 0.14) |  | 2605 (1037, 4192) | 1332 (608, 2259) | | 140.67 (55.76,226.97) | 55.51 (25.39, 92.91) | -3.86 (-4.39, -3.33) |
| Lao People's Democratic Republic | 0.02 (0.01, 0.03) | 0.04 (0.02, 0.06) |  | 244 (79, 512) | 704 (285, 1363) |  | 23.19 (7.6,48.38) | 33.98 (13.93, 64.99) | 1.21 (0.36, 2.08) |
| Latvia | 0.02 (0.01, 0.04) | 0.02 (0.01, 0.04) |  | 100 (36, 181) | 67 (23, 144) |  | 4.23 (1.53,7.71) | 2.31 (0.74, 4.97) | -2.36 (-3.02, -1.69) |
| Lebanon | 0.07 (0.03, 0.11) | 0.04 (0.02, 0.07) |  | 352 (148, 615) | 513 (208, 994) |  | 33.32 (14.16,57.94) | 17.91 (7.28, 34.58) | -2.03 (-2.26, -1.81) |
| Lesotho | 0.02 (0.01, 0.04) | 0.04 (0.02, 0.07) |  | 143 (46, 330) | 408 (139, 817) |  | 26.63 (8.51,61.1) | 58.3 (20.34, 115.18) | 3.96 (2.77, 5.17) |
| Liberia | 0.01 (0, 0.01) | 0.03 (0.01, 0.05) |  | 18 (5, 40) | 158 (58, 323) |  | 3.47 (1,7.89) | 16.74 (6.24, 33.37) | 5.76 (3.65, 7.91) |
| Libya | 0.05 (0.02, 0.08) | 0.06 (0.02, 0.09) |  | 191 (80, 335) | 613 (272, 1043) |  | 22.52 (9.4,39.21) | 25.38 (11.35, 43.5) | 0.31 (-0.35, 0.97) |
| Lithuania | 0.03 (0.01, 0.05) | 0.01 (0, 0.03) |  | 272 (95, 501) | 79 (26, 162) |  | 9.61 (3.36,17.84) | 1.96 (0.65, 4.09) | -5.66 (-6.31, -5.01) |
| Luxembourg | 0.03 (0.01, 0.05) | 0.03 (0.01, 0.05) |  | 35 (13, 63) | 69 (27, 124) |  | 10.58 (3.93,18.78) | 11.68 (4.53, 20.95) | 0.52 (-0.01, 1.06) |
| Madagascar | 0 (0, 0.01) | 0.02 (0.01, 0.03) |  | 54 (12, 141) | 897 (295, 1894) |  | 2.13 (0.48,5.52) | 16.86 (5.68, 34.88) | 8.11 (5.63, 10.64) |
| Malawi | 0.01 (0, 0.02) | 0.02 (0.01, 0.04) |  | 106 (29, 241) | 469 (169, 929) |  | 5.5 (1.53,12.31) | 12.54 (4.51, 24.18) | 4.05 (2.5, 5.63) |
| Malaysia | 0.01 (0, 0.03) | 0.03 (0.01, 0.06) |  | 405 (129, 782) | 1848 (765, 3242) | | 9.55 (3.07,18.4) | 15.35 (6.38, 26.93) | 0.56 (-0.56, 1.69) |
| Maldives | 0.02 (0, 0.03) | 0.06 (0.03, 0.1) |  | 19 (5, 40) | 83 (37, 139) |  | 50.92 (13.74,109.23) | 67.71 (30.06, 112.85) | -0.09 (-0.83, 0.66) |
| Mali | 0.02 (0.01, 0.03) | 0.04 (0.02, 0.07) |  | 384 (125, 773) | 1532 (510, 3009) | | 18.06 (6.08,36.48) | 36.67 (12.99, 69.6) | 2.27 (0.96, 3.59) |
| Malta | 0.08 (0.04, 0.12) | 0.05 (0.02, 0.09) |  | 38 (17, 58) | 44 (18, 75) |  | 15.61 (7.16,23.97) | 7.97 (3.39, 13.53) | -3.24 (-3.88, -2.6) |
| Marshall Islands | 0 (0, 0) | 0 (0, 0) |  | 0 (0, 0) | 0 (0, 0) |  | 0.58 (0,5.55) | 0.07 (0, 1.26) | -5.95 (-9.58, -2.18) |
| Mauritania | 0.01 (0, 0.02) | 0.02 (0.01, 0.04) |  | 53 (13, 110) | 139 (50, 266) |  | 10.2 (2.57,21.02) | 14.17 (5.22, 27.12) | 1.14 (-0.13, 2.43) |
| Mauritius | 0 (0, 0) | 0 (0, 0) |  | 3 (0, 9) | 0 (0, 2) |  | 0.78 (0,2.34) | 0.03 (0, 0.2) | -11.92 (-15.26, -8.45) |
| Mexico | 0.12 (0.06, 0.17) | 0.05 (0.02, 0.08) |  | 14326 (7016, 21435) | 14980 (6258, 25229) | | 74.39 (36.54,111.74) | 25.34 (10.59, 42.66) | -4.05 (-4.32, -3.78) |
| Micronesia (Federated States of) | 0 (0, 0.01) | 0 (0, 0) |  | 1 (0, 4) | 0 (0, 1) |  | 2.57 (0,16.44) | 0.29 (0, 3.33) | -6.61 (-10.24, -2.84) |
| Monaco | 0.06 (0.02, 0.11) | 0.05 (0.02, 0.08) |  | 7 (2, 14) | 8 (3, 14) |  | 14.86 (3.61,29.77) | 13.42 (5.19, 24.06) | 0.8 (0.01, 1.6) |
| Mongolia | 0.06 (0.03, 0.1) | 0.05 (0.02, 0.08) |  | 252 (112, 422) | 180 (72, 317) |  | 44.8 (19.53,75.51) | 15.85 (6.46, 27.27) | -4.12 (-4.77, -3.46) |
| Montenegro | 0.05 (0.02, 0.09) | 0.05 (0.02, 0.08) |  | 24 (11, 44) | 31 (13, 53) |  | 7.11 (3.21,12.88) | 5.65 (2.39, 9.67) | -0.9 (-1.27, -0.53) |
| Morocco | 0.03 (0.01, 0.06) | 0.07 (0.03, 0.11) |  | 1015 (285, 2055) | 4606 (1921, 8181) | | 15.52 (4.47,30.73) | 31.32 (13.51, 54.15) | 2.83 (2.47, 3.18) |
| Mozambique | 0 (0, 0.01) | 0.02 (0.01, 0.03) |  | 61 (16, 143) | 452 (165, 929) |  | 2.17 (0.59,5.05) | 8.28 (3.02, 16.76) | 5.9 (4.09, 7.74) |
| Myanmar | 0.02 (0.01, 0.04) | 0.04 (0.02, 0.07) |  | 5695 (1771, 11457) | 12880 (4809, 23309) | | 49.35 (15.78,99.02) | 54.33 (20.15, 97.94) | -0.14 (-0.68, 0.4) |
| Namibia | 0.04 (0.02, 0.07) | 0.04 (0.02, 0.07) |  | 140 (50, 306) | 229 (84, 471) |  | 37.4 (13.28,81.28) | 30.19 (11.04, 61.78) | -0.33 (-1.1, 0.45) |
| Nauru | 0 (0, 0) | 0 (0, 0) |  | 0 (0, 0) | 0 (0, 0) |  | 0.38 (0,0) | 0.21 (0, 0) | -3.57 (-6.49, -0.57) |
| Nepal | 0.11 (0.05, 0.17) | 0.16 (0.08, 0.23) |  | 17919 (6682, 31547) | 52268 (22551, 85636) | | 436.26 (172.45,757.75) | 500.11 (218.51, 815.64) | 0.52 (0.35, 0.69) |
| Netherlands | 0.02 (0.01, 0.04) | 0.02 (0.01, 0.05) |  | 1108 (435, 2034) | 2691 (932, 5061) | | 9.14 (3.59,16.78) | 13.98 (4.85, 26.38) | 0.79 (-0.02, 1.6) |
| New Zealand | 0 (0, 0.01) | 0.01 (0, 0.02) |  | 52 (11, 125) | 207 (62, 438) |  | 2.37 (0.49,5.7) | 4.79 (1.44, 10.09) | 1.72 (0.46, 3) |
| Nicaragua | 0.02 (0.01, 0.03) | 0.02 (0.01, 0.04) |  | 37 (13, 72) | 214 (67, 401) |  | 4.84 (1.67,9.41) | 10.25 (3.19, 19.12) | 1.93 (0.96, 2.9) |
| Niger | 0.03 (0.01, 0.05) | 0.05 (0.02, 0.08) |  | 413 (161, 769) | 1462 (595, 2702) | | 32.59 (12.59,59.9) | 39.08 (16.03, 71.82) | 1.03 (-0.08, 2.15) |
| Nigeria | 0.04 (0.02, 0.07) | 0.06 (0.02, 0.09) |  | 5523 (2355, 9625) | 12741 (5490, 21977) | | 26.44 (11.22,46.43) | 31 (13.32, 52.86) | 0.91 (0.14, 1.69) |
| Niue | 0.01 (0, 0.03) | 0.01 (0, 0.03) |  | 0 (0, 1) | 0 (0, 0) |  | 17.3 (4.12,39.63) | 11.57 (2.85, 26.72) | -3.19 (-3.9, -2.48) |
| North Macedonia | 0.08 (0.04, 0.12) | 0.02 (0.01, 0.04) |  | 373 (173, 608) | 128 (49, 240) |  | 42.05 (19.42,69.03) | 8.09 (3.06, 15.2) | -4.96 (-5.96, -3.95) |
| Northern Mariana Islands | 0.03 (0.01, 0.06) | 0.07 (0.03, 0.11) |  | 2 (1, 4) | 7 (3, 13) |  | 27.46 (7.58,57.02) | 30.98 (12.38, 57.17) | -0.4 (-1.16, 0.36) |
| Norway | 0.02 (0.01, 0.04) | 0.02 (0.01, 0.03) |  | 253 (105, 456) | 406 (121, 811) |  | 6.23 (2.52,11.16) | 7.51 (2.17, 15.08) | 0.11 (-1.05, 1.28) |
| Oman | 0.07 (0.03, 0.11) | 0.06 (0.03, 0.1) |  | 115 (47, 205) | 165 (69, 285) |  | 43.34 (17.97,77.72) | 30.13 (12.46, 51.91) | -0.55 (-0.92, -0.18) |
| Pakistan | 0.08 (0.04, 0.12) | 0.1 (0.05, 0.16) |  | 23732 (10137, 42316) | 58245 (26020, 94436) | | 102.01 (43.28,184.03) | 128.66 (57.78, 208.2) | 0.73 (0.52, 0.94) |
| Palau | 0 (0, 0.01) | 0 (0, 0.01) |  | 0 (0, 1) | 0 (0, 1) |  | 3.41 (0,22.44) | 1.18 (0, 9.56) | -3.22 (-6.35, 0.02) |
| Palestine | 0.08 (0.04, 0.12) | 0.05 (0.02, 0.09) |  | 164 (69, 282) | 197 (87, 352) |  | 36.61 (15.01,62.5) | 18.09 (8.1, 32.65) | -2.72 (-2.89, -2.56) |
| Panama | 0.03 (0.01, 0.05) | 0.02 (0.01, 0.04) |  | 64 (25, 116) | 134 (41, 287) |  | 9.01 (3.47,16.27) | 5.98 (1.84, 12.91) | -3.53 (-4.76, -2.29) |
| Papua New Guinea | 0 (0, 0) | 0 (0, 0) |  | 16 (3, 49) | 21 (3, 61) |  | 1.92 (0.36,5.74) | 1.01 (0.14, 3) | -3.61 (-6.58, -0.54) |
| Paraguay | 0.02 (0.01, 0.04) | 0.04 (0.02, 0.07) |  | 77 (30, 147) | 247 (95, 462) |  | 6.72 (2.58,12.68) | 8.59 (3.31, 16.11) | 0.97 (-0.1, 2.06) |
| Peru | 0 (0, 0.01) | 0.02 (0.01, 0.03) |  | 92 (28, 206) | 547 (200, 1060) |  | 1.57 (0.48,3.47) | 3.22 (1.18, 6.23) | 2.22 (1.21, 3.24) |
| Philippines | 0 (0, 0.01) | 0 (0, 0.01) |  | 379 (104, 827) | 574 (164, 1328) |  | 2.99 (0.82,6.52) | 1.51 (0.44, 3.49) | -1.84 (-3.38, -0.27) |
| Poland | 0.04 (0.02, 0.07) | 0.04 (0.02, 0.06) |  | 3179 (1372, 5344) | 3630 (1504, 6214) | | 12.21 (5.26,20.55) | 8.52 (3.54, 14.62) | -1.21 (-1.57, -0.85) |
| Portugal | 0.02 (0.01, 0.04) | 0.03 (0.01, 0.05) |  | 724 (300, 1288) | 1400 (554, 2551) | | 9.11 (3.7,16.09) | 8.09 (3.18, 14.56) | 0.46 (-0.65, 1.58) |
| Puerto Rico | 0.01 (0, 0.03) | 0 (0, 0.01) |  | 87 (28, 178) | 40 (6, 98) |  | 4.47 (1.45,9.14) | 0.9 (0.14, 2.24) | -7.82 (-9.62, -5.99) |
| Qatar | 0.07 (0.03, 0.11) | 0.06 (0.03, 0.1) |  | 20 (8, 35) | 67 (30, 122) |  | 58.22 (24.56,103.1) | 57.57 (26.94, 100.55) | 0.31 (0.03, 0.58) |
| Republic of Korea | 0.05 (0.02, 0.09) | 0.08 (0.04, 0.12) |  | 2512 (903, 4465) | 6526 (3045, 10493) | | 18.08 (6.28,32.49) | 12.61 (5.89, 20.27) | -2.78 (-3.95, -1.58) |
| Republic of Moldova | 0.07 (0.03, 0.11) | 0.03 (0.01, 0.05) |  | 1156 (438, 1914) | 216 (84, 410) |  | 46.01 (17.5,76.44) | 6.07 (2.35, 11.55) | -7.29 (-8.02, -6.57) |
| Romania | 0.08 (0.03, 0.12) | 0.01 (0, 0.03) |  | 6891 (2597, 11348) | 731 (249, 1458) |  | 47.28 (17.46,77.5) | 3.25 (1.12, 6.43) | -8.52 (-9.06, -7.97) |
| Russian Federation | 0.05 (0.02, 0.08) | 0.02 (0.01, 0.04) |  | 21747 (7759, 36939) | 6964 (2693, 12868) | | 18.33 (6.53,31.15) | 4.58 (1.76, 8.45) | -4.93 (-5.32, -4.54) |
| Rwanda | 0.02 (0.01, 0.04) | 0.05 (0.02, 0.08) |  | 459 (139, 895) | 1267 (521, 2558) | | 29.17 (9.1,55.97) | 39.53 (16.39, 77.27) | 1.26 (-0.55, 3.1) |
| Saint Kitts and Nevis | 0.02 (0, 0.06) | 0 (0, 0) |  | 1 (0, 3) | 0 (0, 0) |  | 5.28 (0.26,16.27) | 0 (0, 0) | -25.31 (-28.74, -21.71) |
| Saint Lucia | 0.01 (0, 0.05) | 0 (0, 0.01) |  | 2 (0, 7) | 0 (0, 3) |  | 3.81 (0,15.03) | 0.19 (0, 2.33) | -10.97 (-12.46, -9.45) |
| Saint Vincent and the Grenadines | 0.01 (0, 0.04) | 0 (0, 0.01) |  | 1 (0, 2) | 0 (0, 1) |  | 1.53 (0,5.69) | 0.13 (0, 1.59) | -9.9 (-11.47, -8.31) |
| Samoa | 0.02 (0, 0.06) | 0.02 (0, 0.03) |  | 11 (0, 43) | 13 (4, 29) |  | 25.77 (0,97.48) | 18.36 (5.01, 39.29) | -2.65 (-3.29, -2) |
| San Marino | 0.07 (0.03, 0.12) | 0.05 (0.02, 0.09) |  | 4 (1, 6) | 6 (2, 10) |  | 18.79 (7.32,31.83) | 13.76 (5.39, 25.75) | -0.55 (-1.24, 0.15) |
| Sao Tome and Principe | 0.01 (0, 0.03) | 0.03 (0.01, 0.06) |  | 4 (1, 13) | 20 (6, 41) |  | 13.42 (2.29,39.91) | 40.01 (12.74, 80.34) | 4.13 (2.81, 5.47) |
| Saudi Arabia | 0.08 (0.03, 0.12) | 0.07 (0.03, 0.11) |  | 1304 (541, 2270) | 2573 (1127, 4395) | | 57.67 (23.78,100.9) | 40.19 (17.8, 66.57) | -1.27 (-1.49, -1.05) |
| Senegal | 0.02 (0.01, 0.03) | 0.03 (0.01, 0.06) |  | 176 (60, 370) | 732 (312, 1316) |  | 11.45 (3.98,24.03) | 19.88 (8.73, 35.84) | 2.31 (1.26, 3.37) |
| Serbia | 0.07 (0.03, 0.12) | 0.02 (0.01, 0.04) |  | 1742 (778, 2905) | 669 (247, 1216) |  | 30.53 (13.83,51.43) | 7.68 (2.82, 14) | -4.46 (-5.16, -3.75) |
| Seychelles | 0 (0, 0.02) | 0.01 (0, 0.05) |  | 0 (0, 3) | 2 (0, 12) |  | 0.57 (0,8.16) | 4.12 (0, 21.02) | 6.57 (4.07, 9.14) |
| Sierra Leone | 0.01 (0.01, 0.03) | 0.03 (0.01, 0.05) |  | 101 (33, 207) | 415 (161, 797) |  | 11.17 (3.67,22.61) | 23.78 (9.2, 45.34) | 3.4 (2.16, 4.66) |
| Singapore | 0.02 (0, 0.06) | 0.04 (0.01, 0.08) |  | 99 (3, 297) | 214 (57, 474) |  | 9.35 (0.28,28.11) | 5.33 (1.43, 11.8) | -2.41 (-4.24, -0.55) |
| Slovakia | 0.06 (0.03, 0.09) | 0.04 (0.02, 0.07) |  | 429 (193, 756) | 428 (196, 743) |  | 12.48 (5.66,21.72) | 7.98 (3.59, 13.99) | -0.84 (-1.31, -0.37) |
| Slovenia | 0.07 (0.03, 0.11) | 0.06 (0.03, 0.1) |  | 272 (117, 472) | 280 (125, 477) |  | 17.88 (7.56,31.01) | 9.84 (4.41, 17.12) | -3.11 (-3.51, -2.71) |
| Solomon Islands | 0 (0, 0) | 0 (0, 0) |  | 1 (0, 3) | 1 (0, 4) |  | 1.13 (0,4.57) | 0.55 (0, 2.93) | -2.71 (-5.59, 0.26) |
| Somalia | 0 (0, 0.01) | 0.02 (0.01, 0.04) |  | 56 (14, 146) | 739 (226, 1746) |  | 4.5 (1.14,11.08) | 21.21 (6.61, 49.26) | 5.17 (3.88, 6.47) |
| South Africa | 0.01 (0, 0.02) | 0.03 (0.01, 0.05) |  | 1276 (482, 2426) | 4450 (1849, 7754) | | 10.81 (4.06,20.57) | 17.96 (7.46, 31.14) | 1.99 (1.15, 2.83) |
| South Sudan | 0.04 (0.02, 0.07) | 0.03 (0.01, 0.05) |  | 292 (99, 592) | 258 (97, 546) |  | 28.89 (9.97,57.11) | 16.06 (6.06, 32.77) | -1.68 (-2.47, -0.89) |
| Spain | 0.04 (0.02, 0.06) | 0.06 (0.03, 0.09) |  | 4126 (1702, 7062) | 10309 (4358, 17312) | | 12.44 (5.16,21.44) | 14.23 (6.08, 23.83) | 0.32 (-0.06, 0.7) |
| Sri Lanka | 0 (0, 0.01) | 0.03 (0.01, 0.05) |  | 123 (32, 281) | 1588 (533, 3057) | | 2.56 (0.68,5.83) | 11.96 (4.12, 22.81) | 4.57 (3.61, 5.53) |
| Sudan | 0.03 (0.01, 0.05) | 0.05 (0.02, 0.08) |  | 961 (318, 2218) | 2308 (895, 4650) | | 21.97 (7.36,49.39) | 27.7 (10.79, 56.76) | 1.04 (0.43, 1.65) |
| Suriname | 0 (0, 0) | 0 (0, 0.01) |  | 1 (0, 2) | 2 (0, 6) |  | 0.49 (0.09,1.22) | 0.63 (0.09, 1.91) | -0.37 (-2.23, 1.52) |
| Sweden | 0.01 (0.01, 0.03) | 0.01 (0, 0.03) |  | 364 (138, 666) | 750 (233, 1503) |  | 4.11 (1.57,7.5) | 6.06 (1.92, 12.2) | 0.86 (-0.21, 1.95) |
| Switzerland | 0.06 (0.02, 0.09) | 0.05 (0.02, 0.08) |  | 913 (408, 1477) | 1357 (591, 2280) | | 14.21 (6.31,22.88) | 13.13 (5.65, 21.97) | -0.01 (-0.31, 0.29) |
| Syrian Arab Republic | 0.07 (0.03, 0.11) | 0.06 (0.03, 0.1) |  | 1036 (418, 1780) | 1686 (695, 3067) | | 43.18 (17.15,78.01) | 34.26 (14.07, 62.62) | -0.95 (-1.12, -0.77) |
| Taiwan (Province of China) | 0.03 (0.01, 0.05) | 0.04 (0.02, 0.07) |  | 932 (358, 1645) | 1812 (724, 3493) | | 15.28 (5.85,27.06) | 8.18 (3.27, 15.79) | -2.4 (-3.16, -1.64) |
| Tajikistan | 0.12 (0.06, 0.18) | 0.09 (0.04, 0.13) |  | 1755 (830, 2771) | 1444 (636, 2594) | | 110.53 (51.99,173.86) | 59.15 (24.86, 110.01) | -2.17 (-2.49, -1.85) |
| Thailand | 0.01 (0, 0.02) | 0.04 (0.02, 0.07) |  | 1903 (643, 3917) | 7355 (3012, 12820) | | 10.78 (3.62,22.13) | 13.47 (5.53, 23.51) | 0.2 (-0.56, 0.98) |
| Timor-Leste | 0.01 (0, 0.02) | 0.02 (0.01, 0.04) |  | 14 (4, 33) | 61 (18, 139) |  | 10.68 (2.83,25.2) | 16.12 (4.78, 36.66) | 0.52 (-1.04, 2.11) |
| Togo | 0.03 (0.01, 0.05) | 0.06 (0.03, 0.09) |  | 162 (61, 304) | 736 (308, 1334) |  | 25.7 (9.78,48.45) | 37.51 (15.62, 67.82) | 1.71 (0.37, 3.07) |
| Tokelau | 0.02 (0, 0.03) | 0.01 (0, 0.03) |  | 0 (0, 0) | 0 (0, 0) |  | 29.6 (7.25,67.47) | 16.68 (3.95, 36.82) | -3.5 (-4.18, -2.81) |
| Tonga | 0.01 (0, 0.03) | 0.02 (0, 0.04) |  | 3 (1, 6) | 3 (1, 7) |  | 9.64 (2.17,22.72) | 7.05 (1.37, 17.02) | -2.57 (-3.24, -1.9) |
| Trinidad and Tobago | 0.01 (0, 0.03) | 0 (0, 0.01) |  | 7 (1, 20) | 5 (0, 15) |  | 1.71 (0.22,4.74) | 0.47 (0.01, 1.6) | -5.98 (-7.13, -4.81) |
| Tunisia | 0.07 (0.03, 0.11) | 0.06 (0.03, 0.1) |  | 613 (259, 1115) | 1269 (544, 2270) | | 27.62 (11.86,49.6) | 20.51 (8.82, 36.79) | -1.33 (-1.65, -1) |
| Turkey | 0.07 (0.03, 0.11) | 0.06 (0.03, 0.09) |  | 10027 (4305, 16775) | 15367 (6176, 26399) | | 57 (24.66,95.04) | 33.74 (13.53, 58.15) | -1.48 (-2, -0.96) |
| Turkmenistan | 0.08 (0.04, 0.12) | 0.05 (0.02, 0.09) |  | 587 (223, 1011) | 280 (103, 775) |  | 54.41 (21.1,93.15) | 13.34 (4.87, 37.74) | -5.5 (-6.17, -4.83) |
| Tuvalu | 0 (0, 0.01) | 0 (0, 0.01) |  | 0 (0, 1) | 0 (0, 1) |  | 3.71 (0,15.37) | 4.42 (0, 14.92) | -0.08 (-1.78, 1.66) |
| Uganda | 0.02 (0.01, 0.04) | 0.03 (0.01, 0.06) |  | 524 (164, 1110) | 1489 (571, 3101) | | 17.1 (5.55,35.35) | 20.73 (8.12, 42.62) | 0.67 (-1.31, 2.69) |
| Ukraine | 0.06 (0.03, 0.1) | 0.03 (0.01, 0.05) |  | 18021 (6595, 30428) | 2877 (1056, 7204) | | 38.56 (13.97,64.92) | 5.92 (2.18, 14.66) | -7.39 (-7.7, -7.07) |
| United Arab Emirates | 0.08 (0.04, 0.13) | 0.06 (0.03, 0.1) |  | 106 (43, 200) | 479 (195, 875) |  | 81.74 (33.47,162.18) | 45.41 (20.29, 81.97) | -1.42 (-2.51, -0.31) |
| United Kingdom | 0.02 (0.01, 0.03) | 0.01 (0, 0.02) |  | 4833 (1689, 9058) | 3895 (1127, 8269) | | 8.88 (3.11,16.57) | 5.35 (1.54, 11.34) | -0.59 (-1.52, 0.34) |
| United Republic of Tanzania | 0.01 (0, 0.01) | 0.02 (0.01, 0.04) |  | 172 (53, 361) | 1371 (520, 2597) | | 3.27 (1.02,6.78) | 11.44 (4.35, 21.43) | 5.37 (3.53, 7.25) |
| United States of America | 0.05 (0.02, 0.08) | 0.04 (0.02, 0.06) |  | 67128 (30523, 107818) | 98947 (38793, 168370) | | 36.02 (16.27,57.56) | 31.53 (12.49, 53.72) | -0.59 (-1.18, 0.01) |
| United States Virgin Islands | 0.01 (0, 0.03) | 0 (0, 0) |  | 1 (0, 3) | 0 (0, 1) |  | 2.95 (0.83,6.46) | 0.19 (0, 0.63) | -11.36 (-13.46, -9.21) |
| Uruguay | 0.01 (0, 0.02) | 0.01 (0, 0.03) |  | 56 (16, 126) | 183 (48, 397) |  | 2.56 (0.73,5.77) | 5.65 (1.49, 12.19) | 2.56 (1.98, 3.15) |
| Uzbekistan | 0.1 (0.05, 0.15) | 0.07 (0.03, 0.11) |  | 3733 (1495, 6244) | 2569 (1119, 4846) | | 57.31 (23.06,95.81) | 28.23 (12.05, 57.24) | -3.55 (-4.18, -2.92) |
| Vanuatu | 0 (0, 0.01) | 0 (0, 0.01) |  | 2 (0, 5) | 5 (1, 14) |  | 5.76 (0.67,17.17) | 5.91 (0.83, 17.32) | -0.73 (-2.33, 0.89) |
| Venezuela (Bolivarian Republic of) | 0.02 (0.01, 0.04) | 0.02 (0.01, 0.04) |  | 386 (148, 718) | 1216 (442, 2224) | | 8.06 (3.08,14.95) | 7.98 (2.92, 14.53) | -1.53 (-2.45, -0.61) |
| Viet Nam | 0.02 (0.01, 0.03) | 0.03 (0.01, 0.05) |  | 2445 (598, 5247) | 6740 (1560, 13525) | | 10.92 (2.67,23.45) | 13.85 (3.17, 27.71) | 1.36 (0.63, 2.1) |
| Yemen | 0.03 (0.01, 0.06) | 0.06 (0.03, 0.09) |  | 784 (266, 1688) | 2800 (1086, 5071) | | 31.81 (11.27,70.17) | 42.73 (17.07, 77.01) | 1.23 (0.8, 1.66) |
| Zambia | 0.02 (0.01, 0.04) | 0.03 (0.01, 0.06) |  | 255 (87, 509) | 725 (275, 1354) |  | 19.87 (6.91,38.48) | 22.23 (8.34, 40.7) | 1.05 (0.06, 2.06) |
| Zimbabwe | 0.01 (0, 0.02) | 0.02 (0.01, 0.05) |  | 145 (45, 295) | 562 (177, 1120) |  | 8.02 (2.55,16.04) | 16.44 (5.24, 32.83) | 4.94 (3.28, 6.64) |

# Table S4. Association between country-level factors and DALY rate of COPD attributable to ozone pollution

| Country-level factors | Men | | |  | Women | | |
| --- | --- | --- | --- | --- | --- | --- | --- |
|  | β | 95% CI | P value |  | β | 95% CI | P value |
| Lifestyle factors |  |  |  |  |  |  |  |
| Smoking prevalence (%) | 0.803 | 0.691, 0.916 | <0.001 |  | 0.54 | 0.390, 0.690 | <0.001 |
| Demographic factors |  |  |  |  |  |  |  |
| Average years of schooling (years) | -1.938 | -2.655, -1.221 | <0.001 |  | -0.63 | -1.170, -0.090 | 0.02 |
| Population density (thousand people/km^2^) | 5.075 | 2.738, 7.413 | <0.001 |  | 2.808 | 1.019, 4.597 | 0.002 |
| Proportion of population aged 65 years and above (%) | 3.115 | 2.717, 3.513 | <0.001 |  | 1.23 | 0.887, 1.574 | <0.001 |
| Socioeconomic factors |  |  |  |  |  |  |  |
| Proportion of urban population (%) | -0.379 | -0.465, -0.293 | <0.001 |  | -0.317 | -0.383, -0.251 | <0.001 |
| GDP per capita (US$ (thousand)) | -0.043 | -0.144, 0.057 | 0.4 |  | -0.015 | -0.092, 0.061 | 0.696 |
| Environmental factors |  |  |  |  |  |  |  |
| Average maximum temperature (℃) | 3.598 | 1.916, 5.281 | <0.001 |  | 1.983 | 6.996, 3.266 | 0.002 |
| Average temperature (℃) | -5.297 | -8.134, -2.460 | <0.001 |  | -3.771 | -5.950, -1.593 | 0.001 |
| Average minimum temperature (℃) | 1.81 | 0.356, 3.265 | 0.015 |  | 1.911 | 0.775, 3.047 | 0.001 |
| Rainfall (mm) | 0.027 | 0.001, 0.002 | <0.001 |  | 0.016 | 0.006, 0.026 | 0.001 |
| NVDI | -511.03 | -593.832, -428.223 | <0.001 |  | -400.14 | -464.80, -335.477 | <0.001 |
